# Subsistence and population development from the Middle Neolithic B (2800–2350 BCE) to the Late Neolithic (2350–1700 BCE) in Southern Scandinavia

**DOI:** 10.1371/journal.pone.0301938

**Published:** 2024-10-28

**Authors:** Jens Winther Johannsen, Julian Laabs, Magdalena M. E. Bunbury, Morten Fischer Mortensen

**Affiliations:** 1 ROMU/Frederikssund Museum, Jægerspris, Denmark; 2 Department of History, Leipzig University, Leipzig, Germany; 3 CRC 1266 “Scales of Transformation”, Kiel University, Kiel, Germany; 4 ARC Centre of Excellence for Australian Biodiversity and Heritage, College of Arts, Society and Education, James Cook University, Brisbane, Australia; 5 ROOTS, Kiel University, Kiel, Germany; 6 National Museum of Denmark, Copenhagen, Denmark; University of Bern, Institute of Forensic Medicine, SWITZERLAND

## Abstract

The present study aims to explore the hypothesis of a link between a population increase derived from intensified food production and the development from the widespread cultural diversity of the Middle Neolithic B (MNB) to the cultural unity towards the end of the Late Neolithic (LN) in Southern Scandinavia. We explore this through quantitative modelling of radiocarbon dates, aoristic time series of material culture and palynological data. On this basis, we propose three main results that may explain the transformation: (1) A supra-regional population increase, culminating in the middle of the LN (c. 2100 BCE). (2) A depopulation in Western Jutland at the transition from the MNB to the LN (c. 2400–2300 BCE) counterbalanced by a population increase in North and East Denmark. (3) A population boom in Southern Sweden around 2250–2000 BCE, possibly leading to migrations towards west. Furthermore, we propose an overall decline in population in the final LN (c. 1850 BCE).

## Introduction

The transition from the Middle Neolithic B (MNB, 2850–2350 BCE) to the Late Neolithic (LN, 2350–1700 BCE) in Southern Scandinavia is recognised as a time of major cultural shift. Roughly put, cultural differences between regions in the MNB were replaced by cultural unity in the LN, primarily recognised by the widespread adaption of bifacial flint work, daggers in particular [1,2]. This transformation has recently been suggested to have been more prolonged than hitherto thought, and subsistence changes, population increase and migrations are placed as possible drivers [3]. The present study further explores the transformative developments from the MNB to LN through a socioecological lens, emphasizing the significance of agricultural technologies and subsistence strategies.

We propose that these factors serve as potential prerequisites for a population boom during the LN. The foundational model regarding the relationship between agricultural productivity and population growth/size is rooted in Malthus’ concept that heightened agricultural productivity increases population [4–6]. Malthus’ overarching theory, asserting that population tends to outpace the carrying capacity of a landscape to produce sufficient food, resulting in cyclical patterns of population collapse and readjustment, but especially his socio-political counter measures, has been subject of debate for over two centuries. However, the fundamental assumption of the relationship between agricultural productivity and population growth has not been outright refuted and is still the base of many models of population dynamics [5,7–9]. Therefore, we adopt the viewpoint that advances in technology and subsistence strategies can multiply agricultural productivity and increase the carrying capacity of a landscape for human population, which in ecology is defined as the maximum population density that an area can sustain without being degraded or destroyed [10,11]. A well-known example of such advances is the widespread Neolithisation processes, understood here as the transition from a subsistence mainly based on hunting, fishing and gathering to one based mainly on agriculture and livestock farming [12–14]. These are accompanied by a marked increase in population size due to increased birth rates and fertility values supported by a sedentary lifestyle, known as the Neolithic Demographic Transition [15]. In an already established farming economy, a landscape’s carrying capacity can be further increased through improvements in agricultural techniques and strategies [7]. An example is the Secondary Products Revolution, where the exploitation of domestic animals was extended to include their secondary products (milk, wool, traction and manure), which is suggested to have led to population booms in the Middle East and Europe in the 4th and 3rd millennia BCE [16–18]. Another more recent example is the so-called Green Revolution in the early 1960s, where the introduction of new high-yield crops, chemical fertilisers and mechanisation of labour tasks was followed by a massive population boom, particularly in developing countries [19].

For the Southern part of Scandinavia, the transition from foraging to agro-pastoral subsistence dates to around 4000 BCE [20–23]. This shift is also visible in the archaeodemographic record, indicating exponential population growth from the Late Mesolithic to the Early Neolithic [24–28]. However, the progress in the subsequent millennia deviated significantly from a linear advancement regarding agricultural techniques. In particular the second half of the Middle Neolithic, the MNB, has been described as a period of “de-Neolithisation”, where foraging strategies were reintroduced in some areas [29–32] while pastoral component to subsistence was intensified in other areas [33,34]. This is in contrast to the following period, the Late Neolithic, or LN, which in recent studies has been characterised as a period with a strong revival of the agricultural economy, a “re-Neolithisation”, which is likely to have led to a population increase [3,24,35–37].

Population increase is a well-known driver of migrations and may lead to expansion of people and thereby genes, and new cultural habits [20,38,39]. We thus hypothesise that increased food production and derived population growth were central in the change from cultural diversity in the MNB to cultural unity towards the end of the LN. While research on population dynamics in Neolithic Northern Europe [24,26,29,40] supports an overall population increase in the LN, the population development from MNB to LN in Southern Scandinavia remains poorly understood, particularly on the regional level. Our goal is thus to investigate population dynamics in MNB-LN on both regional and cross-regional levels and, on this basis, to be able to discuss the gradual spread of people and new cultural traits across Southern Scandinavia.

To investigate the spatio-temporal behaviour of population dynamics, we employ a multi-proxy approach utilizing archaeological and palaeoenvironmental data. More specifically, distributions of radiocarbon dates, density of graves with battle axes and flint daggers and the degree of vegetation openness. These proxies were selected based on the assumption that an increase in population density correlates with increased human activity and its associated impact, which in turn is reflected in increased rates of radiocarbon dates, artefact density and landscape openness. Because this relation is not always straightforward, we use more than one proxy to approach our hypothesis.

## Environmental and archaeological background

### Environment

The geographical delimitation of our study corresponds to present-day Denmark, Scania, Southern Sweden and Schleswig-Holstein, Germany, which we group as Southern Scandinavia. Geologically, Scania, the Danish islands, and the eastern parts of Jutland are a moraine landscape with a dense concentration of arable land. However, the southwestern part of Jutland was formed by melting ice during the Weichselian glaciation and is therefore characterised by sandy and nutrient-poor soils [41,42]. The area under study is relatively homogeneous regarding climate conditions, belonging to the nemoral zone with warm summers and mild winters [43]. The Neolithisation of Southern Scandinavia at 4000 BCE happened during the Holocene Thermal Maximum, a period of generally warm weather in the region [24,27]. It was however interrupted by a period of long-term cooling, commonly known as the 4.2 ka BP climate event, whose duration varied from region to region [24].

### Archaeology

#### The Early Neolithic and the Middle Neolithic A

During the Early and the first half of the Middle Neolithic (c. 4000-2800/2600 BCE), the cultural landscape in Southern Scandinavia was shaped by Funnel Beaker groups. These were pioneering farmers in Northern Europe, sharing cultural connections, notably through the tradition of megalith building [20,44]. As a result, our study area can be characterised as relatively culturally homogeneous during this period. The Funnel Beaker economy is thought to have been based mainly on a combination of cereal cultivation and animal husbandry [20]. However, pastoral elements seem to have been increased in the northern part of Jutland towards the end of the period, likely related to the integration of this area into the Globular Amphora network [45].

#### The Middle Neolithic B

**Fig 1:**
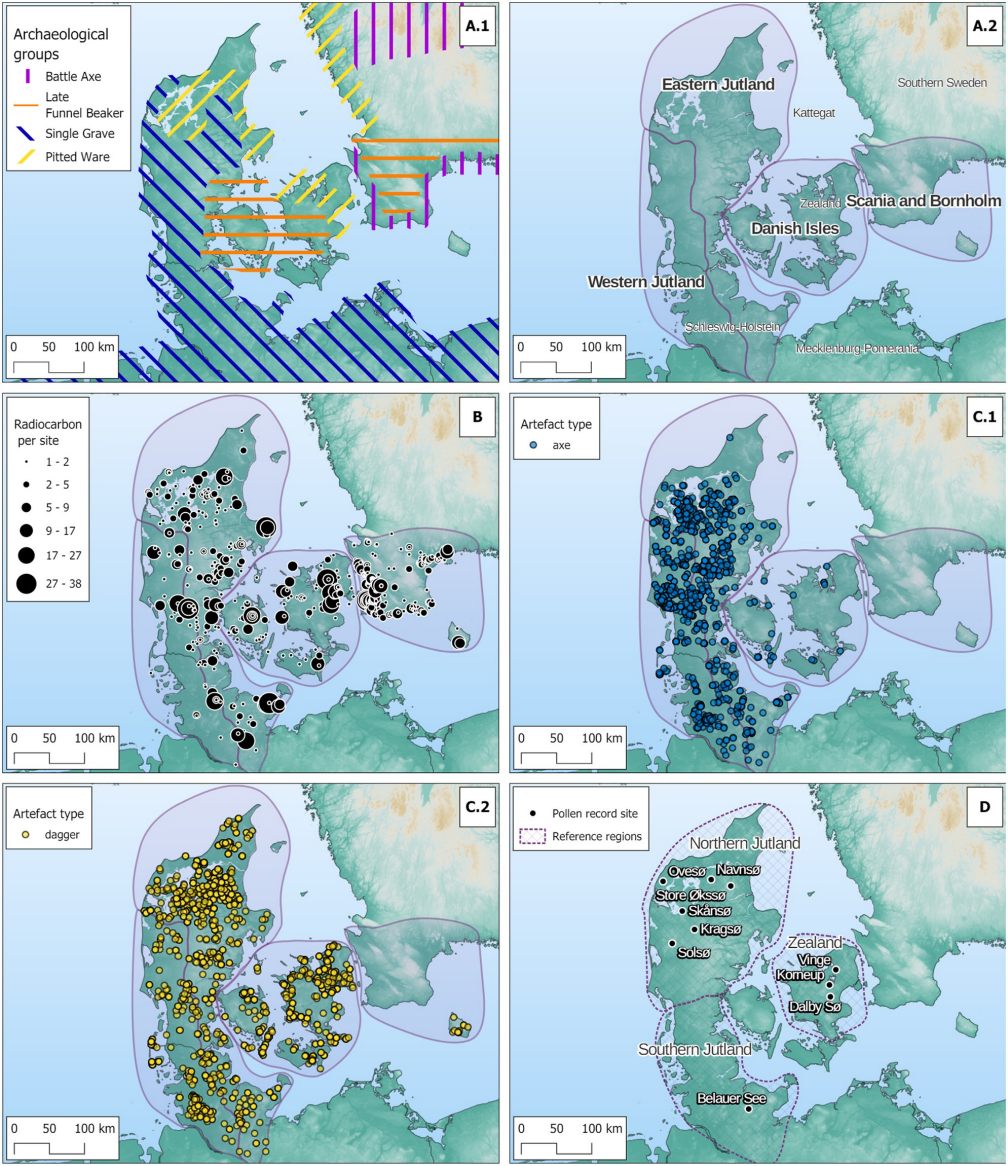
Maps of the study area. A.1) The rough distribution of the various cultural groupings in MNB (Battle Axe Culture c. 2800-2250/2200 BCE; Late Funnel Beaker Culture, Store Valby phase c. 3000–2600 BCE; Single Grave Culture c. 2800-2250/2200 BCE; Pitted Ware Culture c. 3100–2700 BCE) [57–60]. A.2) Distribution of the four cultural-geographical regions discussed B) Distribution of sites with radiocarbon dates and number of dates per site. C.1) Distribution of graves with battle axes C.2) Distribution of graves with flint daggers. D) Distribution of pollen record sites and regional division for reference. Basemap source: https://doi.org/10.5270/ESA-c5d3d65.

**Fig 2:**
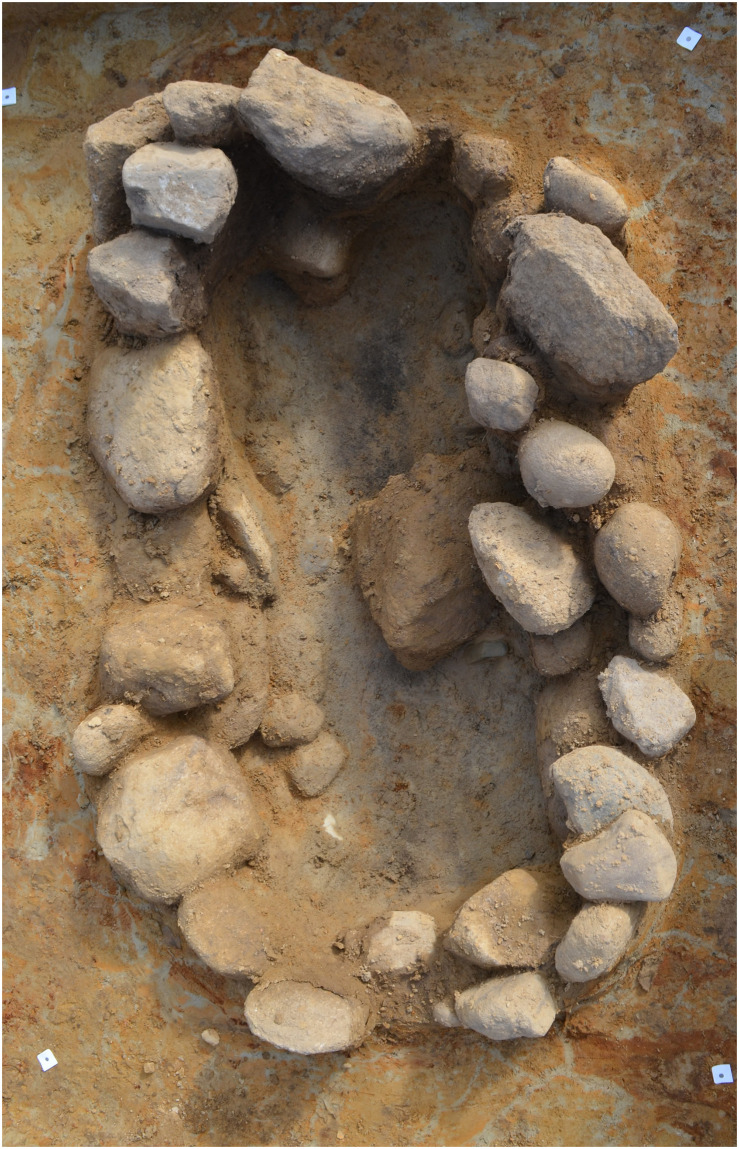
Grave typical of the Single Grave Culture, including battle axe of Glob’s type C. The battle axe is seen just below the large stone in the centre of the grave. The central oblong, dark patch are putrefaction fluid deposits forming a vague silhouette of the buried person. The grave, about two metres long and oriented east-west, was excavated near Esbjerg in Southwest Jutland (grave A6792; SJM 979 Veldbæk Industri). Photo: Sydvestjyske Museer.

The following period, the MNB (c. 2800–2350 BCE), was characterised by regional differences in cultural practices and subsistence patterns (see Fig 1, A.1): As part of the large-scale westward population movements associated with the Yamnaya Culture from the Pontic-Caspian steppe and the derived genetic mixing with European Neolithic populations during the 3rd millennium, people belonging to the Corded Ware Complex migrated into Western Jutland (here the Single Grave Culture) and Scania (here the Battle Axe Culture) around 2800 BCE and remained until 2250/2200 BCE [46–49] (see Fig 1, A.1). The Corded Ware groups are mainly recognised by their characteristic pottery and shaft hole axes, and by their single burial tradition, which in Jutland were covered by small mounds (Fig 2) [50–52]. Little is known of the subsistence of the Scandinavian Corded Ware groups, but the vague settlement remains, and the extensive clearance of the forests with fire, thereby creating an open, steppelike area in Western Jutland, has been attributed to an economy based on pastoralism [33,35,53,54]. However, studies show that the importance of cereal cultivation increased towards the end of the MNB, with naked barley as the most important crop, while emmer and bread wheat were also cultivated [55,56].

Partly contemporary with the Corded Ware groups, the Pitted Ware Culture influenced the Kattegat region (c. 3100–2700 BCE [61], see Fig 1, A.1), at least partly through migration from the Scandinavian Peninsula or neighbouring islands [62]. It has recently been shown that cereal cultivation was part of the economy on both West Swedish and Danish Pitted Ware sites [63], and cereal cultivation even spread to Middle Sweden and Finland during the Middle Neolithic [64]. Nevertheless, the preference for coastal settlements and the finds of numerous tanged arrowheads and bones from game animals indicate a decline in the importance of agriculture and a corresponding increase in hunting strategies in the areas influenced by the Pitted Ware Culture [1,65,66].

Although both Pitted Ware and Corded Ware practices also affected Eastern Denmark and Scania, the continued construction of palisade enclosures and the (re)use of old megalithic tombs for burials reflect a partial continuation of the Funnel Beaker traditions in these areas, in what is called the Store Valby phase (c. 3000–2600 BCE, see Fig 1, A.1) [1,67,68]. However, the disappearance of larger sites with extensive cultural layers, the regeneration of the forest, and the prevalence of tanged arrowheads have been attributed to a partial shift from agriculture to foraging strategies on Zealand at the end of the MNB [1].

A supra-regional trait characterising the MNB is the scarcity of identified house remains. This could indicate an overall more mobile lifestyle with fewer permanent dwellings than in the adjacent periods, where house remains are more abundant [69].

#### The Late Neolithic

The following period, the Late Neolithic (LN, 2350–1700 BCE), is best known for the reintroduction of metal, copper alloys in particular, in Southern Scandinavia [70] and for the sudden appearance of elaborate bifacial flint tools and weapons over large areas [71,72]. The outstanding bifacial daggers are central objects in studies on the LN [e.g. 71–75] and have even given the period its name (dolktid = dagger period). The distribution of the bifacial flint work across the region is why the LN in Southern Scandinavia is generally regarded as relatively culturally homogeneous [1,76]. However, some of the regional cultural differences of the MNB persisted into the first half of the LN: While the Western European Bell Beaker phenomenon influenced Jutland, the Southwestern part of Norway and West Sweden in the first half of the LN most clearly reflected by the occurrence of Bell Beaker pottery, so-called archery burials, wrist guards, and characteristic barbed and tanged arrowheads, inhumations in the old burial mounds of the Single Grave Culture continued, while also new mounds were constructed [see 3 for further references]. Still, this left the Danish Islands relatively untouched, and ancient Funnel Beaker traditions partly continued. However, East Denmark came under influence from the northern part of the Únětice area through the metal import in the second half of the LN [3,77–79]. However, connections to the Swedish peninsula, generally less recognised, were also influential. This is most evident from the introduction of new sickle types, houses of Fosie-type (Fig 4) and gallery graves in East Denmark (Fig 5), all originating from the Swedish peninsula [36,37,80]. Migration from Southern Sweden to Denmark from around 2000 BCE has recently been suggested by studies on LN haplogroups [49]. Although strontium studies have not yet demonstrated this development, the overall picture in the LN is that of increased mobility [81–83], supporting the impression of population movements within the period.

**Fig 3:**
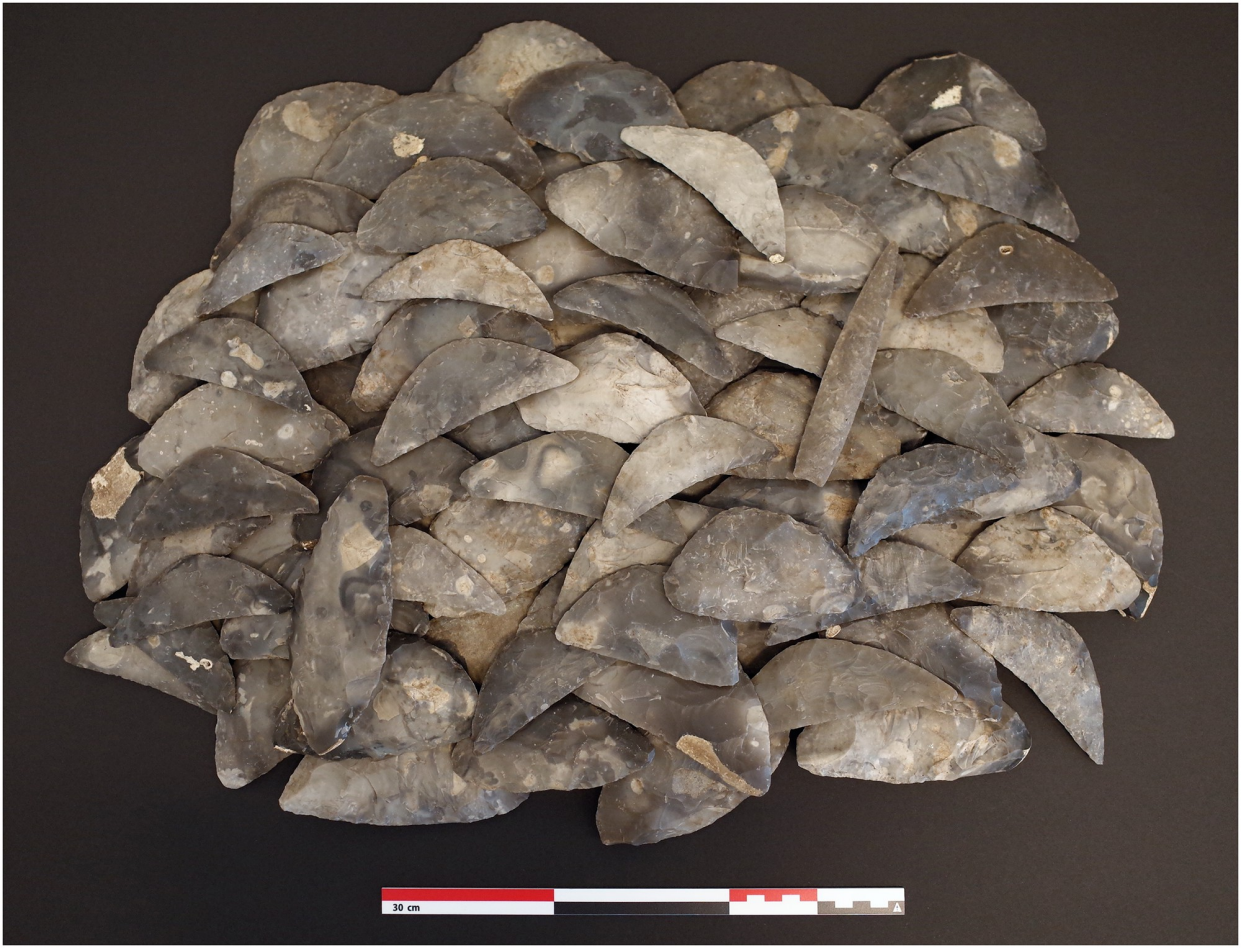
Example of Late Neolithic hoard with sickles. The hoard was found in a wet area in Gillbjerghoved in the most northern part of Zealand, Denmark. In addition to a dagger preform and a spearhead, the hoard included 26 sickles and 86 sickle preforms. The hoard is typologically dated to the LNI (2350–1950 BCE) by the shape of the dagger preform (Type Ix). With its 114 artefacts and 16 kilos of flint, the find represents Scandinavia’s largest hoard of bifacial tools. It is one of at least 268 hoards of crescent-shaped sickles from northern Europe [36]. Photo: Jens Winther Johannsen.

**Fig 4:**
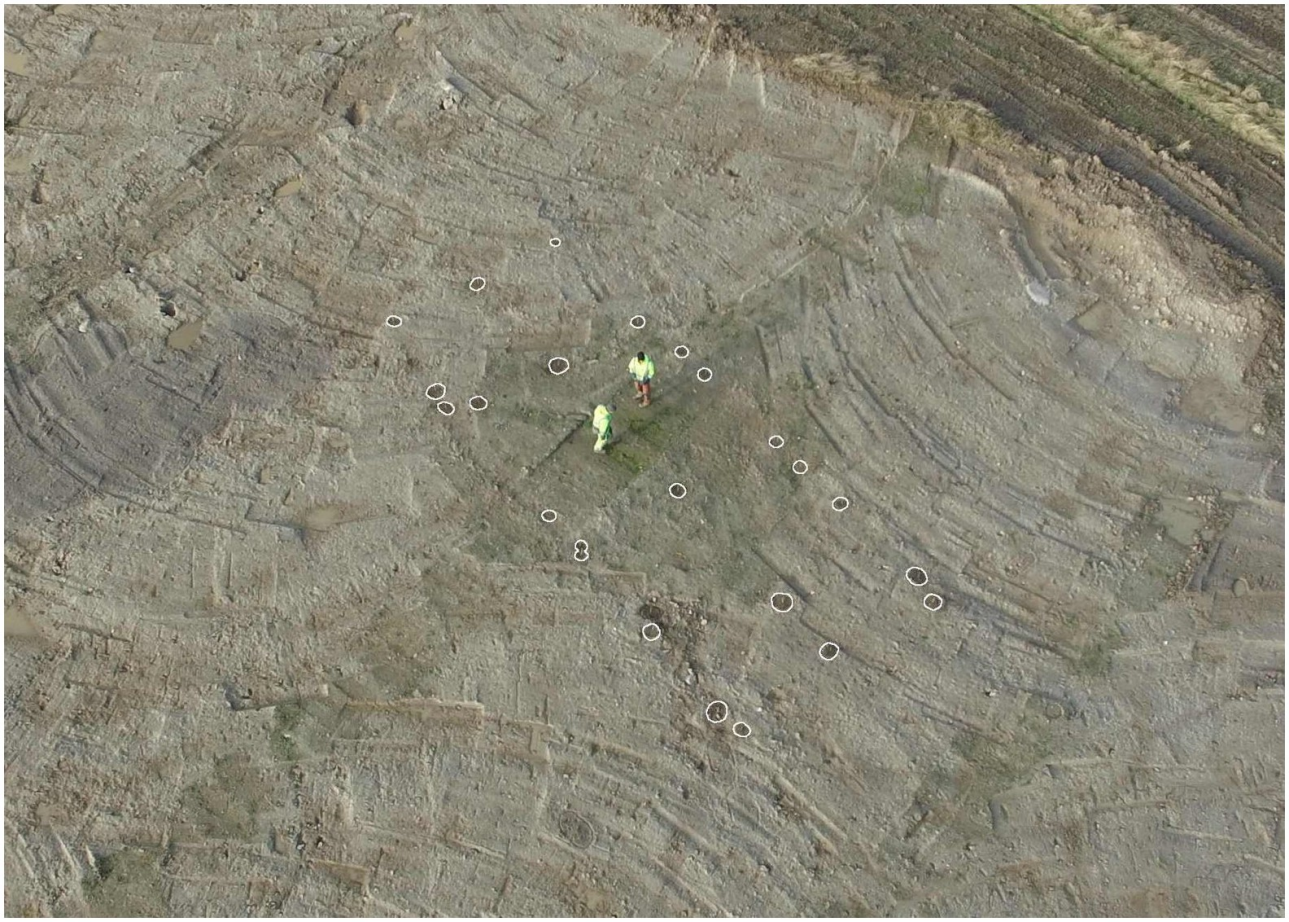
Example of a Late Neolithic two-aisled house. The house, which is of the so-called Fosie type and dated to the second half of the Late Neolithic, was excavated in Vinge in the northern part of Zealand, Denmark [37]. Photo: Martin Hamberg/ROMU.

As mentioned, the period was also marked by a strong revival of the agricultural economy. The most tangible evidence of this is the thousands of bifacial crescent-shaped sickles found throughout the study area (Fig 3). These succeeded the preceding periods’ much rarer harvest knives as the prime reaping tool. Use-wear studies show that the crescent-shaped sickles were mainly used for harvesting cereals, which, together with their large quantities, reflect the importance of cereal cultivation in the LN and the Early Bronze Age (EBA, 1700–1100 BCE) subsistence strategies [36,84]. Agricultural intensification is also reflected in the cultivation of a wider variety of crops than in the preceding period. Spelt, as a new, more cold-resistant crop, was introduced. Culturing a wider variety of crops on the settlement than in the preceding period [37,51,83–86] may reflect diversification as a risk management strategy [85]. Furthermore, a higher ratio of hulled vs. free-threshing crops and the appearing annual vs. perennial weeds indicate a more intensive agricultural practice during the LN [35], which increased the labour input per unit area but also the output per unit area and reduced the risk of yield failure [86,87]. Although nitrogen isotope studies made on carbonised grains have indicated local decreases in the level of fertilisation [88], other archaeological evidence point towards intensification: Analyses on charred cereal remains, the regular relocation of houses, and the ploughing of former house plots indicate that crop rotation, fallow periods and fertilisation, were integral parts of the LN agricultural practice [37,89–92]. The sudden reappearance of solid houses (Fig 4) within large settlements inhabited for hundreds of years in much of Southern Scandinavia is evidence of increased sedentism [37,90,93–96]. This stability of settlements may be related to an increased reliance on cereal cultivation and demonstrates the ability of the LN farmers to remain in the same area for several generations without over-exploiting soil fertility and other resources [37]. Furthermore, an increase in house size and large finds of carbonised grains in sunken floors show that houses now included crop storage facilities [24,37,69,90,91,95,96], underlining the importance of cereal cultivation to the LN economy.

The heavy reliance on cereal cultivation during the LN is further supported by studies on human material from the period. Recent analyses of collagen-apatite spacing in LN human bone material from Southwest Sweden indicate a notable consumption of plant-based foods [97], while stable isotope analyses indicate a low intake of marine food sources [98,99]. The commonness of dental caries in LN individuals from Southern Sweden supports these findings, as carbohydrate intake, including cereal-based products, correlates with the incidence of dental caries [100,101].

### Material, methods and source criticism

According to the surface geology and the cultural differentiation described above, we have defined four major cultural-geographical regions, which we will compare throughout the present study regarding human population. Fig 1 A.2 shows the chosen regional division into Eastern Jutland (EJ, more specifically North and East Jutland), Western Jutland (WJ, more specifically Southwest Jutland), the Danish Islands (DI) and Scania, including the island of Bornholm (SB).

In the present study we, as mentioned, use a quantitative approach based on radiocarbon dates, archaeological objects and palynological analyses to investigate human population dynamics, or the intensity of human activity, during the MNB and LN in Southern Scandinavia. It is important to note that we are dealing with proxies of relative measures, which cannot be translated into absolute population numbers. This also means that the level of population density can vary considerably between the regions. However, the internal dynamics can be correlated, compared and interpreted.

All our proxies have potential biases and shortcomings regarding their use as simple demographic proxies, which is further described below. Using a multi-proxy approach is a deliberate choice, as a lack of synchronicity can be considered a useful tool for identifying potential biases in the individual proxies being used in the research [26].

### Radiocarbon data

To present the demographic development of our study area, we use the established ’dates as data’ approach. The basic assumption is that the longer and more intense an occupation is, the more datable material will be produced and that radiocarbon dates thereby reflect population density [102]. In the last two decades in particular, this approach has undergone rigorous methodological development and has become increasingly important in archaeodemographic research, which involves using summed probability densities (SPD) and related methods of radiocarbon dates from archaeological records [25,103,104].

For the present study, we obtained radiocarbon dates from a variety of sources, including the Xronos database [105], the Feeser et al. [26], Blank [106] and Friman and Lagerås [40] datasets. In addition, our data were supplemented by the compiled dataset of Bunbury et al. [24] and our own data collection (see D01 in S1 Supporting information). We used the R package ’rcarbon’ [104] to calculate temporal models from the radiocarbon dates (see C01 in S1 Supporting information).

The use of SPDs and related methods has however been criticised for the difficulty of distinguishing demographic signals from statistical noise. Therefore, we used composite kernel density estimation (KDE) models to approach population dynamics, which provide a more robust alternative to summed probability distributions and address issues such as sampling error, chronological uncertainty and calibration artefacts. The KDE approach implies randomly selecting a subset of the sample dataset, calibrating the dates, sampling a calendar date from the calibrated distributions, and performing a univariate kernel density estimation. This process is repeated to obtain an ensemble of KDEs from which measures of uncertainty can be visualised as an uncertainty envelope [28,107,108]. A kernel with a bandwidth of 50 years was used to compute the KDE models, and 1000 simulations were performed.

We excluded radiocarbon dates with high measurement errors (>100 ^14^C years) and restricted them to dates falling within our defined time frame of 2850–1700 cal. BCE, +/- 100 years, to avoid boundary effects. We also excluded radiocarbon dates from marine environments, in our case, dates from shells, to *prevent* the influence of the reservoir effect [109]. Dates from dated materials such as food remains (n dates = 30; < 1.5% of dates) and human bones (n dates = 273; < 13.3% of dates), which may be influenced by marine diet, were not excluded, as we expect a general low marine diet, which as mentioned is supported by studies on stable isotope analyses of Late Neolithic human bone material from Southern Sweden [98,99]. However, a potential skewing of calibrated ^14^C ages through marine diet for human and other bone material cannot entirely be ruled out. The refined dataset includes 2060 radiocarbon dates from 668 sites, which we use to generate KDE models as proxies for population development across the study area and the *described* regions. The radiocarbon dates are modelled using the IntCal20 curve [110]. Annual growth rates are calculated from the KDE models (see C04 in S1 Supporting information), which may reflect the pace of demographic change leading to so-called ’boom’ and ’bust’ events in the SPD and KDE models [28].

The radiocarbon dataset may be subject to bias in the initial data collection due to research history or taphonomic circumstances [25,111,112]. Regarding the present study, the rates of radiocarbon dating may have an underlying bias due to archaeological research practices. On Danish excavations, it is common practice to obtain three radiocarbon dates from each house structure where possible, whereas sampling practices for other features are less systematic. Consequently, most of the 2060 radiocarbon dates used in this study are from houses, respectively, settlements (c. 73% of the dataset). The circumstance that house remains are much more abundant in the LN than in the MNB may very well be related to an increase in population during the LN, but could also be related to architectural changes from the MNB to the LN: As mentioned above, sedentism seems to have increased during the LN, leading to the construction of more permanent, robust house types that are easier to identify than less permanent dwellings associated with the more mobile lifestyle that may have prevailed in the MNB [31]. To avoid strong bias due to sampling practices, we have endeavoured to include all available radiocarbon dates from human-made structures. Nevertheless, the described sampling strategy is a potential bias in our study. Here, it is also relevant to note that the map of the geographical distribution of sites and the number of radiocarbon dates (Fig 1B) reflects the clustering of dates around urban areas and infrastructure, while other dense concentrations may represent archaeological focus regions and local research interests [35,90,113], which also represents a bias in our data.

Fig 1B shows the spatial distribution of radiocarbon dates in our study area within the defined time frame. The distribution of dates, sites and mean dates per site within the boundaries of our proposed regions are shown in Table 1. Bunbury et al. [114] suggested using at least 200 ^14^C dates over spans of 750 years, while Williams [115] suggested 200–500 dates for 14,000 years, respectively, to construct robust SPDs. Our regions meet this requirement (Table 1), except for WJ, which has significantly fewer radiocarbon dates and archaeological sites than the other regions. Although the number of dates is below the suggested threshold, we would argue that because of the use of the KDE approach, the KDE model from radiocarbon dates can also be used to make cautious statements about the population dynamics of WJ.

**Table 1:** Count distribution of dates, sites, and mean dates per site by site type.

	N dates			N sites			Mean dates per site		
	All	S	B	All	S	B	All	S	B
Meta region	2060	1496	377	668	453	150	3.08	3.08	2.95
Scania and Bornholm (SB)	918	608	205	321	217	73	2.86	2.5	3.6
Danish Isles (DI)	368	279	44	114	78	23	3.18	3.5	1.9
Eastern Jutland (EJ)	698	547	107	200	147	39	3.52	3.65	2.97
Western Jutland (WJ)	76	35	21	33	11	15	2.3	4.1	1.4

All = all site types; S = settlements; B = burials.

### Graves including Middle Neolithic B battle axes or Late Neolithic daggers

While architectural differences between MNB and LN may bias differences in radiocarbon dates between the MNB and LN, graves from both periods are well represented. Danish Neolithic burials have been an important source material since the dawn of archaeology [116–118], and their publication in detailed catalogues provides a thorough overview of the burial landscape [51,72]. Because of the long research history, the MNB/LN graves are less affected by the described biases derived from the intensity of modern development work, etc., than radiocarbon dates are.

**Fig 5:**
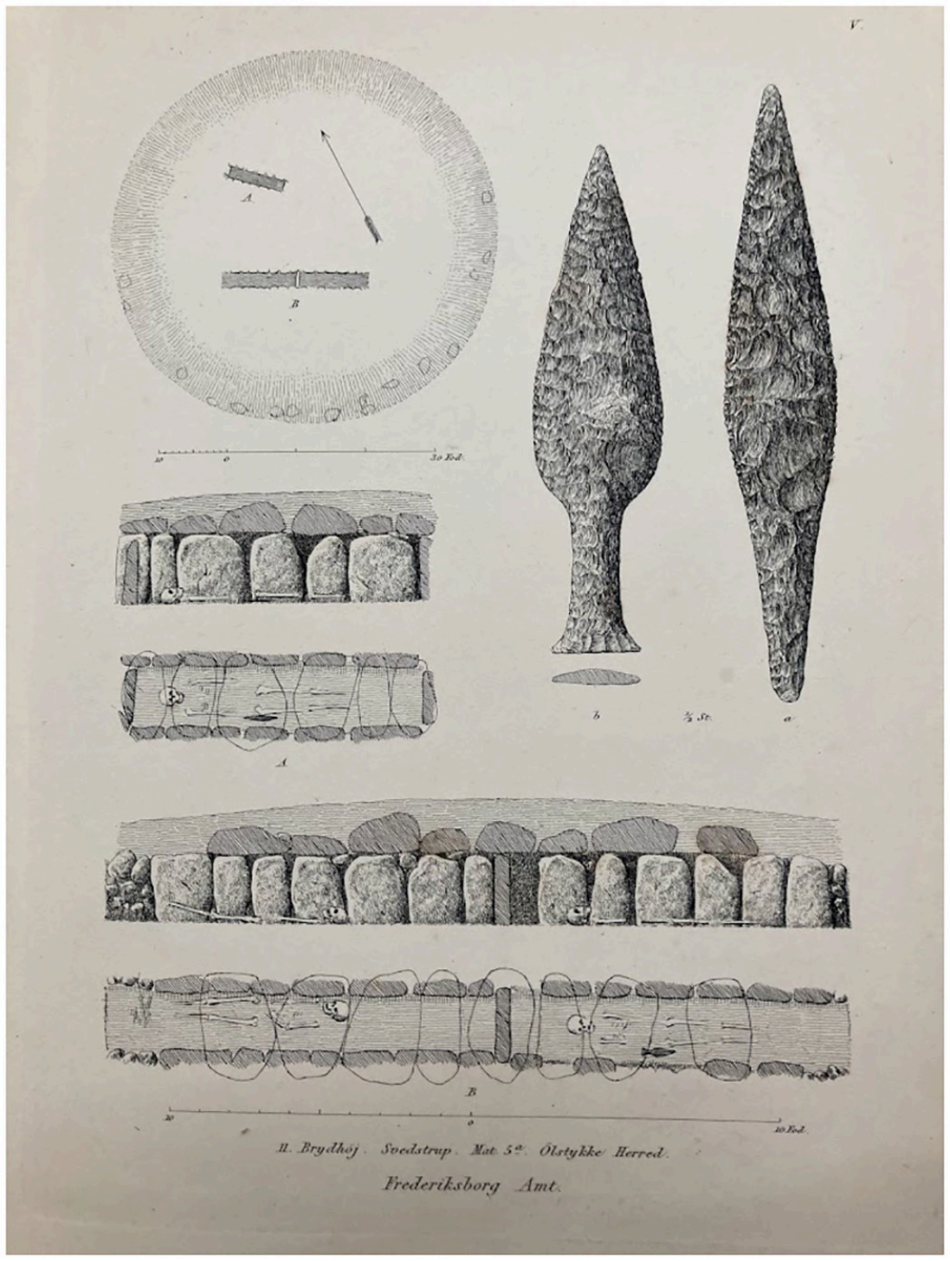
Example of a Late Neolithic grave. The print shows the burial mound Brydhøj, which was found between Svestrup and Ølstykke in Northern Zealand, Denmark. The mound contained a double and a single gallery grave. The dagger types (ID and VB) show that people were buried here in both the LNI (2350–1950 BCE) and LNII (1950–1700 BCE) (after [120], plate V).

Battle axes were common grave goods during the MNB (Fig 3) and were succeeded by bifacial flint daggers in the LN (Fig 5). Both battle axes and flint daggers found in graves are identified as personal items representing mainly male individuals [51,72]. As burial practices did not change significantly between MNB and LNI [1], and individuals were rarely buried with more than one battle axe or dagger [72,119], comparing their deposition in graves is reasonable. Theoretically, the rates of battle axes and flint daggers in graves can therefore be seen as an expression of the male population over time. Because the two weapon types are also relatively well-dated typologically, graves with battle axes and flint daggers were chosen as a second population proxy.

MNB battle axes have mainly been found in graves [51,121], and the majority of stray finds of battle axes therefore most likely represent destroyed graves. However, stray finds of flint daggers cannot per se be interpreted in the same way as representing destroyed graves, as flint daggers are also commonly found in settlements and hoards [72,122]. To be able to compare the two categories of finds, only battle axes and daggers found in graves were included in the study. We also assume that graves with battle axes and daggers have suffered the same destruction over time. The battle axes were mainly collected from catalogues from Hübner [51] and Schultrich [123], while the flint daggers were primarily collected from Lomborg [72] and Kühn [124]. The difference of 32 years of excavation between the two major studies by Lomborg and Hübner carries the risk of over-representation of battle axes compared to flint daggers. Therefore, graves containing flint daggers excavated after 1973, when Lomborg’s study was published, have also been included (see D02 in S1 Supporting information). Fig 1 C.1 and C.2 show the spatial distribution of battle axe and flint dagger burials in our study area. The battle axe dataset contains 2524 graves, while the flint dagger dataset contains 3280 graves. We use the aoristic dating method to bring the compiled battle axe and flint dagger data into a time series representation [125–127]. This is designed to overcome the limitations of typochronological dating methods. Aoristic dating estimates a probability density distribution on the temporal scale for an event within a defined time frame (start and end date) in chosen time steps (e.g. years). In our case the appearance of the artefact, axe or dagger, is considered to have a uniform probability to date in one year of the given dating span of the artefact. These artefact-dating probability distributions can be summed to produce an aoristic sum time series, which can be compared to an SPD or KDE model based on radiocarbon dates. To compute our aoristic sums, we used the R package ’aoristAAR’ [128]; see C02 in S1 Supporting information).

There are however several significant biases in using the proxy. Graves containing battle axes or flint daggers represent only a fraction of the population: Female burials are unlikely to be represented within the material assemblages, and not all male burials contain a battle axe or flint dagger. Calculations show that likely only 10–20% of the population were buried with a flint dagger [80,129,130]. Furthermore, the percentage of the male population buried with a battle axe seems to have fluctuated over time. While in the early MNB (c. 2850–2600 BCE) almost all male burials included a battle axe, in the later part (c. 2600–2250 BCE), flint axes and blades partially replaced their role. This may be why graves from the second half of the MNB are underrepresented compared to graves from the early part of the MNB [51,91]. The decrease in the proportion of graves with battle axes at the end of the MNB is therefore not necessarily a symptom of a population decline. The use of daggers as burial gifts seems to have declined similarly to battle axes [72], which again is not necessarily a symptom of population decline. Furthermore, graves with MNB battle axes are distributed in Jutland mainly, whereas graves with daggers have a more even geographical distribution within our study area, except for in Southwest Jutland. Finally, regarding Scania, the battle axe/dagger ratios have not been calculated due to the lack of compiling Scanian MNB and LN burials catalogues.

The discussion above shows that graves with battle axes and flint daggers represent only a minority of the population and that their use as grave goods fluctuated over time. But while the battle axe/dagger ratio thus may be of limited value as a proxy for overall supra-regional population development, it is a powerful tool for examining differences in population development between the regions and to contrast the SPD and KDE models.

### Pollen records

Kneisel et al. [131] point out that it is essential to include data independent from the archaeological record in population studies to test for biases in the archaeological material used. A proxy for vegetation openness can indicate human impact, from which demographic trends can be inferred, assuming that increasing population density results in greater forest clearance to meet resource demands, including timber, agricultural land, and settlement area. However, vegetation openness has to be considered an unknown function of population size, subsistence strategy and environmental influences [26,35,132,133]. The benefit of computing the vegetation openness and comparing it against other proxies is to find possible relations that lead to a better understanding of the coupling of population size and land cover change.

Therefore, in addition to radiocarbon and battle axe/dagger data, we use a proxy for human impact derived from pollen records. Using multivariate ordination techniques, such as principal component analysis (PCA), a selection of terrestrial pollen taxa from pollen records can be approached in terms of their indication of vegetation openness. Using this approach, it is expected that the data will separate into forest taxa and more light-demanding taxa on the first axis of the PCA. The latter indicates anthropogenic deforestation and vegetation opening in general [134,135]. As suggested by Feeser et al. [26], we use the time window of 4800–700 BCE to calculate the openness score for our analysis, as the relationships of the represented taxa are most consistent for this time frame. Each pollen sample used in the PCA is assigned an absolute date based on the age-depth models of the pollen records, allowing us to plot the openness score (first PCA axis) as a time series reflecting human-induced land opening. From the interpolated time series, a mean, median, etc. can be calculated, which we argue characterises a regional trend in vegetation openness [133]. We choose a temporal resolution of 50 years for the representation of vegetation openness in Northern Jutland and Zealand due to the temporal resolution of the average pollen record. See C03 in S1 Supporting information for more details on the calculations of the vegetation openness score.

A drawback of this method is that the proxy must be treated with caution as to which geographical region’s trajectory of vegetation development it represents. A combined analysis of several nearby pollen records may obscure local trends and dynamics or overestimate individual records relative to others. However, we would argue that a temporally stable regional vegetation openness score results either from antagonistic behaviour in vegetation openness at the sites of the different records or similar openness scores of the individual records used. The first case may represent a spatial shift of settlement activity and the second a stable state of condition of settlement activity over time. An increase or decrease in the regional vegetation openness score may of course result from parallel developments in all pollen records or from ’extreme’ values in single or a few pollen records. We would argue that the intensity of vegetation openness is also meaningful in this case and that an increase/decrease in regional vegetation openness is related to population/human impact.

Unfortunately, the available pollen records cannot provide reliable openness values for the defined geomorphologically/archaeologically distinct regions. However, we can calculate vegetation openness for the northern half of Jutland (NJ) and Zealand and use the well-published Lake Belau vegetation openness record [26,136] for the southern half of Jutland (SJ) to visualise human-induced land opening. Fig 1D shows the distribution of the pollen records used in our study area and the corresponding suggested reference regions. A vegetation openness score was not calculated for Scania, as the openness gradient was not clearly projected onto the PCA’s first or second principal competent (see C03 in S1 Supporting information). The data set for NJ consists of the off-site pollen records from Store Økssø, Navnsø [137], Ovesø [138], Skånsø, Kragsø and Solsø [41]. The off-site pollen records from Dalby Sø [unpublished, but see 33] and Korneup Sø [139] were used for Zealand. In addition, the on-site pollen record from Vinge [37,140] is added for observation, but due to the lack of beech (*fagus*) pollen in the samples, integration into the regional PCA would obscure the openness gradient and the dataset was therefore omitted.

### Time series, correlation and events

With the proxies described above, we have several time series that can be examined for correlations and events of population dynamics in Southern Scandinavia. We acknowledge that these time series have intrinsic temporal uncertainties, and the correlation analysis presented here can only be an additional measure to approach our questions about temporal relationships.

To perform any correlation test or event detection, we use detrended versions of the time series or growth rates (C05 in S1 Supporting information). Detrending a time series involves removing the underlying trend component to focus on the remaining variation. It can help to reveal cyclical patterns, i.e. oscillations or repetitive movements that are not directly related to the overall trend. The growth rate of a time series measures the percentage change in the value of the series over a given period. It helps assess the rate at which the series increases or decreases over time. By examining the growth rate, it is possible to identify more short-term trends. Furthermore, when comparing different time series, looking at the growth rates allows a standardised comparison [141]. Neither the detrended time series nor the calculated growth rates are stationary. When a time series is said to be stationary, its statistical properties (e.g. mean, variance) remain constant, and its autocorrelation structure does not change over time.

To test for relationships within our regional setup and between our different proxies, we test for correlation between them with a pairwise Pearson correlation [see 139] of the total time window of observation (2850–1700 BCE) and the respective time windows of MNB (2850–2350 BCE) and LN (2350–1700 BCE). Although we represent all our population proxies as continuous time series, we only use the KDE models in this way for calculating correlations. For the correlation test of the other proxies, the data were binned into blocks of 25 or 50 years. We applied cross-correlations to identify possible time-lagged relationships between our selected regional demographic trends and the proxies. This method can identify temporal leads and lags between two variables and the strength of the relationship between them. We also checked the demographic proxies for autocorrelation, which is the degree of similarity between a variable and a lagged version of itself over successive time intervals [141,142]. Depending on the temporal resolution of the proxy, we used a non-binned or binned version of the proxies, similar to simple correlation. When using any of the above correlation techniques, one must be careful of spurious correlations. Spurious correlation occurs when there seems to be a connection between two variables, but in reality, this connection is purely coincidental and lacks any meaningful relationship. In simpler terms, when two things appear to be related, but they are actually not connected in any significant way [141].

We use the event detection method when looking for ’significant’ changes or anomalies in our time series. This involves examining the data for sudden changes in trends, patterns or behaviour that may indicate a significant event or phenomenon. We use the common threshold-based approach to identify outliers or abnormal data points (events) using statistical measures. An event is detected by exceeding or falling below a given threshold (e.g. standard deviation, percentile) calculated from the time series [28,143]. We use the standard deviation because we often deal with smoothed data (e.g. KDE) where the variance is less pronounced (see C06 in S1 Supporting information).

## Results

The results section is divided into three sub-sections that look at the evolution of our proxies over time at different geographical scales. The procedure for preparing the data and plotting the results can be found in the C04 and C05 in S1 Supporting information.

### KDE models

We carried out preliminary data handling and presentation tests [28,139]. The normalisation of the radiocarbon dates does not alter the general trend in the SPD and shows a high correlation (pearson-r: 0.83) with the non-normalised SPD. The comparison of an SPD including all radiocarbon dates and one using only dates from short-lived materials also shows a strong correlation (pearson-r: 0.9), providing confidence that the inclusion of radiocarbon dates from non-short-lived materials will not obscure developments in the following demographic models.

**Fig 6:**
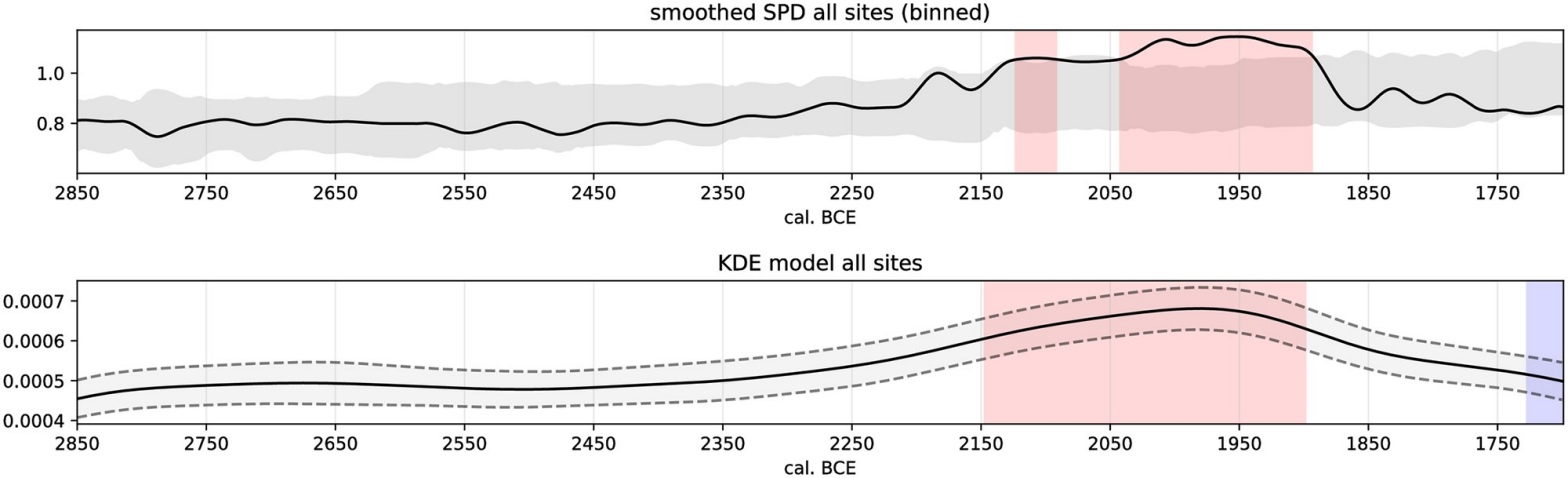
Top: Summed probability distribution of all radiocarbon dates (black solid line). Plotted against an exponential model (grey envelope). Bottom: KDE model of all radiocarbon dates (black solid line) with given uncertainty (grey envelope). Deviating positive (red highlights exceptional human activity) and negative (blue highlights exceptional human activity) events indicated.

Population density based on radiocarbon dates only (Fig 6) shows a stable development from 2850–2400 BCE, which implies little change in the large-scale demographic trajectory of the MNB in Southern Scandinavia. From about 2400 BCE, we see an increase in population, culminating in a boom between 2150 and 1900 BCE. The boom phase was determined by comparing our SPD model with a theoretical exponential growth model [see 104] and by our event detection of the KDE model. After 1900 BCE, a sudden decline in human activity indicates a decreasing population. Based on this observation, we can identify a major event within our time frame that corresponds to the cultural phenomenon of the LN in our research area.

**Fig 7:**
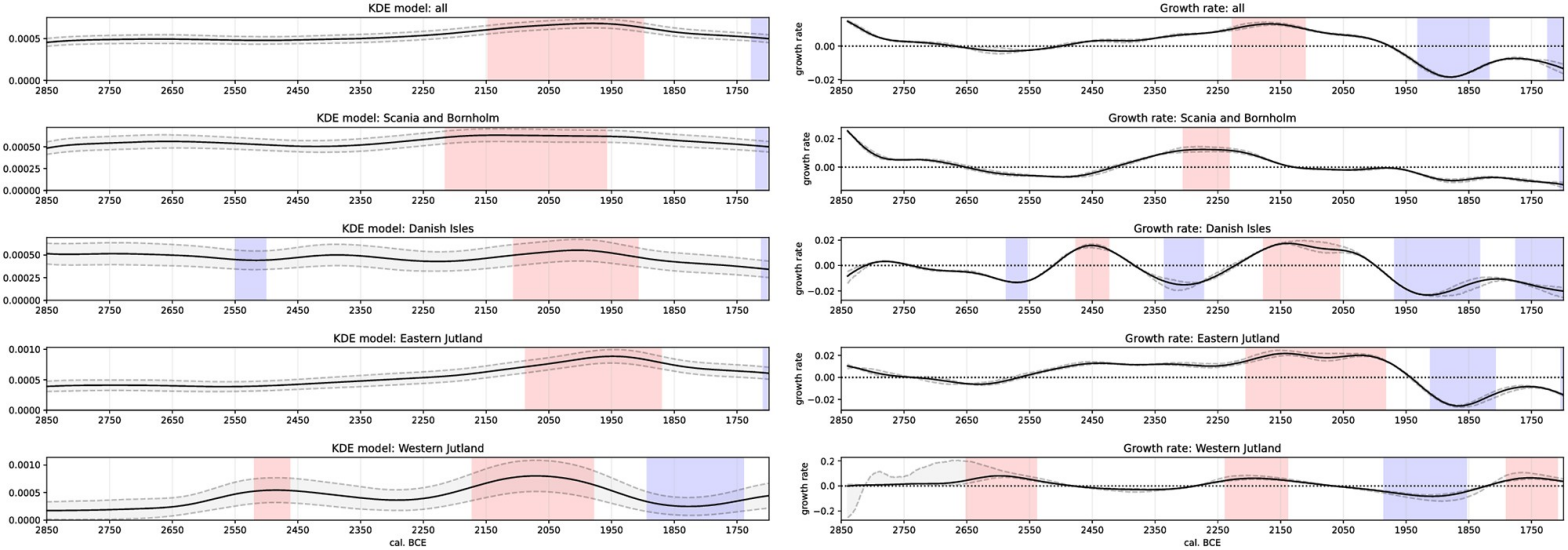
Left: KDE model of all radiocarbon dates in the respective region (black solid line) with given uncertainty (grey envelope). Deviating positive (red highlighting, population density) and negative (blue highlighting, population density) events indicated. Right: Growth rate of the KDE model in the respective region (black solid line) with given uncertainty (grey envelope). Anomalous positive (red highlighting, population density) and negative (blue highlighting, population density) events indicated.

Examining the regional KDE models (Fig 7), the over-regional trend of stable conditions for the MNB is not present in Western Jutland (WJ) and the Danish Islands (DI). WJ shows increased densities around 2600 BCE, with a significant positive event around 2500 BCE and a non-significant but discernible decrease for the onset of LN (2350–2200 BCE). This low is followed by a significant peak of human activity around 2150–2000 BCE in the WJ, followed by a decline. Here, it must be remembered that the results from WJ must be interpreted with some caution due to the small amount of radiocarbon dates from the area. While the growth rate decreases after 2500 BCE in WJ, in Eastern Jutland (EJ) and on the DI, growth rates increase, resulting in higher population densities. The KDE model of the DI shows a population decline after 2550 BCE. A first, although not significant, density peak can be observed at the beginning of the LN, around 2400–2350 BCE. This is followed by a dip in development, accompanied by significantly low growth rates. Around 2200 BCE, the population grows again and is particularly high around 2100–1900 BCE. The early peak around 2350 BCE on the DI is mainly the result of dates from settlement contexts correlating to a plateau in the radiocarbon calibration curve around 2450–2200 BCE [144]. Therefore, the abrupt increase in settlement activities on the DI should be treated with caution, as many of the dates have a larger dating uncertainty and show a higher probability of dating 100–200 years later than 2350 BCE (see D01 in S1 Supporting information). Negative growth rates are present between 2375 and 2200 BCE, resulting in a population decrease between 2275 and 2175 BCE. This is followed by a significant increase in population from 2175–2000 BCE, while a decline on the DI can be observed from 1950 BCE onwards. The density of the EJ shows a constant increase, also represented by positive growth rates, from about 2500 to 2000 BCE, with a peak from 2100 to 1875 BCE. The decline in population density after this peak is pronounced. It is noticeable that the growth rates of EJ and the DI both increase around 2550 BCE, but EJ does not show a population decline until 1875 BCE. This shows that although we do not have significant events in the KDE model, the growth rates identify changes in population dynamics. The region of Scania/Bornholm (SB), on the other hand, can be described as smooth and shows a slightly later appearance of positive growth rates compared to the DI and EJ, dating back to the transition from MNB and LN around 2400 BCE. A smooth decline follows the significant peak in population density between 2200–1975 BCE. The population increase of 2200–1975 BCE in SB precedes those of the DI and EJ. Regarding growth rates, SB shows slightly antagonistic behaviour to the DI and EJ. While the positive growth phase of SB from 2400–2150 BCE precedes that of the DI, the DI shows a significant growth decline between 2350–2250 BCE.

Interestingly, the significant positive growth event of EJ from 2200–1900 BCE overlaps with the late positive growth of SB. In summary, both SB and EJ show less variation in population density during the MNB, with significant events occurring during the LN. For all regions except WJ, there are significant positive deviations in the LN with some time offset.

According to the correlation test of our time windows (Tables 2–4), the detrended KDE models of all regions and all derived growth rates show a positive relationship during the LN (Table 4) and less strong over the whole observation period (Table 2). The correlation between the DI and EJ is very strong in all time windows compared to other correlation pairs. This suggests a closer relationship between these two neighbouring regions, while other neighbouring regions show fewer similarities. Especially during the MNB, most correlation pairs show negative coefficients or almost no correlation (Table 3), except for the DI and EJ. Together with the higher densities of the KDE model, this argues for a higher human activity in the western part of our study area compared to others. But also SB shows initially higher growth rates at the beginning of the MNB and population densities during the MNB, while EJ and the DI show very little change.

**Table 2:** Pearson correlation of the regions’ *detrended KDE models* | KDE model growth rates in the period of 2850–1700 BCE (MNB-LN).

	SB	DI	EJ	WJ
SB	1			
DI	*0*.*56* | 0.21	1		
EJ	*0*.*68* | 0.53	*0*.*86* | 0.74	1	
WJ	*0*.*5* | 0.032	*0*.*49* | 0.18	*0*.*27* | 0.085	1

**Table 3:** Pearson correlation of the regions’ *detrended KDE models* | KDE model growth rates in the period of 2850–2350 BCE (MNB).

	SB	DI	EJ	WJ
SB	1			
DI	*-0*.*18* | -0.14	1		
EJ	*-0*.*38* | 0.19	*0*.*91* | 0.68	1	
WJ	*-0*.*33* | -0.38	*-0*.*82* | -0.69	*-0*.*67* | -0.81	1

**Table 4:** Pearson correlation of the regions’ *detrended KDE models* | KDE model growth rates 2350–1700 BCE (LN).

	SB	DI	EJ	WJ
SB	1			
DI	*0*.*69* | 0.32	1		
EJ	*0*.*77* | 0.67	*0*.*92* | 0.77	1	
WJ	*0*.*74* | 0.19	*0*.*69* | 0.4	*0*.*54* | 0.26	1

We ran the autocorrelation and cross-correlation for each of our three time windows and with the detrended KDE models and growth rates. The correlation studies show several things. The autocorrelation of regions shows some implicit cyclicity but not strong enough to draw conclusions. The cross-correlation often shows the strongest correlations without a time lag. EJ and the DI show a strong link in population dynamics (see also Table 4). The cross-correlation suggests that EJ precedes developments on the Danish Islands in growth by a few years to decades. SB and WJ often show lagged (negative) correlations (50–300 years) with EJ and the DI. This may indicate geographical shifts in population density over time and space and possible migration/diffusion/mobility dynamics within our study area and neighbouring regions.

### Aoristic sum

**Fig 8:**
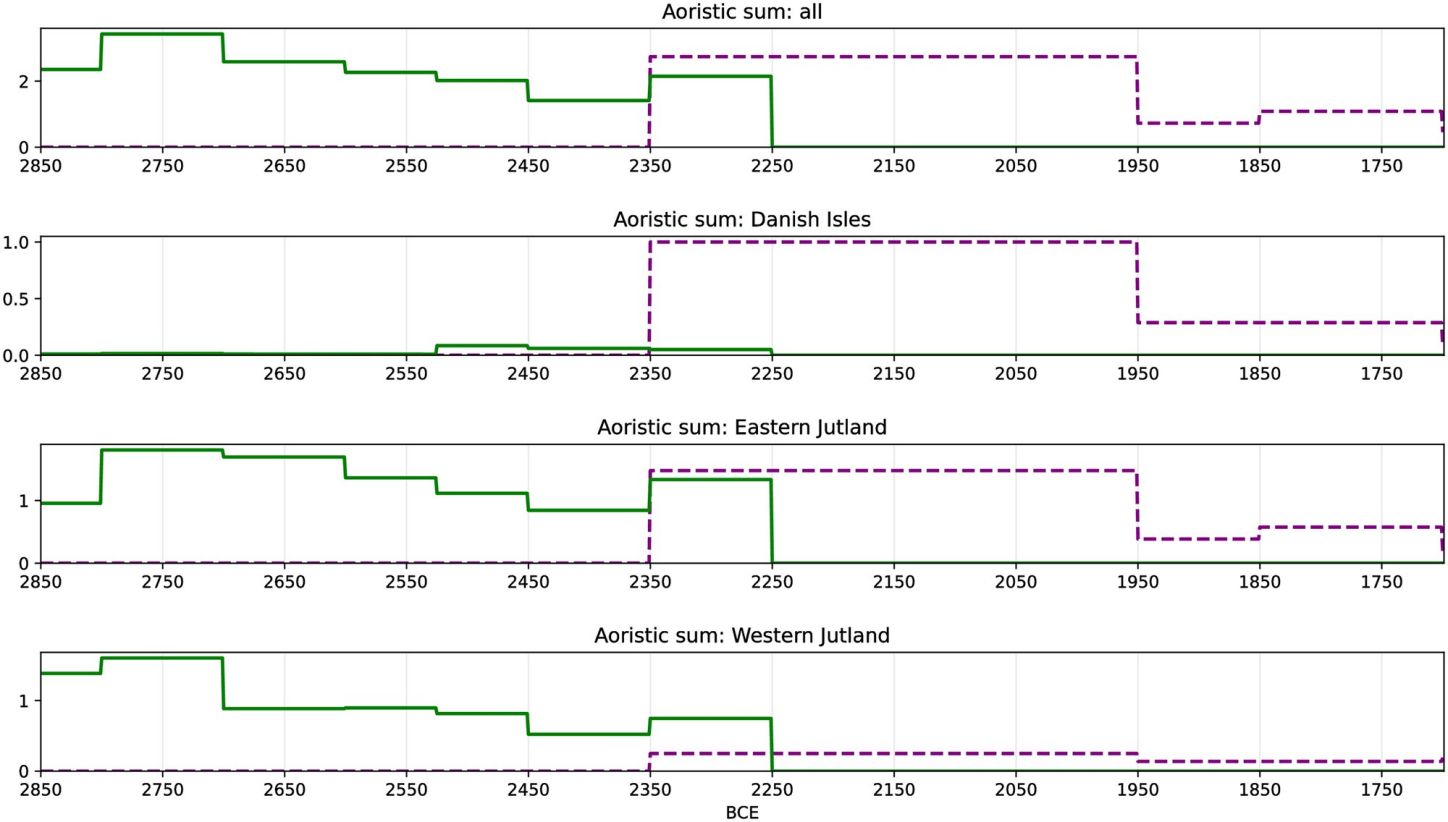
Supra-regional and regionally differentiated aoristic time series of axes (green solid line) and daggers (purple dashed line).

The data set of battle axes and flint daggers shows their temporal distribution throughout the study area and in the defined regions (Fig 8). The clear typochronological division between the two object categories with the small overlap between 2350–2250 BCE [1,51] is well visible. Both aoristic time series show a similar pattern of temporal development: With the beginning of the respective archaeological phase, the number of new objects peaks rapidly and then steadily declines over time. An exception, however, is the axes of type K and L, which date to the latest MNB/earliest LN (2350–2250 BCE [1,51]).

Looking at battle axes and flint daggers from a regional perspective, EJ and WJ show similar trends in MNB material, although the decline in the number of battle axes is more pronounced in WJ than in EJ. The density of MNB axes on the DI is very low. With the beginning of the LN and the appearance of the daggers, we see a rapid increase in these in the EJ and the DI. This increase is not visible in the data from WJ.

A small number of axes or daggers can indicate changes in human activity, but also possible cultural preferences and choices. This is certainly the case for the small number of battle axes on the DI, as the practice of single-grave burials never gained a foothold in this region [58]. Demographic trends and relations to other regions do not suggest a comparably low level of human activity (see section Population: KDE models).

### Vegetation openness scores

**Fig 9:**
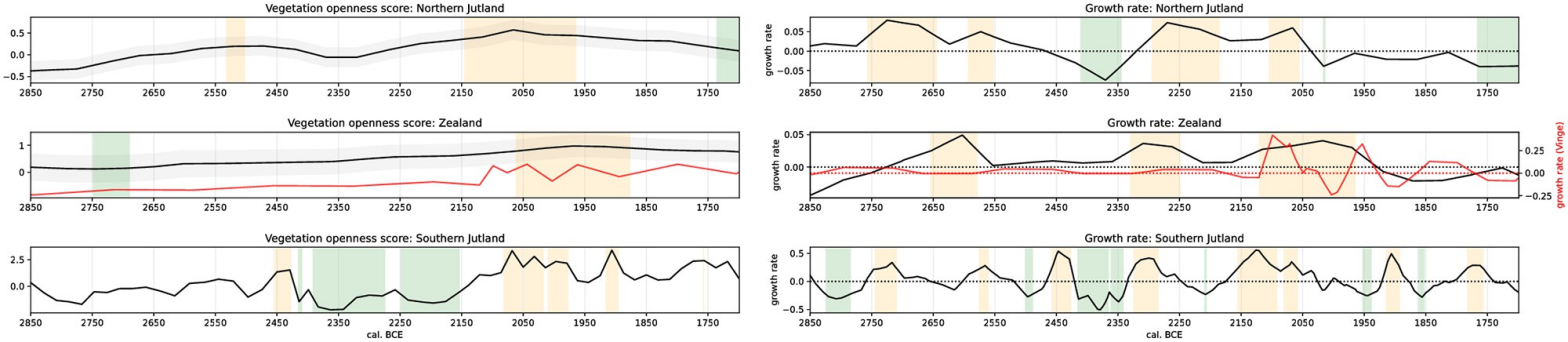
Left: Vegetation openness scores for the palynological records from Northern Jutland, Zealand, the Vinge record (red dotted line), and of Southern Jutland (Lake Belau). Deviating positive (yellow highlighting, exceptional openness) and negative (green highlighting, exceptional closeness) events indicated. Right: Growth rate of vegetation openness scores. Deviating positive (yellow highlighting, increasing openness) and negative (green highlighting, decreasing openness) events indicated.

Fig 9 shows the comparison of the vegetation openness scores and their growth rates of the three different regions: North Jutland (NJ), South Jutland (SJ) and Zealand (cf. Fig 1D). It must be remembered that these regions do not correspond to the regions of our two proxies presented earlier due to shortcomings in the available pollen data discussed above. However, a trend of increasing vegetation openness is visible for all regions in the differentiated pronunciation. It should be noted that the vegetation openness score for NJ and Zealand is an average of all included pollen records, and the result has been smoothed. Calculating vegetation openness for the whole region does not give a conclusive result, as the ecological differences are too large. Treating Jutland as a whole shows a common gradient of openness, but the magnitude is higher in the North due to environmental reasons. Nevertheless, the combined regional analysis of NJ and Zealand and the single record from Vinge show little variation or a significant positive event of vegetation opening during the MNB (2800–2350 BCE). The high-resolution temporal record of Lake Belau, representing SJ, shows a slightly more differentiated development of the MNB, suggesting a trend of vegetation opening from 2800–2450 BCE but including phases of short-term decrease in open land. A decrease in open land and possible reforestation in SJ appears around 2400–2150 BCE. NJ and Zealand show a continuous increase in open vegetation from 2450 BCE to the end of our observation period, with a positive event from c. 2350–2050 BCE. The Zealand records show an increase in vegetation openness from about 2200 BCE, with the highest vegetation openness values between 2050–1900 BCE. From this point on, vegetation openness decreases again but does not reach the low vegetation openness of the MNB. Vinge shows a strong increase in openness from 2100 BCE onwards, which is maintained. Similar to Vinge and less pronounced than Zealand, the Belau record shows rather rapid vegetation opening around 2150 BCE, peaking 2075–2000 BCE. The period after 2000 BCE shows some dynamics of decrease and increase of open area, but the openness remains at a high level. From the point at which Vinge shows signals of human influence, around 2100 BCE, its growth rates are similar to Lake Belau’s, but about 50 years earlier.

These observations suggest that the pollen-based proxies for vegetation openness, similar to the radiocarbon models, show a kind of stability of human impact during the MNB and an increase from the LN onwards. Zealand and SJ show a temporal overlap of a significant positive event from c. 2100–1900 BCE, while NJ shows stagnation in openness during this period, even a very slight decrease. This suggests a change in human impact from MNB to LN. NJ already shows a high human impact during the early LN.

**Fig 10:**
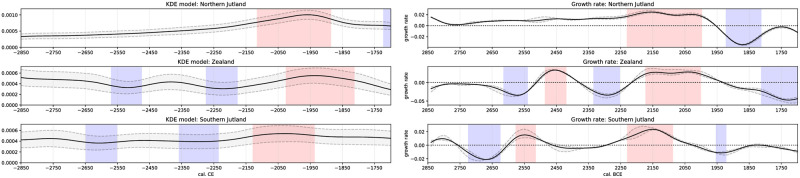
Left: KDE model of all radiocarbon dates in the respective region (black solid line) with given uncertainty (grey envelope). Deviating positive (red highlighting, exceptional population density) and negative (blue highlighting, exceptional population density) events. Right: Growth rate of the KDE model in the respective region (black solid line) with given uncertainty (grey envelope). Anomalous positive (red highlighting, population density) and negative (blue highlighting, population density) events indicated.

To compare vegetation openness with another proxy for population density, we computed further KDE models from the radiocarbon data in the reference regions of the pollen record (see Fig 1D). These KDE models show the expected change in trajectories (Fig 10). Zealand is very similar to the evolution of the DI, with little difference due to missing data from the other islands. NJ and SJ both show a mixture of WJ and EJ trajectories due to the new regional summation of radiocarbon dates (cf. Fig 7). It is clear that NJ, in particular, experienced a steady increase in human activity from the late MNB onwards, culminating in the LN boom around 2100–1900 BCE. The less pronounced start of LN in NJ is obscured by the WJ data, which influences the northern reference region for the openness score. Similarly for SJ, with a stronger bias from the WJ trajectory, the wavy pattern of WJ becomes visible (cf. Fig 7). The SJ seems to be related to developments on Zealand, as two negative deviations around 2650–2550 BCE and 2350–2250 BCE, as well as the positive peak from 2150–1950 BCE, preceded the significant population events on Zealand. Interestingly, the indication of population decline in the KDE models at the end of the LN around is not as strong in the vegetation openness scores, suggesting a bias in our datasets for this particular period.

The general trend is an increase in comparing the vegetation openness of the region (Fig 9 right) and the detrended regional KDE models (Fig 10 right) with their counterparts. The identified positive events in both time series show an overlap. However, in many cases, the timing is not as expected, as the vegetation openness events seem to occur earlier than those in the corresponding KDE model. This may be due to the chosen bandwidth of 50 years for smoothing the KDE models or a possible dating uncertainty of the pollen record’s time depth models. Another possibility for the unexpected lead of landscape opening to the KDE models could be the lagged relationship between population growth and landscape opening. Therefore, it is important also to look at how vegetation openness and KDE model growth (Fig 10 *left*) may relate. Rather than assuming the straightforward relation of increasing population equals increasing human impact, it is likely that human impact, the intensification of agricultural production, precedes population growth. The Vinge, Zealand and NJ datasets show an earlier increase in vegetation openness than the KDE model would suggest for population density. However, population growth precedes the peak of vegetation openness, and in the case of Zealand and Vinge, it even marks the start of meaningful openness growth (Fig 9 *left*). For SJ, our vegetation openness proxy shows increased human impact in the LN, which occurs later than further north but is nearly contemporary with Zealand. The KDE models provide insight of higher population densities at about the same time for NJ and SJ. A period of reforestation in SJ around 2400–2150 BCE and a low population density indicated by the KDE model within this time window coincide in SJ. Growth rates of the KDE models begin to increase around 2250 BCE, while we see peaks in the KDE model around 2140 BCE and vegetation opening from around 2075 BCE. In SJ, the assumed relation of higher population density results in more open land seems to hold. The population decline in the late LN and EBA is visible in all three KDE models and their growth rates. However, the SJ KDE model suggests only a small population decline and remains consistent with the openness score of Lake Belau. The long increase in vegetation openness in NJ from about 2300–2050 BCE is more or less accompanied by a significantly increased growth rate in the corresponding KDE model. At the peak of population density around 2100–1900 BCE, vegetation openness decreased slightly.

The correlation coefficients of the binned versions of the vegetation openness score and the corresponding population time series (Table 5) show little to strong direct linear correlation. The correlation between the detrended KDE model and the vegetation openness scores is generally higher than the growth rates. Zealand and SJ show high correlation coefficients for the whole period and the LN. The cross-correlations between vegetation openness and demographic time series yield inconclusive results. While in NJ and Zealand population growth precedes vegetation openness, in SJ human impact increases before the KDE population models.

**Table 5:** Pearson correlation of the regions’ *detrended KDE models vs*. *vegetation openness score* | KDE model growth rates vs. vegetation openness score in the different periods.

	2850–1700 BC (MNB-LN)	2850–2350 BC (MNB)	2350–1700 BC (LN)
VOS vs. Demography NJ	*0*.*2* | -0.079	*0*.*74* | 0.55	*-0*.*19* | -0.23
VOS vs. Demography Zealand	*0*.*64* | 0.27	*-0*.*58* | -0.26	*0*.*74* | 0.53
VOS vs. Demography SJ	*0*.*51* | 0.23	*-0*.*01* | -0.13	*0*.*77* | 0.44

Overall, the comparison of vegetation openness and the KDE models shows that attempted correlations are not straightforward. Although the positive deviation of both proxies on Zealand and SJ overlap to a large extent, population does not precede vegetation openness. This finding differs from that of Feeser et al. [26] for Southern Jutland and Mecklenburg-Pomerania, Germany. Our KDE models smooth the temporal resolution of the demographic proxy, and visible wiggles in the commonly used SPD curve may coincide with the evolution of the vegetation openness proxy curve. Furthermore, the Danish pollen records lag behind the high temporal resolution of Lake Belau, obscuring possible temporal relationships. This result indicates that changes in the modes and technologies of subsistence precede population growth.

Nevertheless, the vegetation openness proxy shows increasing land opening throughout our time frame. Based on this finding, it can be argued that population density and impact were higher in the northern parts of our study area at the beginning of the LN (2350–2250 BCE). This finding is also consistent with the KDE models of the geomorphologically defined regions (see section KDE models) and the densities of late battle axes and flint daggers found in grave contexts (see section Aoristic summary).

## Discussion

To reiterate, the present study hypothesises that increased food production and derived population growth were pivotal in changing the widespread cultural diversity of the Middle Neolithic B to cultural unity towards the end of the Late Neolithic. Based on the well-established fact that population increase is a push factor in migrations, our goal was to investigate population dynamics in MNB-LN on both regional and cross-regional levels and thereby be able to discuss the gradual spread of people and, thereby, new cultural traits across Southern Scandinavia.

As evident from the time series presented above, the KDE models and the vegetation openness scores confirm the overall population increase in Southern Scandinavia during the LN, which aligns well with previous population studies discussing the population development in the Neolithic [24,26,29,40]. Although it cannot be directly proven, the concurrency of the population increase identified by our proxies and the described indications of a shift toward more intensified agriculture in the LN strongly support the hypothesised relation between these two phenomena. An important indicator that increased and changed agricultural practices might be responsible for the population growth is given the temporal relation of population density and vegetation openness in SJ. Here, the vegetation opening derived from the high-resolution pollen record from Lake Belau precedes our population peaks.

Our study also shows that the population dynamics, especially the pacing of the growth trajectories, were regionally differentiated and not a simultaneous development. Based on these findings, we suggest the hypotheses that the population increase formed the background of movement of people and thus the spread of LN culture and subsistence practises: Both battle axe ratios and KDE models show that WJ must be considered a core area within the early MNB, where it was strongly influenced by the Corded Ware Complex [31,46,51]. However, the KDE model, the growth rate and the extremely low level of graves with daggers compared to other areas indicate a veritable depopulation of WJ in the early part of the LN. The decline of WJ was accompanied by an increase in activity in EJ and the DI. This shift of the main centre of activity eastwards began as early as 2500 BCE and is most clearly reflected in the KDE models, while the flatter decline in battle axe ratios in EJ compared to WJ points in the same direction. Thus, we see an antagonistic development between WJ and EJ/the DI at the MNB/LN transition. A possible explanation could be that the shift of heightened human activity from west to east from the middle of the MNB to the early LN was driven by migration. A pull factor may have been the general change in subsistence described above: The poorer sandy soils in WJ combined with the cooling trend from 2550 BCE onward did not meet the requirements of the increasing cereal cultivation at the end of the MNB and in the LN. The fact that the vegetation remained open in WJ however suggests that the area was not entirely depopulated but that people who remained maintained the heath through vegetational fires, which is a tool of low labour input for managing extensive heathlands [41,53]. Here, it must again be noted that the overall patterns of rapid peaks and subsequent steady decline in both battle axes and flint daggers ratios can hardly be taken as an indication of population booms and busts but rather as an effect of that grave goods became more diverse over time [91,145]. This is also indicated by the lack of overall correlation between the battle axe/dagger ratio and the KDE models, as well as the vegetation openness scores.

While there is a shift of activity from West to East between the late MNB and early LN, a movement in the opposite direction can be recognised within the later part of the LN reflected in the regional differences in both KDE models and growth rates: In regards to Scania, our study is limited by the lack of both the vegetation openness and the battle axe/dagger proxy, due to non-conclusive differentiation of open vs. forest vegetation, and the lack of compiling catalogues of Scanian MNB and LN burials respectively. However, looking at the KDE model and the growth rates of SB, the area did not face the same population increase at the transition from MNB to LN as in EJ and on the DI. Instead, SB seems to have been characterised by a population boom around 2250–2000 BCE. A similar increase is seen in both KDE models and the openness score on the DI and in EJ, but with a delayed start around 2100 BCE and lasting until around 1900 BCE. Along with the more effective LN agricultural practices, this could be related to the earlier population boom in Scania, which may have been a driver of migration from this area. The described Swedish influence from around 2100 BCE in East Denmark supports increased cultural connectivity between the regions. WJ followed the same development, besides a sudden peak around 2200 BCE, which may be ascribed to uncertainties related to the small radiocarbon dataset from the area. WJ thus seem to finally converge with the rest of the study area in terms of population in the second half of the LN, a trend which has previously been noted by the increase in house remains in the area from the early LN to the late LN [113,146]. We suggest that this alignment of WJ and the rest of our study area marks the final cultural unification of Southern Scandinavia towards the end of the LN. SPDs made on Norwegian material also indicate a population increase in this area in the second half of the LN, albeit somewhat later than in Denmark, c. 2000–1750 BCE [29,94]. This may be related to the same population expansion from Sweden indicated by the Danish material and recent aDNA studies [3,49].

The next question is, why did the detected changes occur at this particular phase of the South Scandinavian prehistory? As mentioned, a recent study by Bunbury et al. (2023) revealed a gradual 1300-year cooling period commencing around 2550 BCE across all four regions. This cooling trend was punctuated by abrupt cooling events around 2350, 2050, and 1650 BCE. While these overarching climate patterns are consistent across our study areas, there appear to be differentiated effects in the regions. While WJ growth rates decline after 2550 BCE, they rise in EJ and on the DI. This may be related to the idea that WJ in more vulnerable to cooling climate, as agricultural production is already limited due to poor sandy soils and possible (over-)exploitation over the last centuries. The identified abrupt cooling events at 2350 and 2050 BCE show no consistent pattern with in the regional population trajectories. However, the population development on the DI is more negatively effected than other regions. Although all regions show positive population events before and after 2050 BCE, the decreasing growth rates around that time indicate that all regions are affected. It is worth mentioning that WJ and the DI seem to be hit more severely regarding climate deterioration than EJ and SB. Further investigations should investigate the relationship between climatic variabilities and regionalized population dynamics.

Nevertheless, this changing population pattern, and especially the increase in population in the LN, cannot be solely explained by climate change. In fact, there is a correlation between a cooling period, an increase in population and agricultural productivity. This corresponds to Boserup’s idea that reaching a landscape’s carrying capacity pressures people to increase food production through intensification and/or technological developments [7]. Thus, we suggest that the changes in subsistence in the LN are a story of human adaptation to climatic deterioration. The climate cooling may, quite paradoxically, ultimately have led to an increase in population in Southern Scandinavia during the LN [144].

Next to adaptation strategies, the study of Großmann et al. [147] on Central European population dynamics shows a population decline around 2250 BCE, possibly induced by a cooling climate. During this time, all Southern Scandinavian regions witnessed an increasing population. If such larger-scale offsets in population developments are related to large-scale migration has yet to be investigated in detail.

Overall, the population decline at the end of the LN, starting around 1900 BCE, as indicated by the KDE models growth rates (Fig 7) and vegetation openness scores (Fig 9), is precursed by a significant drop in summer temperature at 2050 BCE [24] and coincides with a significant and rather sudden drop in Baltic Sea surface temperature between 1900 and 1800 BCE [27]. This population decline may reflect the farmers’ ability to cope with the general cooling trend, and additional abrupt cooling events reached their limit at the end of the LN period, resulting in a population decline. This given situation, not least the marked cooling around 1650 BCE [24], may be understood as one of the reasons for the slow offset of the Nordic Bronze Age. Southern Scandinavia seems not to have fully recovered before 1500 BCE when the Nordic Bronze Age bloomed [131,148].

Climate change is however hardly the only reason for the described intensification of agriculture during the LN of Southern Scandinavia. A part of the explanation may be ascribed to cultural influence from the outside: There is reason to believe that the bifacial sickle was developed in a strongly Bell Beaker-influenced environment in Northern Jutland at the transition from MNB to LN [3,36]. Although cereal production is thought to have been intensified already at the end of the MNB [55,56], this suggests that the intensification of agriculture in the LN is related to the Bell Beaker migrations, which strongly influenced Western Europe around 2450–2350 BCE [149]. As beer-drinking rituals are widely understood as an integral part of the Bell Beaker phenomenon [150,151], the cultivation of cereals associated with beer brewing must have been as much a part of the new practices introduced in Scandinavia around 2350 BCE as the name-giving pottery itself. Furthermore, the introduction of spelt in Southern Scandinavia seems connected to the Bell Beaker phenomenon [55]. Similar is true for Switzerland [152,153], which points to that spelt belonged to an extended version of the Beaker package and was embedded in a vast European exchange network [154,155]. Part of the explanation for the changes in subsistence could thus lie in cultural changes from MNB to LN related to migration and continued contact and exchange with people of the Northwestern periphery of the Bell Beaker phenomenon.

Several other factors, such as ideologies, social organisation and pandemics [1,156–158], to name but a few, may also have affected the MNB/LN population in Southern Scandinavia. An important factor to mention is supra-regional networks associated with the exchange of raw materials such as flint, amber and metal, which we know were widespread in the LN [71,159]. These networks must have included the channels through which knowledge, for instance about new agricultural techniques, flowed. While the Bell Beaker societies on the continent were an early source of new ideas, the contacts to Únětice groups in Central Europe and its northern periphery were particularly influential in the second half of the Late Neolithic [70,77,78]. The famous Nebra sun disc, found in the core area of the Únětice culture, among other things, depicts the rising and setting of the sun at the summer and winter solstices [160]. These astronomical events play a crucial role in shaping the seasonal cycles of agricultural practices, reflecting the importance of cereal cultivation even to the elites of Central European Early Bronze Age societies.

## Conclusions and implications

To summarise, on an overall level, the previously recognised population increase from the MNB to the LN in Southern Scandinavia [24,26,29,40] is supported by our study. The contemporary revival of the agricultural economy, outlined in the introduction, is a strong indicator that population growth and agricultural productivity are linked in the case of the transition from MNB to LN in Southern Scandinavia. The hypothesis of a relation between the population increase and the migration of people inside Scandinavia is also supported by our study. On this basis, it is reasonable to assume that the population increase was a crucial factor in the spread of the new cultural traits associated with the LN and the suggested cultural unification of Southern Scandinavia towards the end of the period. Cultural changes related to contact with and migration of people from the northwestern periphery of the Bell Beaker phenomenon in the MNB/LN transition and adaptations to changes in the climatic environment for agricultural production during the same period are suggested to have kicked off the subsistence shift.

As evident, the suggested migrations within Southern Scandinavia are only made probable by our study, while definite conclusions so far are out of reach. A field of research that may confirm or deny the proposed developments is further genomic and isotopic analyses targeting South Scandinavian LN human material. However, already now, the interpretation of the population development in Southern Scandinavia in MNB and LN and its role in the transformation from the diverse cultural landscape of the MNB to the end of the old east-west divide in Scandinavia in the second half of the Late Neolithic challenges the prevailing interpretation of the MNB as the era when eastern Steppe-derived genes came to dominate in Southern Scandinavia [49]. The Steppe genes were doubtlessly introduced in Scandinavia with the various Corded Ware Culture groups migrating into Western Jutland and Southwestern Sweden around 2800 BCE [46,48]. However, the overall demographic pattern within Scandinavia during the MNB, as presented in the present and other studies [24,26,29], indicates a population decline rather than a rapid increase. Furthermore, the Corded Ware groups, especially the Single Grave Culture, did not replace existing communities in the primary sphere of influence. Instead, the immigrating Corded Ware people settled in Western Jutland, which the scarcity of megaliths in the area shows was sparsely populated during the Funnel Beaker period. While the Single Grave Culture gradually spread to Eastern Jutland, evidenced by the increased distribution of single grave mounds in the later part of the MNB, the Single Grave Culture, as described above, failed to gain foothold on the East Danish Islands, where single grave burial mounds are extremely rare [58,161,162]. Furthermore, the genetic evidence dating to the MNB is not straightforward: While DNA evidence from the Gjerrild grave predominantly reflects Steppe ancestry, other samples reflect a more mixed picture: The sample from Stendrup Hage, dating to the transition between the Funnel Beaker Period and the Middle Neolithic B, shows no influence from Steppe DNA, while two individuals from East Jutland (Toftum Mose) show some Steppe influence, but are found in a bog, which cannot be understood as a context typical for the Single Grave Culture. Three other MNB individuals from Denmark (Klokkehøj, Næs and Kyndeløse) show varying degrees of Steppe ancestry. Still, their association with megalithic structures contradicts the prevailing narrative of an aggressively expanding Corded Ware Culture [54]. The find contexts instead indicate a process of cultural assimilation or potential coercion, wherein people of Steppe ancestry were integrated into the prevailing Funnel Beaker traditions in East Denmark. This interpretation is akin to the recent interpretation of the Vittrup man, who was brought up in a forager community in the northern part of Scandinavia but ended his life among farmers of the Funnel Beaker Culture in Northern Jutland (Fischer et al., 2024). The DNA evidence from MNB Denmark shows that genes and identity are not the same. Although of Steppe ancestry, individuals could still integrate into the local ancient cultural traditions and be laid to rest in a megalith upon passing.

In contrast, the DNA evidence from the LN period is clearer: Here multiple samples show that Steppe ancestry dominated entirely in the South Scandinavian population [49]. Considering the evidence presented above, in particular the evidence of depopulation of the core area of the Single Grave Culture in LN I, it is here proposed that the great turn-over in the gene pool did not happen in the MNB but in the LN: The collapse of the old Single Grave society in Western Jutland, the introduction of a more efficient subsistence and a derived population increase led to the rapid expansion of people and thereby genes, and new cultural habits throughout Southern Scandinavia during the LN. The great turnover of the gene pool in Southern Scandinavia was thus not necessarily violent. People of Steppe ancestry may simply have outnumbered people of the old Anatolian-derived ancestry through the Late Neolithic population boom and the derived expansions.

## Supporting information

S1 Supporting information **S1 File. SI_C01_SPD_KDE_models.** R-script for analysing radiocarbon dates dates. The code performs the computation of over-regional and regional SPD and KDE models, as well as their export to CSV files (Rmd). **S2 File. SI_C02_aoristic_dating.** R-script for exporting aoristic time series derived from typochronological dated archaeological material as CSV files (Rmd). **S3 File. SI_C03_vegetation_openness_score_example.** R-script performing the computation of a vegetation openness score from pollen records and the export of the generated time series as CVS file (Rmd). **S4 File. SI_C04_data_preparation.** Jupyter Notebook performing the import and transformation of relevant data visualize plots exhibited in the paper (ipynb). **S5 File. SI_C05_figures_extra.** Jupyter Notebook visualizing the plots exhibited in the paper (ipynb). **S1 Data. SI_D01_reg_data_no_dups.** Spread sheet holding radiocarbon dates, with the information of laboratory identification, site name, geographical coordinates, site type, material, source and regional affiliation (csv). **S2 Data. SI_D02_reg_axe_dagger_graves.** Spread sheet holding entries of axes and daggers, with the information of context, site, parish, artefact identification, type, subtype, absolute dating, typochonological dating, references, geographical coordinates and regional affiliations (csv). **S3 Data. SI_D03_pollen_example.** Spread sheet holding sample entries of the pollen records from Krageholm (neotoma Site ID 3204) and Bjäresjöholmsjön (neotoma Site ID 3017) for example run of S3 File. Record can be access via the neotoma explorer (https://apps.neotomadb.org/explorer/) with their given IDs. Each entry holds the information of the records type, regional affiliation, absolute BP and BCE dating, as well as the counts of given plant taxa (csv). **S4 Data. SI_D04_PAP_303600_TOC_LOI.** Table holding sample entries of TOC content, LOI and SST reconstruction of sediment core PAP_303600 for correlations of population development with Baltic sea surface temperature. Available via 10.1594/PANGAEA.883292 (tab). **S5 Data. SI_D05_vos_[…].** Spread sheets holding the vegetation openness score time series of lake Belau, Vinge, Northern Jutland and Zealand (csv).
(ZIP)
