# Supplementary material for: Subsistence and population development from the Middle Neolithic B (2800–2350 BCE) to the Late Neolithic (2350–1700 BCE) in Southern Scandinavia
Source: PLoS One. 2024 Oct 28;19(10):e0301938. doi: 10.1371/journal.pone.0301938 (PMC11516014; doi:10.1371/journal.pone.0301938)
Supplement: S1 Supporting information — S1 File. SI_C01_SPD_KDE_models. R-script for analysing radiocarbon dates dates. The code performs the computation of over-regional and regional SPD and KDE models, as well as their export to CSV files (Rmd). S2 File. SI_C02_aoristic_dating. R-script for exporting aoristic time series derived from typochronological dated archaeological material as CSV files (Rmd). S3 File. SI_C03_vegetation_openness_score_example. R-script performing the computation of a vegetation openness score from pollen records and the export of the generated time series as CVS file (Rmd). S4 File. SI_C04_data_preparation. Jupyter Notebook performing the import and transformation of relevant data visualize plots exhibited in the paper (ipynb). S5 File. SI_C05_figures_extra. Jupyter Notebook visualizing the plots exhibited in the paper (ipynb). S1 Data. SI_D01_reg_data_no_dups. Spread sheet holding radiocarbon dates, with the information of laboratory identification, site name, geographical coordinates, site type, material, source and regional affiliation (csv). S2 Data. SI_D02_reg_axe_dagger_graves. Spread sheet holding entries of axes and daggers, with the information of context, site, parish, artefact identification, type, subtype, absolute dating, typochonological dating, references, geographical coordinates and regional affiliations (csv). S3 Data. SI_D03_pollen_example. Spread sheet holding sample entries of the pollen records from Krageholm (neotoma Site ID 3204) and Bjäresjöholmsjön (neotoma Site ID 3017) for example run of S3 File. Record can be access via the neotoma explorer (https://apps.neotomadb.org/explorer/) with their given IDs. Each entry holds the information of the records type, regional affiliation, absolute BP and BCE dating, as well as the counts of given plant taxa (csv). S4 Data. SI_D04_PAP_303600_TOC_LOI. Table holding sample entries of TOC content, LOI and SST reconstruction of sediment core PAP_303600 for correlations of population development with Baltic sea surface t [file pone.0301938.s001.zip › support_information/SI_C01_SPD_KDE_models.html]

Relative population modelling - SPD and KDE models


# Relative population modelling - SPD and KDE models

#### Julian Laabs

This Supplementary Information belongs to the study
**“Subsistence and Population developments from the late Middle to
the Late Neolithic in Southern Scandinavia”** (DOI:
**tba**). Please consider the publication for context and
content related information.

This script has profited very much from the supplementary information
of Palmisano et al. (2021) **“Long-Term Demographic Trends in
Prehistoric Italy: Climate Impacts and Regionalised Socio-Ecological
Trajectories”** (DOI: https://doi.org/10.1007/s10963-021-09159-3) and the
supplementary information of Parkinson et al. (2021)
**“Radiocarbon Dated Trends and Central Mediterranean
Prehistory”** (DOI: https://doi.org/10.1007/s10963-021-09158-4)

For deeper insights into the methods used here one should consult
Crema (2022), Crema and Bevan (2021), McLaughlin (2019) and the sources
referenced in their studies.

The script performs the computation of summed probability
distributions (SPD) and kernel density estimation models from
radiocarbon dates.

## Preparation

Load needed packages.

```
library(here)
library(rcarbon)
```

The original data is imported and sub-setted by conditions to enhance
reliability of the models. E.g. dates with high standard deviation are
cropped, as well as data from secure marine origin.

```
# Load data
all_14C <- read.csv("./data/data_raw/reg_data_no_dups.csv", header=TRUE, 
                    stringsAsFactors=FALSE, encoding="UTF-8", 
                    na.strings=c("NA",""), strip.white=TRUE)

# Delete dates with too high std
all_14C <- subset(all_14C, all_14C$std<100)

# Delete dates from material with high probability of marine reservoir effects
all_14C <- subset(all_14C, all_14C$material != "shell")
```

Set general parameters reoccuring in the analysis.

```
### General SPD parameters -----------------------------------------------------
runm <- 25 # running mean (smoother) in years
nsim <- 100 # number of simulations for exponential/permutation models
ncores <- 4 # use n cores of the processor, if possible
binh <- 100 # degree of binning on-site level
kdesim = 1000 # number of simulations for KDE models
kdebw = 50 # bandwidth (years) used for KDE models

# Start and end of chronological working range
realstartBP <- 4800 #2850 BCE
realendBP <- 3650 #1750 BCE
bracket <- 100
workingstartBP <- realstartBP+bracket
workingendBP <- realendBP-bracket
if (workingendBP<0){ workingendBP <- 0 }
```

## Over-regional dataset

### All sites

#### Basic summed probability distribution (SPD)

Perform calibration of the radiocarbon dates and sum calibration,
save the density distribution to a .csv file and visualize the results.
Note that we do not normalize our data (see further information see:
Cream and Bevan 2021).

```
# Calibration
all_14C.caldates = calibrate(x=all_14C$bp, errors=all_14C$std, 
                             calCurves='intcal20', method="standard", 
                             normalised=FALSE, ncores=ncores, calMatrix=TRUE)

# Calculating SPD
all_14C.spd = spd(all_14C.caldates, timeRange=c(workingstartBP,workingendBP), 
                  datenormalised=FALSE) 

# Write not normalized SPD to CSV
write.csv(all_14C.spd$grid,"./data/data_derived/table/all_all_spd.csv")

# Plot it
plot(all_14C.spd, main="Non-normalized Sum (all)") 
plot(all_14C.spd, runm=runm, add=TRUE,type="simple",col="darkorange", lwd=1.5, 
     lty=2)
```

##### Subset data to time frame

Cropping data set to time frame defined with the initial
parameters.

```
# Subset data
x <- all_14C.caldates$calmatrix[(55000-workingstartBP):(55000-workingendBP),]
all_14C <- all_14C[colSums(x) > 0.0,]

# Write meta data into CSV
meta = data.frame(ndates=NA)
meta$ndates <- nrow(all_14C)
write.csv(meta, "./data/data_derived/meta/meta_all_all_spd.csv")

# Not normalized - Calibration with reduced, but relevant data set
all_14C.caldates = calibrate(x=all_14C$bp, errors=all_14C$std, 
                             calCurves='intcal20', method="standard", 
                             normalised=FALSE, ncores=ncores, calMatrix=TRUE)
```

Create df holding each site with their number of dates to calculate
mean dates per site.

```
# Drop duplicates
site_14C <- all_14C[!duplicated(all_14C$site_name), ]

# Add column holding dates per site  
site_14C$n_dates <- 0

# Iterate over site_14C df and sum number of dates for each site from all_14C df
for(s in site_14C$site_name){
  site_14C[site_14C$site_name == s,]$n_dates = length(all_14C[all_14C$site_name == s,]$lab)
}
```

Create table for publication (Tab.1) counting, the number of dates of
sites, settlements and burials for all regions and the whole data set.
Further, calculate mean dates per site for each site type and all
regions.

```
# Create empty df
Tab1 <- data.frame(matrix(nrow = 5, ncol = 9), 
                   row.names = c("Meta region","Scania and Bornholm", 
                                 "Danish Isles","Eastern Jutland",
                                 "Western Jutland"))
colnames(Tab1) = c("n_date_all","n_date_s","n_date_b",
                   "n_site_all","n_site_s","n_site_b",
                   "mean_ds_all","mean_ds_s","mean_ds_b")

# Add number of dates for whole region
Tab1["Meta region",]$n_date_all <- nrow(all_14C)
Tab1["Meta region",]$n_date_s <- nrow(subset(all_14C, 
                                             all_14C$site_type == "settlement"))
Tab1["Meta region",]$n_date_b <- nrow(subset(all_14C, 
                                             all_14C$site_type == "burial"))

# Add number of sites for whole region
Tab1["Meta region",]$n_site_all <- nrow(site_14C)
Tab1["Meta region",]$n_site_s <- nrow(subset(site_14C, 
                                      site_14C$site_type == "settlement"))
Tab1["Meta region",]$n_site_b <- nrow(subset(site_14C, 
                                      site_14C$site_type == "burial"))

# Add mean number of dates per site for whole region
Tab1["Meta region",]$mean_ds_all <- mean(site_14C$n_dates)
Tab1["Meta region",]$mean_ds_s <- mean(subset(site_14C, 
                                       site_14C$site_type == "settlement")$n_dates)
Tab1["Meta region",]$mean_ds_b <- mean(subset(site_14C, 
                                       site_14C$site_type == "burial")$n_dates)


for(reg in c("Scania and Bornholm", "Danish Isles","Eastern Jutland",
             "Western Jutland")){
  # Subset data sets to regional affiliation
  xdf <- all_14C[all_14C$region_geom_2 == reg,]
  ydf <- site_14C[site_14C$region_geom_2 == reg,]
  
  # Add number of dates for region reg
  Tab1[reg,]$n_date_all <- nrow(xdf)
  Tab1[reg,]$n_date_s <- nrow(subset(xdf, xdf$site_type == "settlement"))
  Tab1[reg,]$n_date_b <- nrow(subset(xdf, xdf$site_type == "burial"))
  
  # Add number of sites for region reg
  Tab1[reg,]$n_site_all <- nrow(ydf)
  Tab1[reg,]$n_site_s <- nrow(subset(ydf, ydf$site_type == "settlement"))
  Tab1[reg,]$n_site_b <- nrow(subset(ydf, ydf$site_type == "burial"))
  
  # Add mean number of dates per site for region reg
  Tab1[reg,]$mean_ds_all <- mean(ydf$n_dates)
  Tab1[reg,]$mean_ds_s <- mean(subset(ydf, 
                                      ydf$site_type == "settlement")$n_dates)
  Tab1[reg,]$mean_ds_b <- mean(subset(ydf,
                                      ydf$site_type == "burial")$n_dates)
}

# Write table to .csv file
write.csv(Tab1,"./data/data_derived/table/Tab1.csv")
```

#### Binning

Explore binning levels on the sum calibration.

```
# Bin sense
binsense(x=all_14C.caldates, y=all_14C$site_name, h=seq(0,500,50), 
         timeRange=c(workingstartBP,workingendBP))
```

Bin data on site level and perform sum calibration with a resonable
bin level.

```
# Bin data
all_14C.bins = binPrep(sites=all_14C$site_name, ages=all_14C$bp, h=binh)

# Binned SPD
all_14C.spd.bins = spd(all_14C.caldates, bins=all_14C.bins, 
                       timeRange=c(workingstartBP,workingendBP), 
                       datenormalised=FALSE, runm=runm)

# Write binned SPD to CSV
write.csv(all_14C.spd.bins$grid, 
          "./data/data_derived/table/all_all_spd_bin.csv")

# Write meta data into CSV
meta = data.frame(ndates=NA,nbins=NA,nsites=NA)
meta$ndates <- all_14C.spd.bins$metadata$ndates
meta$nbins<- all_14C.spd.bins$metadata$nbins
meta$nsites <- length(unique(all_14C$site_name))
write.csv(meta,"./data/data_derived/meta/meta_all_all_spd_bin.csv")

# Plot it
plot(all_14C.spd.bins, main="Non-normalized binned Sum (all)")
plot(all_14C.spd.bins, runm=runm, add=TRUE,type="simple", col="darkorange", 
     lwd=1.5, lty=2)
```

Calculate and visualize median dates of all radiocarbon dates the on
the binned SPD.

```
all_14C.bins.med=binMed(x = all_14C.caldates, bins=all_14C.bins)

# Plot it
plot(all_14C.spd.bins, runm=runm, main="Non-normalized binned Sum (all)")
barCodes(all_14C.bins.med, yrng=c(0,0.05))
```

#### Thinning

Thin the radiocarbon dates and perform sum calibration. If the
original data is well distributed a thinned version of the SPD should
not diverge badly from the whole data set.

```
# Subset caldates object based on random thinning
all_14C.caldates2 = all_14C.caldates[thinDates(ages=all_14C$bp, 
                                               errors=all_14C$std, 
                                               bins=all_14C.bins, size=1, 
                                               method='random')]

# Aggregation and visualization of the SPD
all_14C.spd.thinned = spd(all_14C.caldates2, 
                          timeRange=c(workingstartBP,workingendBP))

# Write thinned SPD to CSV
write.csv(all_14C.spd.thinned$grid,
          "./data/data_derived/table/all_all_spd_bin_thin.csv")

# Write meta data into CSV
meta = data.frame(ndates=NA,nbins=NA)
meta$ndates <- all_14C.spd.thinned$metadata$ndates
meta$nbins<- all_14C.spd.thinned$metadata$nbins
write.csv(meta, "./data/data_derived/meta/meta_all_all_spd_bin_thin.csv")

# Plot it
plot(all_14C.spd.thinned, main="Non-normalized thinned Sum (all)")
plot(all_14C.spd.thinned, runm=runm, add=TRUE,type="simple",col="darkorange", 
     lwd=1.5, lty=2)
```

Calculate the Pearson correlation coefficient between the thinned and
original SPDs of calibrated radiocarbon dates.

```
cor.test(all_14C.spd$grid$PrDens, all_14C.spd.thinned$grid$PrDens, 
         method="pearson")
```

```
## 
##  Pearson's product-moment correlation
## 
## data:  all_14C.spd$grid$PrDens and all_14C.spd.thinned$grid$PrDens
## t = 84.186, df = 1349, p-value < 2.2e-16
## alternative hypothesis: true correlation is not equal to 0
## 95 percent confidence interval:
##  0.9076008 0.9246978
## sample estimates:
##       cor 
## 0.9165672
```

#### Short-lived material

Subset the data to radiocarbon dates from only short-lived material
and perform sum calibration. If the SPD of short-lived material is
similar to the over all SPD, one can argue that the influence of
long-lived material is marginal.

```
# Subset dates from short-lived material 
short <-all_14C[grep(paste(c("plant remains","bone","food remains","wood",
                             "shell", "textile"), 
                           collapse="|"), all_14C$material),] 
# Calibrate, bin, SPD
short.caldates = calibrate(x=short$bp, errors=short$std, calCurves='intcal20',
                           method="standard", normalised=FALSE, ncores=ncores, 
                           calMatrix=TRUE)
short.bins = binPrep(sites=short$site_name, ages=short$bp, h=binh)
short.spd.bins = spd(short.caldates, bins=short.bins, 
                       timeRange=c(workingstartBP,workingendBP), 
                       datenormalised=FALSE, runm=runm)

# Write short-lived SPD to CSV
write.csv(short.spd.bins$grid, 
          "./data/data_derived/table/all_all_spd_bin_short.csv")

# Write meta data into CSV
meta = data.frame(ndates=NA,nbins=NA,nsites=NA)
meta$ndates <- short.spd.bins$metadata$ndates
meta$nbins<- short.spd.bins$metadata$nbins
meta$nsites <- length(unique(short$site_name))
write.csv(meta, "./data/data_derived/meta/meta_all_all_spd_bin_short.csv")

# Plot it
plot(short.spd.bins, 
     main="Non-normalized binned Sum (only short-lived material)")
plot(short.spd.bins, runm=runm, add=TRUE,type="simple",col="darkorange", 
     lwd=1.5, lty=2)
```

Calculate the Pearson correlation coefficient between the short-lived
and all SPDs of calibrated radiocarbon dates.

```
cor.test(short.spd.bins$grid$PrDens, all_14C.spd.bins$grid$PrDens, 
         method="pearson")
```

```
## 
##  Pearson's product-moment correlation
## 
## data:  short.spd.bins$grid$PrDens and all_14C.spd.bins$grid$PrDens
## t = 77.197, df = 1349, p-value < 2.2e-16
## alternative hypothesis: true correlation is not equal to 0
## 95 percent confidence interval:
##  0.8926631 0.9123972
## sample estimates:
##       cor 
## 0.9030054
```

#### (Composite) Kernel Density Estimation

Perform KDE modelling of radiocarbon dates with 1000 simulations.

```
# Sample dates and perform KDE 
all_14C.randates = sampleDates(all_14C.caldates, bins=all_14C.bins, nsim=kdesim, 
                               boot=TRUE)
all_14C.ckde = ckde(all_14C.randates, timeRange=c(workingstartBP,workingendBP),
                    bw=kdebw)

# Write KDE model to CSV and add time vector as first column
kde_matrix = cbind(seq(workingstartBP,workingendBP),all_14C.ckde$res.matrix) 
kde_matrix[is.na(kde_matrix)] <- 0 # Replace NAs with 0.0
write.csv(kde_matrix, "./data/data_derived/table/all_all_spd_bin_kde.csv")

# Write meta data into CSV
meta = data.frame(ndates=NA,nbins=NA,nsites=NA,nsim=kdesim,bw=kdebw)
meta$ndates <- all_14C.spd.bins$metadata$ndates
meta$nbins<- all_14C.spd.bins$metadata$nbins
meta$nsites <- length(unique(all_14C$site_name))
write.csv(meta, "./data/data_derived/meta/meta_all_all_spd_bin_kde.csv")

# Plot it
matplot(seq(workingstartBP,workingendBP)*-1,
        as.data.frame(all_14C.ckde$res.matrix), type="l", xlab="BP",
        main = "cKDE (all)")
```

#### Hypotheses testing: Exponential growth model

Perform computation of an exponential model against which the binned
SPD can be compared. The exp. model expects an exponential growth from
the start of our time window until the end of it. Positive and negative
deviation of the SPD against that model can be interpreted as times of
extraordinary high (red) respectively low (blue) density.

```
# Exponential model 
expnull_all_14C <- modelTest(all_14C.caldates, errors=all_14C$std, 
                             bins=all_14C.bins, nsim=nsim, 
                             timeRange=c(workingstartBP,workingendBP), 
                             model="exponential", runm=runm, 
                             method="uncalsample", ncores=ncores,
                             datenormalised=FALSE)

# Save results to independent data frame
exp_result = expnull_all_14C$result
names(exp_result)[3] <- 'env_min'
names(exp_result)[4] <- 'env_max'

# Write perm. tested binned burial SPD to CSV
write.csv(exp_result, "./data/data_derived/table/all_all_spd_bin_exp.csv")

# Write meta data into CSV
meta = data.frame(ndates=NA,nbins=NA,nsites=NA,p=NA)
meta$ndates <- all_14C.spd.bins$metadata$ndates
meta$nbins<- all_14C.spd.bins$metadata$nbins
meta$nsites <- length(unique(all_14C$site_name))
meta$p <- expnull_all_14C$pval
write.csv(meta, "./data/data_derived/meta/meta_all_all_spd_bin_exp.csv")

# Plot it
plot(expnull_all_14C, main="Non-normalized binned Sum (all) against Exp-Model")
```

Examine global fit of the SPD from exponential model.

```
expnull_all_14C$pval #global p-value
```

```
## [1] 0.00990099
```

Examine significant deviations of the SPD from exponential model.

```
# Testing Local Growth Rates
summary(expnull_all_14C,type='roc')
```

```
## 'modelTest()' function summary:
## 
## Number of radiocarbon dates: 2060
## Number of bins: 1187
## Backsight size: 50
## 
## Statistical Significance computed using 100 simulations. 
## Global p-value (rate of change): 0.0297.
## 
## Signficant positive local deviations at:
## 4812~4785 BP 
## 3772~3766 BP 
## 
## Significant negative local deviations at:
## 4850 BP 
## 3839~3779 BP 
## 3735~3715 BP 
## 3631~3550 BP
```

Visualize rate of change of the SP and exponential model with
significant deviations.

```
# Plot ROC
plot(expnull_all_14C,type='roc', main="Rate of Change")
```

### Burial sites

Repeating the computation of binned SPD and KDE models for dates from
burial contexts.

#### Binned SPDs

```
# ------------------------------------------------------------------------------
# Subset all burial dates
bur_14C <- subset(all_14C, all_14C$site_type=="burial")

# Calibrate and SPD
bur_14C.caldates=calibrate(x=bur_14C$bp, errors=bur_14C$std, 
                           calCurves='intcal20', method="standard", 
                           normalised=FALSE, ncores=ncores, calMatrix=TRUE)
bur_14C.spd = spd(bur_14C.caldates, timeRange=c(workingstartBP,workingendBP), 
                  datenormalised=FALSE) 

# Write not normalized SPD to CSV
write.csv(bur_14C.spd$grid, "./data/data_derived/table/all_bur_spd.csv")

# Binned SPD
bur_14C.bins = binPrep(sites=bur_14C$site_name, ages=bur_14C$bp, h=binh)
bur_14C.spd.bins = spd(bur_14C.caldates, bins=bur_14C.bins,
                       timeRange=c(workingstartBP,workingendBP), 
                       datenormalised=FALSE, runm=runm)

# Write binned SPD to CSV
write.csv(bur_14C.spd.bins$grid, "./data/data_derived/table/all_bur_spd_bin.csv")

# Write meta data into CSV
meta = data.frame(ndates=NA,nbins=NA,nsites=NA)
meta$ndates <- bur_14C.spd.bins$metadata$ndates
meta$nbins<- bur_14C.spd.bins$metadata$nbins
meta$nsites <- length(unique(bur_14C$site_name))
write.csv(meta, "./data/data_derived/meta/meta_all_bur_spd_bin.csv")

# Plot it
plot(bur_14C.spd.bins, main="Non-normalized binned Sum (burials)")
plot(bur_14C.spd.bins, runm=runm, add=TRUE, type="simple", col="darkorange", 
     lwd=1.5, lty=2)
```

#### KDE

```
# Composite kernel density estimates (CKDE)
bur_14C.randates = sampleDates(bur_14C.caldates, bins=bur_14C.bins, nsim=kdesim, 
                               boot=TRUE)
bur_14C.ckde = ckde(bur_14C.randates, timeRange=c(workingstartBP,workingendBP),
                    bw=kdebw)

# Write KDE model to CSV and add time vector as first column
kde_matrix = cbind(seq(workingstartBP,workingendBP),bur_14C.ckde$res.matrix) 
kde_matrix[is.na(kde_matrix)] <- 0 # Replace NAs with 0.0
write.csv(kde_matrix, "./data/data_derived/table/all_bur_spd_bin_kde.csv")

# Write meta data into CSV
meta = data.frame(ndates=NA,nbins=NA,nsites=NA,nsim=kdesim,bw=kdebw)
meta$ndates <- bur_14C.spd.bins$metadata$ndates
meta$nbins<- bur_14C.spd.bins$metadata$nbins
meta$nsites <- length(unique(bur_14C$site_name))
write.csv(meta, "./data/data_derived/meta/meta_all_bur_spd_bin_kde.csv")

# Plot it
matplot(seq(workingstartBP,workingendBP)*-1,
        as.data.frame(bur_14C.ckde$res.matrix), type="l", xlab="BP", 
        main = "cKDE (burials)")
```

### Settlement sites

Repeating the computation of binned SPD and KDE models, as well as
testing against an exponential model for dates from settlement
contexts.

#### Binned SPDs

```
# ------------------------------------------------------------------------------
# Subset all settlements dates
set_14C <- subset(all_14C, all_14C$site_type=="settlement")

# Calibrate and SPD
set_14C.caldates=calibrate(x=set_14C$bp, errors=set_14C$std, 
                           calCurves='intcal20', method="standard", 
                           normalised=FALSE, ncores=ncores, calMatrix=TRUE)
set_14C.spd = spd(set_14C.caldates, timeRange=c(workingstartBP,workingendBP), 
                  datenormalised=FALSE)

# Write not normalized SPD to CSV
write.csv(set_14C.spd$grid, "./data/data_derived/table/all_set_spd.csv")

# Binned SPD
set_14C.bins = binPrep(sites=set_14C$site_name, ages=set_14C$bp, h=binh)
set_14C.spd.bins = spd(set_14C.caldates, bins=set_14C.bins,
                       timeRange=c(workingstartBP,workingendBP), 
                       datenormalised=FALSE, runm=runm)

# Write binned SPD to CSV
write.csv(set_14C.spd.bins$grid, 
          "./data/data_derived/table/all_set_spd_bin.csv")

# Write meta data into CSV
meta = data.frame(ndates=NA,nbins=NA,nsites=NA)
meta$ndates <- set_14C.spd.bins$metadata$ndates
meta$nbins<- set_14C.spd.bins$metadata$nbins
meta$nsites <- length(unique(set_14C$site_name))
write.csv(meta, "./data/data_derived/meta/meta_all_set_spd_bin.csv")

# Plot it
plot(set_14C.spd.bins)
plot(set_14C.spd.bins, runm=runm, add=TRUE,type="simple", col="darkorange", 
     lwd=1.5, lty=2)
```

Calculate the Pearson correlation coefficient between the burial and
settlemnt SPDs of calibrated radiocarbon dates.

```
cor.test(set_14C.spd.bins$grid$PrDens, bur_14C.spd.bins$grid$PrDens, 
         method="pearson")
```

```
## 
##  Pearson's product-moment correlation
## 
## data:  set_14C.spd.bins$grid$PrDens and bur_14C.spd.bins$grid$PrDens
## t = 9.9518, df = 1349, p-value < 2.2e-16
## alternative hypothesis: true correlation is not equal to 0
## 95 percent confidence interval:
##  0.2111376 0.3105263
## sample estimates:
##      cor 
## 0.261525
```

#### KDE

```
# Composite kernel density estimates (CKDE)
set_14C.randates = sampleDates(set_14C.caldates, bins=set_14C.bins, nsim=kdesim, 
                               boot=TRUE)
set_14C.ckde = ckde(set_14C.randates, timeRange=c(workingstartBP,workingendBP),
                    bw=kdebw)

# Write KDE model to CSV and add time vector as first column
kde_matrix = cbind(seq(workingstartBP,workingendBP),set_14C.ckde$res.matrix) 
kde_matrix[is.na(kde_matrix)] <- 0 # Replace NAs with 0.0
write.csv(kde_matrix, "./data/data_derived/table/all_set_spd_bin_kde.csv")

# Write meta data into CSV
meta = data.frame(ndates=NA,nbins=NA,nsites=NA,nsim=kdesim,bw=kdebw)
meta$ndates <- set_14C.spd.bins$metadata$ndates
meta$nbins<- set_14C.spd.bins$metadata$nbins
meta$nsites <- length(unique(set_14C$site_name))
write.csv(meta, "./data/data_derived/meta/meta_all_set_spd_bin_kde.csv")

# Plot it
matplot(seq(workingstartBP,workingendBP)*-1,
        as.data.frame(set_14C.ckde$res.matrix), type="l", xlab="BP", 
        main = "cKDE (settlement)")
```

#### Hypotheses testing: Exponential growth model

The testing of the settlement SPD against an exponential model is
performed because settlements are argued to be a more direct proxy of
population density.

```
# Exponential model 
expnull_set_14C <- modelTest(set_14C.caldates, errors=set_14C$std, 
                             bins=set_14C.bins, nsim=nsim, 
                             timeRange=c(workingstartBP,workingendBP), 
                             model="exponential", runm=runm, 
                             method="uncalsample", ncores=ncores,
                             datenormalised=FALSE)

# Save results to independent data frame
exp_result = expnull_set_14C$result
names(exp_result)[3] <- 'env_min'
names(exp_result)[4] <- 'env_max'

# Write perm. tested binned burial SPD to CSV
write.csv(exp_result, "./data/data_derived/table/all_set_spd_bin_exp.csv")

# Write meta data into CSV
meta = data.frame(ndates=NA,nbins=NA,nsites=NA,p=NA)
meta$ndates <- set_14C.spd.bins$metadata$ndates
meta$nbins<- set_14C.spd.bins$metadata$nbins
meta$nsites <- length(unique(set_14C$site_name))
meta$p <- expnull_set_14C$pval
write.csv(meta, "./data/data_derived/meta/meta_all_set_spd_bin_exp.csv")

# Plot it
plot(expnull_set_14C, main="Non-normalized binned Sum (set) against Exp-Model")
```

Examine global fit of the SPD from exponential model.

```
expnull_set_14C$pval #global p-value
```

```
## [1] 0.00990099
```

Examine significant deviations of the SPD from exponential model.

```
summary(expnull_set_14C)
```

```
## 'modelTest()' function summary:
## 
## Number of radiocarbon dates: 1469
## Number of bins: 864
## 
## Statistical Significance computed using 100 simulations. 
## Global p-value: 0.0099.
## 
## Signficant positive local deviations at:
## 3986~3836 BP 
## 
## Significant negative local deviations at:
## 4857~4832 BP 
## 3634~3550 BP
```

## Regionalized analysis: KDE models

Define columns holding regional affiliation. In regard to our study
we compute KDE models for the regional division based on
geomorphological characteristics of our study area (“region\_geom\_2”) and
the differentiation by pollen record reference regions
(“region\_geom\_3”).

```
# Create list of columns names
j_lst = c("region_geom_2", "region_geom_3")
```

The modelling of KDE models will be repeated for the full data set
without distinguishing the site type, as well as for a subset of all
settlement and all burial data.

### All sites

The following code chunk performs the sub-setting of the original
data set to to those dates lying in the current region passed to the
loop. These dates are calibrated (non-normalized), binned (binh=100) and
passed on to the KDE function.

Next to the current regional’s KDE model a KDE model is simulated
that includes all other regions except the one currently passed to the
loop. Those two KDE models can be used for the detection of positive or
negative deviation of regional trajectories from the over-all
development. Such an approach is in its idea similar to the mark
permutation tests applied to regional SPDs (Crema and Bevan 2021;
Parkinson et al. 2021).

For both types of KDE models the meta data (number of dates, number
of sites, number of bins) of each model will be saved in a table and
exported as .csv file. The actual KDE model is saved as .csv file
too.

```
# Loop over all given regional affiliation columns
for (j in j_lst){
  # List unique regions in region column
  region_lst = unique(all_14C[,j])
  # Loop over region list
  for (i in region_lst){
    # Subset whole data set to current region
    reg_14C <- subset(all_14C, all_14C[,j]==i)
    
    # Calibrate dates
    reg_14C.caldates=calibrate(x=reg_14C$bp, errors=reg_14C$std, 
                               calCurves='intcal20', method="standard", 
                               normalised=FALSE, ncores=ncores, calMatrix=TRUE)
    
    # Bin dates
    reg_14C.bins = binPrep(sites=reg_14C$site_name, ages=reg_14C$bp, h=binh)
    
    # Binned SPD
    reg_14C.spd.bins = spd(reg_14C.caldates, bins=reg_14C.bins, 
                       timeRange=c(workingstartBP,workingendBP), 
                      datenormalised=FALSE, runm=runm)
    
    #Composite kernel density estimates (KDE)
    reg_14C.randates = sampleDates(reg_14C.caldates, bins=reg_14C.bins, 
                                   nsim=kdesim, boot=TRUE)
    reg_14C.ckde = ckde(reg_14C.randates, 
                        timeRange=c(workingstartBP,workingendBP), bw=kdebw)
    
    # Write KDE model to CSV and add time vector as first column
    kde_matrix = cbind(seq(workingstartBP,workingendBP),reg_14C.ckde$res.matrix)
    kde_matrix[is.na(kde_matrix)] <- 0 # Replace NAs with 0.0
    write.csv(kde_matrix, paste0("./data/data_derived/table/",j,"_",i,
                                "_all_spd_bin_kde.csv"))
    
    # Write meta data into CSV
    meta = data.frame(ndates=NA,nbins=NA,nsites=NA,nsim=kdesim,bw=kdebw)
    meta$ndates <- reg_14C.spd.bins$metadata$ndates
    meta$nbins<- reg_14C.spd.bins$metadata$nbins
    meta$nsites <- length(unique(reg_14C$site_name))
    write.csv(meta, paste0("./data/data_derived/meta/meta_",j,"_",i,
                          "_all_spd_bin_kde.csv"))
# ------------------------------------------------------------------------------ 
    # Subset whole data set to NOT current region
    not_reg_14C <- subset(all_14C, all_14C[,j]!=i)
    
    # Calibrate dates
    not_reg_14C.caldates=calibrate(x=not_reg_14C$bp, errors=not_reg_14C$std, 
                               calCurves='intcal20', method="standard", 
                               normalised=FALSE, ncores=ncores, calMatrix=TRUE)
    
    # Bin dates
    not_reg_14C.bins = binPrep(sites=not_reg_14C$site_name, ages=not_reg_14C$bp,
                               h=binh)
    
    # Binned SPD
    not_reg_14C.spd.bins = spd(not_reg_14C.caldates, bins=not_reg_14C.bins,
                               timeRange=c(workingstartBP,workingendBP),
                               datenormalised=FALSE, runm=runm)
    
    # Composite kernel density estimates (KDE)
    not_reg_14C.randates = sampleDates(not_reg_14C.caldates, 
                                       bins=not_reg_14C.bins, nsim=kdesim, 
                                       boot=TRUE)
    not_reg_14C.ckde = ckde(not_reg_14C.randates, 
                        timeRange=c(workingstartBP,workingendBP), bw=kdebw)
    
    # Write KDE model to CSV and add time vector as first column
    kde_matrix = cbind(seq(workingstartBP,workingendBP),
                       not_reg_14C.ckde$res.matrix) 
    kde_matrix[is.na(kde_matrix)] <- 0 # Replace NAs with 0.0
    write.csv(kde_matrix, paste0("./data/data_derived/table/",j,"_not_",i,
                                "_all_spd_bin_kde.csv"))
    
    # Write meta data into CSV
    meta = data.frame(ndates=NA,nbins=NA,nsites=NA,nsim=kdesim,bw=kdebw)
    meta$ndates <- not_reg_14C.spd.bins$metadata$ndates
    meta$nbins<- not_reg_14C.spd.bins$metadata$nbins
    meta$nsites <- length(unique(not_reg_14C$site_name))
    write.csv(meta, paste0("./data/data_derived/meta/meta_",j,"_not_",i,
                          "_all_spd_bin_kde.csv"))
  }
}
```

### Burial sites

Repeat procedure for a subset of burial sites.

```
for (j in j_lst){
  region_lst = unique(bur_14C[,j])
  for (i in region_lst){
    print(i)
    bkde_14C <- subset(bur_14C, bur_14C[,j]==i)
    bkde_14C.caldates=calibrate(x=bkde_14C$bp, errors=bkde_14C$std, 
                                calCurves='intcal20', method="standard", 
                                normalised=FALSE, ncores=ncores, 
                                calMatrix=TRUE)
    bkde_14C.bins = binPrep(sites=bkde_14C$site_name, ages=bkde_14C$bp, h=binh)
    bkde_14C.spd.bins = spd(bkde_14C.caldates, bins=bkde_14C.bins,
                            timeRange=c(workingstartBP,workingendBP),
                            datenormalised=FALSE, runm=runm)
    bkde_14C.randates = sampleDates(bkde_14C.caldates, bins=bkde_14C.bins, 
                                    nsim=kdesim,boot=TRUE)
    bkde_14C.ckde = ckde(bkde_14C.randates, 
                         timeRange=c(workingstartBP,workingendBP),bw=kdebw)
    kde_matrix = cbind(seq(workingstartBP,workingendBP),
                       bkde_14C.ckde$res.matrix) 
    kde_matrix[is.na(kde_matrix)] <- 0
    write.csv(kde_matrix, paste0("./data/data_derived/table/",j,"_",i,
                          "_bur_II_spd_bin_kde.csv"))
    meta = data.frame(ndates=NA,nbins=NA,nsites=NA,nsim=kdesim,bw=kdebw)
    meta$ndates <- bkde_14C.spd.bins$metadata$ndates
    meta$nbins<- bkde_14C.spd.bins$metadata$nbins
    meta$nsites <- length(unique(bkde_14C$site_name))
    write.csv(meta, paste0("./data/data_derived/meta/meta_",j,"_",i,
                          "_bur_II_spd_bin_kde.csv"))
# ------------------------------------------------------------------------------
    not_bkde_14C <- subset(bur_14C, bur_14C[,j]!=i)
    not_bkde_14C.caldates=calibrate(x=not_bkde_14C$bp, errors=not_bkde_14C$std, 
                                calCurves='intcal20', method="standard", 
                                normalised=FALSE, ncores=ncores, calMatrix=TRUE)
    not_bkde_14C.bins = binPrep(sites=not_bkde_14C$site_name, 
                                ages=not_bkde_14C$bp, h=binh)
    not_bkde_14C.spd.bins = spd(not_bkde_14C.caldates, bins=not_bkde_14C.bins,
                                timeRange=c(workingstartBP,workingendBP),
                                datenormalised=FALSE, runm=runm)
    not_bkde_14C.randates = sampleDates(not_bkde_14C.caldates, 
                                        bins=not_bkde_14C.bins, nsim=kdesim,
                                        boot=TRUE)
    not_bkde_14C.ckde = ckde(not_bkde_14C.randates, 
                             timeRange=c(workingstartBP,workingendBP), bw=kdebw)
    kde_matrix = cbind(seq(workingstartBP,workingendBP),
                       not_bkde_14C.ckde$res.matrix) 
    kde_matrix[is.na(kde_matrix)] <- 0
    write.csv(kde_matrix, paste0("./data/data_derived/table/",j,"_not_",i,
                                 "_bur_II_spd_bin_kde.csv"))
    meta = data.frame(ndates=NA,nbins=NA,nsites=NA,nsim=kdesim,bw=kdebw)
    meta$ndates <- not_bkde_14C.spd.bins$metadata$ndates
    meta$nbins<- not_bkde_14C.spd.bins$metadata$nbins
    meta$nsites <- length(unique(not_bkde_14C$site_name))
    write.csv(meta, paste0("./data/data_derived/meta/meta_",j,"_not_",i,
                           "_bur_II_spd_bin_kde.csv"))
  }
}
```

### Settlement sites

Repeat procedure for a subset of settlement sites.

```
for (j in j_lst){
  region_lst = unique(set_14C[,j])
  for (i in region_lst){
    print(i)
    skde_14C <- subset(set_14C, set_14C[,j]==i)
    skde_14C.caldates=calibrate(x=skde_14C$bp, errors=skde_14C$std, 
                                calCurves='intcal20', method="standard", 
                                normalised=FALSE, ncores=ncores, calMatrix=TRUE)
    skde_14C.bins = binPrep(sites=skde_14C$site_name, ages=skde_14C$bp, h=binh)
    skde_14C.spd.bins = spd(skde_14C.caldates, bins=skde_14C.bins,
                            timeRange=c(workingstartBP,workingendBP),
                            datenormalised=FALSE, runm=runm)
    skde_14C.randates = sampleDates(skde_14C.caldates, bins=skde_14C.bins, 
                                    nsim=kdesim,boot=TRUE)
    skde_14C.ckde = ckde(skde_14C.randates, 
                         timeRange=c(workingstartBP,workingendBP), bw=kdebw)
    kde_matrix = cbind(seq(workingstartBP,workingendBP),
                       skde_14C.ckde$res.matrix) 
    kde_matrix[is.na(kde_matrix)] <- 0
    write.csv(kde_matrix, paste0("./data/data_derived/table/",j,"_",i,
                                "_set_II_spd_bin_kde.csv"))
    meta = data.frame(ndates=NA,nbins=NA,nsites=NA,nsim=kdesim,bw=kdebw)
    meta$ndates <- skde_14C.spd.bins$metadata$ndates
    meta$nbins<- skde_14C.spd.bins$metadata$nbins
    meta$nsites <- length(unique(skde_14C$site_name))
    write.csv(meta, paste0("./data/data_derived/meta/meta_",j,"_",i,
                          "_set_II_spd_bin_kde.csv"))
# ------------------------------------------------------------------------------
    not_skde_14C <- subset(set_14C, set_14C[,j]!=i)
    not_skde_14C.caldates=calibrate(x=not_skde_14C$bp, errors=not_skde_14C$std, 
                                    calCurves='intcal20', method="standard", 
                                    normalised=FALSE, ncores=ncores, 
                                    calMatrix=TRUE)
    not_skde_14C.bins = binPrep(sites=not_skde_14C$site_name, 
                                ages=not_skde_14C$bp, h=binh)
    not_skde_14C.spd.bins = spd(not_skde_14C.caldates, bins=not_skde_14C.bins,
                                timeRange=c(workingstartBP,workingendBP),
                                datenormalised=FALSE, runm=runm)
    not_skde_14C.randates = sampleDates(not_skde_14C.caldates, 
                                        bins=not_skde_14C.bins, nsim=kdesim,
                                        boot=TRUE)
    not_skde_14C.ckde = ckde(not_skde_14C.randates, 
                             timeRange=c(workingstartBP,workingendBP),bw=kdebw)
    kde_matrix = cbind(seq(workingstartBP,workingendBP),
                       not_skde_14C.ckde$res.matrix) 
    kde_matrix[is.na(kde_matrix)] <- 0
    write.csv(kde_matrix, paste0("./data/data_derived/table/",j,"_not_",i,
                                 "_set_II_spd_bin_kde.csv"))
    meta = data.frame(ndates=NA,nbins=NA,nsites=NA,nsim=kdesim,bw=kdebw)
    meta$ndates <- not_skde_14C.spd.bins$metadata$ndates
    meta$nbins<- not_skde_14C.spd.bins$metadata$nbins
    meta$nsites <- length(unique(not_skde_14C$site_name))
    write.csv(meta, paste0("./data/data_derived/meta/meta_",j,"_not_",i,
                           "_set_II_spd_bin_kde.csv"))
  }
}
```

## References

Crema ER. Statistical Inference of Prehistoric Demography from
Frequency Distributions of Radiocarbon Dates: A Review and a Guide for
the Perplexed. J Archaeol Method Theory. 2022;29(4):1387-1418. Available
from: https://doi.org/10.1007/s10816-022-09559-5

Crema ER, Bevan A. Inference from Large Sets of Radiocarbon Dates:
Software and Methods. Radiocarbon. 2021;63(1):23–39. Available from: http://doi.org/10.1017/RDC.2020.95

McLaughlin TR. On Applications of Space–Time Modelling with
Open-Source 14C Age Calibration. J Archaeol Method Theory.
2019;26(2):479–501.

Palmisano A, Bevan A, Kabelindde A, Roberts N, Shennan S. Long-Term
Demographic Trends in Prehistoric Italy: Climate Impacts and
Regionalised Socio-Ecological Trajectories. J World Prehist.
2021;34(3):381-432. Available from: https://doi.org/10.1007/s10963-021-09159-3

Parkinson EW, McLaughlin TR, Esposito C, Stoddart S, Malone C.
Radiocarbon Dated Trends and Central Mediterranean Prehistory. J World
Prehist. 2021;34(3):317-379. Available from: https://doi.org/10.1007/s10963-021-09158-4
