# Supplementary material for: Subsistence and population development from the Middle Neolithic B (2800–2350 BCE) to the Late Neolithic (2350–1700 BCE) in Southern Scandinavia
Source: PLoS One. 2024 Oct 28;19(10):e0301938. doi: 10.1371/journal.pone.0301938 (PMC11516014; doi:10.1371/journal.pone.0301938)
Supplement: S1 Supporting information — S1 File. SI_C01_SPD_KDE_models. R-script for analysing radiocarbon dates dates. The code performs the computation of over-regional and regional SPD and KDE models, as well as their export to CSV files (Rmd). S2 File. SI_C02_aoristic_dating. R-script for exporting aoristic time series derived from typochronological dated archaeological material as CSV files (Rmd). S3 File. SI_C03_vegetation_openness_score_example. R-script performing the computation of a vegetation openness score from pollen records and the export of the generated time series as CVS file (Rmd). S4 File. SI_C04_data_preparation. Jupyter Notebook performing the import and transformation of relevant data visualize plots exhibited in the paper (ipynb). S5 File. SI_C05_figures_extra. Jupyter Notebook visualizing the plots exhibited in the paper (ipynb). S1 Data. SI_D01_reg_data_no_dups. Spread sheet holding radiocarbon dates, with the information of laboratory identification, site name, geographical coordinates, site type, material, source and regional affiliation (csv). S2 Data. SI_D02_reg_axe_dagger_graves. Spread sheet holding entries of axes and daggers, with the information of context, site, parish, artefact identification, type, subtype, absolute dating, typochonological dating, references, geographical coordinates and regional affiliations (csv). S3 Data. SI_D03_pollen_example. Spread sheet holding sample entries of the pollen records from Krageholm (neotoma Site ID 3204) and Bjäresjöholmsjön (neotoma Site ID 3017) for example run of S3 File. Record can be access via the neotoma explorer (https://apps.neotomadb.org/explorer/) with their given IDs. Each entry holds the information of the records type, regional affiliation, absolute BP and BCE dating, as well as the counts of given plant taxa (csv). S4 Data. SI_D04_PAP_303600_TOC_LOI. Table holding sample entries of TOC content, LOI and SST reconstruction of sediment core PAP_303600 for correlations of population development with Baltic sea surface t [file pone.0301938.s001.zip › support_information/SI_C02_aoristic_dating.html]

Aoristic dating of MNB battle axes and LN flint daggers


# Aoristic dating of MNB battle axes and LN flint daggers

#### Julian Laabs

*“Aoristic analysis can provide a temporal weight and give an
indication of the probability that the event occurred within a defined
period.”* (Ratcliff 2000)

For archaeology aoristic dating is a way to turn typochronologiocal
stages, that are absolute chronologically “anchored”, into probability
density distribution on the time axis (Johnson 2004, Mischka 2007). Such
probability density distributions for sites, artefacts, etc. can be
summed and similarly used as summed radiocarbon probability densities
(e.g. Crema and Bevan 2021).

This SI belongs to the study **“Subsistence and Population
developments from the late Middle to the Late Neolithic in Southern
Scandinavia”** (DOI: **tba**). Please consider the
publication for context and content related information.

The script performs the computation of aoristic time series from
archaeologically dated materia culture.

## Preparation

Load (or install) the R-package `aoristAAR` (Schmid et
al. 2017) to perform aoristic analysis and/or reproduce the aoristic
time series used in the aforementioned study.

```
#if(!require('devtools')) install.packages('devtools')
#library(devtools)
#install_github('ISAAKiel/aoristAAR')
library(aoristAAR)
```

The original data needs to be imported and slightly manipulated.
Latter process means the transformation of the start and end dates,
which are given in BCE (positive numbers), into CE dates (negative
numbers if dating before Common Era). Further, data that cannot
attributed to burial contexts is exempt from analysis.

```
# Load data
df <- read.csv("./data/data_raw/reg_axe_dagger_graves.csv", header=TRUE)

# Turn dating columns from BCE to CE (needed for aoristAAR)
df$dating_end <- df$dating_end-1

# Delete find from context that are unknown
df <- subset(df, df[,"context_name"]!="Single_Find")
df <- subset(df, df[,"context_name"]!="unknown")
```

Although our time window of interest is focused on MNB to LN
(2850-1700 BCE), we nee to take the dating span of the artefacts into
account. In our case flint dagger date also after LN into the Bronze Age
until c. 1100 BCE. To take this into account, we need to compute the
full length time series to not artificially increase density values of
daggers at the end of the LN.

```
# Find start and end date of the data set
dat_start <- min(df[,"dating_start"])
dat_end <- max(df[,"dating_end"])
```

## Exploring

Before producing aoristic time series for the regioanlized analysis.
Explore the differences of the methods “number”, “weight” and
“period\_correction” available for the creation of aoristic density
distributions. See `help(aorist)` for further insights to the
methods.

```
# Subset df into an axe and dagger df
aoAXE <- subset(df, df[,"type"]=="axe")
aoDAG <- subset(df, df[,"type"]=="dagger")

# Create df list for iteration
df_lst <- list(aoAXE,aoDAG)

# Create df for holding aoristic time series 
dfAO_all <- data.frame(CE = seq(dat_start,dat_end))

# Iterate over the methods of function aorist() "number", "weight" and "period_correction"
for (m in c("number","weight","period_correction")){
  # Iterate over the df's in df_lst
  for(x in seq(1,length(df_lst))){
    # calculate density of occurrences
    aoristic_time_series <- aorist(df_lst[[x]],
                                   from = "dating_start", to = "dating_end",
                                   method = m)
    
    # Find start and end date of the data set
    start_date <- min(aoristic_time_series$date)
    end_date <- max(aoristic_time_series$date)
    
    # Find index of the data set's start and end date
    idx_start <- which(dfAO_all[,"CE"] == min(aoristic_time_series$date))
    idx_end <- which(dfAO_all[,"CE"] == max(aoristic_time_series$date))
    
    # Conditional statement deciding if df is aoAXE or aoDAG
    if (x == 1){
      dfAO_all[,"axe_all"] <- 0
      dfAO_all[idx_start:idx_end,"axe_all"] <- aoristic_time_series$sum
    }else{
      dfAO_all[,"dagger_all"] <- 0
      dfAO_all[idx_start:idx_end,"dagger_all"] <- aoristic_time_series$sum
    }
  }
  # Find max value of y axis
  maxy = pmax(max(dfAO_all[,"dagger_all"]), max(dfAO_all[,"axe_all"]))
  
  # Plot it
  plot(dfAO_all[,"CE"], dfAO_all[,"dagger_all"], 
       type = "l", col = "red", lty = 2, 
       ylab="aoristic values", ylim =c(0,maxy),
       xlab="CE")
  lines(dfAO_all[,"CE"], dfAO_all[,"axe_all"], 
        type = "l", col = "blue")
  legend(-1400, maxy*0.99, legend = c("axe", "dagger"),
         col = c("blue", "red"), lty = 1:2, cex = 0.8)
  title(m)
}
```

In regard to the methods offered by aoristARR, “period\_correction”
has one advantage:

*“The calculation of the aoristic sum is based on exclusive time
intervals in its original implementation (Radcliffe 2000). In
archaeological applications, however, overlapping time intervals often
result from different dating accuracy. For example, individual sites may
only be categorized as part of the Neolithic, others may be narrowed
down to the Middle Neolithic Ia. The structure of the overlapping time
intervals can lead to biases of the aoristic sum (Hinz/Müller-Scheeßel
forthcoming), which is corrected by the algorithm by weighting down
multiple time periods.”* (Schmid et al. 2017)

The overlapping of time intervals in our data set is limited,
however, it seems appropriate to apply the method “period\_correction”
anyways.

## Regionalized analysis

The procedure is now repeated for each defined region and the
aoristic time series are saved as .csv file for each of them for later
use.

```
# Use df with period corrected aoristic time series of axes and daggers for the full study region
dfAO = dfAO_all

# Create meta data df for storing information on item counts per region
dfMETA <- data.frame()
dfMETA["all","n_axe"] <- length(aoAXE$type)
dfMETA["all","n_dagger"] <- length(aoDAG$type)

# Define columns holding regional affiliation on different scales
j_lst = c("region_geom_2")

# Iterate over all given regional affiliation columns
for (j in j_lst){
  # List unique regions in region column
  region_lst = unique(df[,j])
  
  # Delete Bornholm entry from list
  region_lst = region_lst[!region_lst == "Scania and Bornholm"]
  
  # Iterate over regions
  for (i in seq(1,length(region_lst))){
    # Subset whole data set to current region
    ao <- subset(df, df[,j]==region_lst[i])
    
    # Subset regional data set to an axe and dagger df
    aoAXE <- subset(ao, ao[,"type"]=="axe")
    aoDAG <- subset(ao, ao[,"type"]=="dagger")
    
    # Save counts of artefacts in meta data df
    dfMETA[region_lst[i],"n_axe"] <- length(aoAXE$type)
    dfMETA[region_lst[i],"n_dagger"] <- length(aoDAG$type)
    
    # Create df list for iteration
    df_lst <- list(aoAXE,aoDAG)
    
    # Iterate over axe and dagger df subsets
    for(x in seq(1,length(df_lst))){
      # Calculate period corrected density of occurrences
      aoristic_time_series <- aorist(df_lst[[x]],
                                     from = "dating_start",to = "dating_end",
                                     method = "period_correction")
      
      # Find index of the data set's start and end date
      idx_start <- which(dfAO[,"CE"] == min(aoristic_time_series$date))
      idx_end <- which(dfAO[,"CE"] == max(aoristic_time_series$date))
      
      # Conditional statement deciding if df is aoAXE or aoDAG
      if (x == 1){
        dfAO[,paste0("axe_",region_lst[i])] <- 0
        dfAO[idx_start:idx_end,paste0("axe_",region_lst[i])] <-
          aoristic_time_series$sum
      }else{
        dfAO[,paste0("dagger_",region_lst[i])] <- 0
        dfAO[idx_start:idx_end,paste0("dagger_",region_lst[i])] <-
          aoristic_time_series$sum
      }
    }
  }
  # Write aoristic time series to .csv file
  write.csv(dfAO, paste0("./data/data_derived/table/",j,"_ao_da.csv"))
  write.csv(dfMETA, paste0("./data/data_derived/meta/meta_",j,"_ao_da.csv"))
}
```

### Plotting regionalized results

```
# Iterate over regions
for (i in seq(1,length(region_lst))){
  # Find max value of y axis
  maxy = pmax(max(dfAO[,paste0("dagger_",region_lst[i])]), 
              max(dfAO[,paste0("axe_",region_lst[i])]))
  
  # Plot it 
  plot(dfAO[,"CE"], dfAO[,paste0("dagger_",region_lst[i])], 
       type = "l", col = "red", lty = 2,
       xlim = c(-2850, -1700), xlab="CE",
       ylim = c(0, maxy), ylab="aoristic values")
  lines(dfAO[,"CE"], dfAO[,paste0("axe_",region_lst[i])], 
        type = "l", col = "blue")
  legend(-1900, maxy*0.99, 
    legend = c("axe", "dagger"),
    col = c("blue", "red"), lty = 1:2, cex = 0.8)
  title(region_lst[i])
}
```

## References

Crema ER, Bevan A. Inference from Large Sets of Radiocarbon Dates:
Software and Methods. Radiocarbon. 2021 Feb;63(1):23–39. Available from:
http://doi.org/10.1017/RDC.2020.95

Johnson I. Aoristic Analysis: Seeds of a New Approach to Mapping
Archaeological Distributions through Time. In: Fischer-Ausserer K,
Börner W, Goriany W, editors. Enter the Past The E-way into the four
Dimensions of Cultural Heritage [Internet]. Oxford: Archaeopress; 2004.
p. 448–52. (BAR International Series). Available from: http://dx.doi.org/10.15496/publikation-2085

Mischka D. Methodische Aspekte zur Rekonstruktion prähistorischer
Siedlungsmuster. Landschaftsgenese vom Ende des Neolithikums bis zur
Eisenzeit im Gebiet des südlichen Oberrheins. Rahden/Westf.: Marie
Leidorf; 2007. (Freiburger Archäologische Studien).

Ratcliffe JH. Aoristic analysis: the spatial interpretation of
unspecific temporal events. International Journal of Geographical
Information Science. 2000 Oct 1;14(7):669–79. Available from: http://doi.org/10.1080/136588100424963

Schmid C, Hinz M, Müller-Scheeßel N, Raese H. aoristAAR [Internet].
Initiative for Statistical Analysis in Archaeology Kiel (ISAAK); 2017.
Available from: https://github.com/ISAAKiel/aoristAAR
