# Supplementary material for: Subsistence and population development from the Middle Neolithic B (2800–2350 BCE) to the Late Neolithic (2350–1700 BCE) in Southern Scandinavia
Source: PLoS One. 2024 Oct 28;19(10):e0301938. doi: 10.1371/journal.pone.0301938 (PMC11516014; doi:10.1371/journal.pone.0301938)
Supplement: S1 Supporting information — S1 File. SI_C01_SPD_KDE_models. R-script for analysing radiocarbon dates dates. The code performs the computation of over-regional and regional SPD and KDE models, as well as their export to CSV files (Rmd). S2 File. SI_C02_aoristic_dating. R-script for exporting aoristic time series derived from typochronological dated archaeological material as CSV files (Rmd). S3 File. SI_C03_vegetation_openness_score_example. R-script performing the computation of a vegetation openness score from pollen records and the export of the generated time series as CVS file (Rmd). S4 File. SI_C04_data_preparation. Jupyter Notebook performing the import and transformation of relevant data visualize plots exhibited in the paper (ipynb). S5 File. SI_C05_figures_extra. Jupyter Notebook visualizing the plots exhibited in the paper (ipynb). S1 Data. SI_D01_reg_data_no_dups. Spread sheet holding radiocarbon dates, with the information of laboratory identification, site name, geographical coordinates, site type, material, source and regional affiliation (csv). S2 Data. SI_D02_reg_axe_dagger_graves. Spread sheet holding entries of axes and daggers, with the information of context, site, parish, artefact identification, type, subtype, absolute dating, typochonological dating, references, geographical coordinates and regional affiliations (csv). S3 Data. SI_D03_pollen_example. Spread sheet holding sample entries of the pollen records from Krageholm (neotoma Site ID 3204) and Bjäresjöholmsjön (neotoma Site ID 3017) for example run of S3 File. Record can be access via the neotoma explorer (https://apps.neotomadb.org/explorer/) with their given IDs. Each entry holds the information of the records type, regional affiliation, absolute BP and BCE dating, as well as the counts of given plant taxa (csv). S4 Data. SI_D04_PAP_303600_TOC_LOI. Table holding sample entries of TOC content, LOI and SST reconstruction of sediment core PAP_303600 for correlations of population development with Baltic sea surface t [file pone.0301938.s001.zip › support_information/SI_C03_vegetation_openness_score_example.html]

Vegetation openness scores (Example)


# Vegetation openness scores (Example)

#### Julian Laabs

This Supplementary Information belongs to the study
**“Subsistence and Population developments from the late Middle to
the Late Neolithic in Southern Scandinavia”** (DOI:
**tba**). Please consider the publication for context and
content related information.

The code has been adopted and modified from the Supplementary
Information from the preprint of Hinz et al. (2022) **“Bayesian
inference of prehistoric population dynamics from multiple proxies: a
case study from the North of the Swiss Alps”** (DOI: https://osf.io/preprints/socarxiv/dbcag/).

The script performs the computation of a vegetation openness score
from pollen records. This code is only an example to show the
application of the method. Most of the data used in the above study are
not yet published and cannot used as data sets for reproduction in the
scope of this work. Due to this circumstance we use pollen data from
Southern Sweden downloaded from the Neotoma Paleoecology Database (https://apps.neotomadb.org/explorer/).

In the case of this example the separation does not work well, as the
records only share few open vegetation pollen taxa related to
differentiate from forest pollen taxa. **Therefore, the resulting
time series cannot be read as vegetation openness score!**

## Preparation

Load (or install) neccessary R-packages to perform aoristic analysis
and/or reproduce the aoristic time series used in the aforementioned
study.

```
# Load packages
library(tidyverse)
library(vegan)
library(ggplot2)
library(reshape2)
library(here)
library(RcppRoll)
```

Load data. The data has been compiled from different data sets and
was unified to a set of relevant plant taxa.

```
data <- read.csv("./data/data_raw/SI_D03_pollen_example.csv", header=TRUE, 
                 stringsAsFactors=FALSE, encoding="UTF-8", dec=",",
                 na.strings=c("NA",""), strip.white=TRUE)
```

Define smoothing factor and the time frame for which openness is
computed. Although our focus lies on 2850-1700 BCE, Feeser et al. (2019)
suggest to compute openness from 4800-700 BCE as this we need to see the
development of human impact in context of a longer lasting
trajectory.

```
sw = 50 #smothing time window
start_date = -4800
end_date = -700
step_date = 100
timeframe <- c(start_date,end_date)
```

## Example from Southern Sweden

Subset relevant data for needed records, if needed. For the example
not needed

```
## Subset pollen records
#data1 <- subset(data, site == "Krageholm" | site == "Ageröds Mosse")
data1 <- data
```

Calculate proportions form pollen counts.

```
# Calculate proportions for count data set
data1$sums = rowSums(data1[,7:ncol(data1)])
Pdata <- data1[,1:ncol(data1)-1]
for (i in seq(from=7,to=ncol(data1)-1))
{
  Pdata[,i] = data1[,i]/data1[,"sums"]
}
```

Data homogenization. Subset relevant time frame (4800-700 BCE). Drop
taxa with very low percentages or which are present in less of 1/3 of
the pollen records.

```
Pdata_cut <- subset(Pdata, CE > timeframe[1] & CE < timeframe[2])
Pdata_all <- Pdata_cut
Pdata_cut <- Pdata_cut[,7:ncol(Pdata_cut)]
Pdata_cut <- Pdata_cut[,colSums(Pdata_cut) != 0]
Pdata_cut <- Pdata_cut[,colSums(Pdata_cut>0)>(nrow(Pdata_cut)/3)]
Pdata_x <- Pdata_cut[,colMeans(Pdata_cut)>0.01]
```

If no *cerealia* is present in a record add a
*cerealia* column with 0%. This is because *cerealia* will
be treated as “the” cultural marker.

```
if((!("Cerealia" %in% colnames(Pdata_x))) & (sum(Pdata_all[,"Cerealia"]) > 0.0))
{
  Pdata_x <- data.frame(Pdata_x , Cerealia = Pdata_all[,"Cerealia"])  
}
```

Normalize the columns (z-scores).

```
# Scale columns
for (i in seq(from=1,to=ncol(Pdata_x)))
{
  if(sum(Pdata_x[,i]) > 0)
  {
    Pdata_x[,i] = scale(Pdata_x[,i])
  }
}
```

Compute PCA. If *fagus* is present, its influence will be
eliminated by using a partial RDA. This is due to the increase of
*fagus* over the Holocene and establishment as the dominant tree
(Feeser et al. 2019). A PCA involving it in many cases will measure this
gradient, which also coincides in most cases with increasing human
impact and open land indicators.

```
if(("Fagus" %in% colnames(Pdata_x)))
{
   res.pca <- rda(Pdata_x ~ Condition(Fagus), data=Pdata_x, scale = FALSE)
   biplot(res.pca, type="text")
  }else{
   res.pca <- rda(Pdata_x, scale = FALSE)
   biplot(res.pca, type="text")
}
```

In general the data sets separate quite well into open and wood land
taxa in our PCA, although *pinus* often affiliates with openness.
In the case of this example the separation does not work well, as the
records only share few pollen taxa related to open vegetation to
differentiate from forest taxa.

Plot the PCA results as time series for each of the single records
from 4800-700 BCE.

```
orientation <- sign(scores(res.pca)$species["Cerealia",1])
res.open <- data.frame(scores(res.pca, choices = 1, display = "si")*orientation)
colnames(res.open) <- "score"
res.open$CE <- Pdata_all$CE
res.open$site <- Pdata_all$site
g <- ggplot(res.open) + geom_line(aes(x = CE, y = score, color = site)) +
  scale_x_continuous(breaks=seq(start_date,end_date,step_date)) +
  labs(y = "Openness score [PCA]",x = "CE",colour = "Sites") +
  theme_minimal() +
  theme(axis.text.x = element_text(angle = 45, hjust = 1))
# Plot it
g
```

Prepare a 50 year smoothed time series of each record.

```
full_range <- full_seq(round(res.open$CE,0), 1)
res.open.interpolated <- by(res.open, res.open$site, 
                            function(x) approx(x$CE,x$score, full_range)$y)
res.open.interpolated <- data.frame(do.call(cbind, res.open.interpolated))
res.open.interpolated$CE <- full_range
res.open.interpolated <- melt(res.open.interpolated, id.vars=c("CE"))
res.open.interpolated$site <- res.open.interpolated$variable
res.open.interpolated$variable <- NULL
res.open_smoothed <- res.open.interpolated %>% 
  group_by(site) %>%
  # RcppRoll::roll_mean() is written in C++ for speed 
  mutate(moving_mean = roll_mean(value, sw, fill = NA, na.rm = TRUE))
res.open_smoothed$value<-NULL
res.open_smoothed <- na.omit(res.open_smoothed)
res.open_smoothed <- res.open_smoothed[res.open_smoothed$CE %in%
                                         seq(from=start_date,
                                             to=end_date, by=50),]
```

Plot single records’ openness scores, and a mean of all records with
uncertainty envelope in 50 years steps.

```
pp <- ggplot(res.open_smoothed,aes(x=CE,y=moving_mean)) +
  geom_point(aes(color=site), alpha =.5) +
  geom_smooth(span = 0.05, color = "black") +
  scale_x_continuous(breaks=seq(start_date,end_date,step_date)) +
  labs(y = "Openness score [PCA]",x = "CE",colour = "Sites") +
  theme_minimal() +
  theme(axis.text.x = element_text(angle = 45, hjust = 1))
# Plot it
pp
```

Export and save the time series to .csv file for later use.

```
## Save data
#ppp <- ggplot_build(pp)$data[[2]]
#write.csv(ppp[,1:5],"./data/data_derived/table/vos_example.csv")
```

Plot mean of all records with min-max span of the records values in
50 years steps.

```
res.open_final <- res.open_smoothed %>%
  group_by(CE) %>%
  mutate(mean=mean(moving_mean),
         sd = sd(moving_mean)) %>% 
  select(CE,mean,sd) %>%
  unique()  %>% arrange(desc(CE))
# Plot it
ggplot(res.open_final,aes(x=CE)) +
  geom_line(aes(y=mean)) +
  geom_segment(aes(y=mean-sd, yend =mean+sd, xend=CE), alpha = .25)
```

## References

Feeser I, Dörfler W, Kneisel J, Hinz M, Dreibrodt S. Human impact and
population dynamics in the Neolithic and Bronze Age: Multi-proxy
evidence from north-western Central Europe. The Holocene. 2019;
29(10):1596–606. Available from: https://doi.org/10.1177/0959683619857223

Hinz M, Roe J, Laabs J, Heitz C, Kolář J. Bayesian inference of
prehistoric population dynamics from multiple proxies: a case study from
the North of the Swiss Alps. SocArXiv. 2022. Available from: https://osf.io/preprints/socarxiv/dbcag/
