# Supplementary material for: Subsistence and population development from the Middle Neolithic B (2800–2350 BCE) to the Late Neolithic (2350–1700 BCE) in Southern Scandinavia
Source: PLoS One. 2024 Oct 28;19(10):e0301938. doi: 10.1371/journal.pone.0301938 (PMC11516014; doi:10.1371/journal.pone.0301938)
Supplement: S1 Supporting information — S1 File. SI_C01_SPD_KDE_models. R-script for analysing radiocarbon dates dates. The code performs the computation of over-regional and regional SPD and KDE models, as well as their export to CSV files (Rmd). S2 File. SI_C02_aoristic_dating. R-script for exporting aoristic time series derived from typochronological dated archaeological material as CSV files (Rmd). S3 File. SI_C03_vegetation_openness_score_example. R-script performing the computation of a vegetation openness score from pollen records and the export of the generated time series as CVS file (Rmd). S4 File. SI_C04_data_preparation. Jupyter Notebook performing the import and transformation of relevant data visualize plots exhibited in the paper (ipynb). S5 File. SI_C05_figures_extra. Jupyter Notebook visualizing the plots exhibited in the paper (ipynb). S1 Data. SI_D01_reg_data_no_dups. Spread sheet holding radiocarbon dates, with the information of laboratory identification, site name, geographical coordinates, site type, material, source and regional affiliation (csv). S2 Data. SI_D02_reg_axe_dagger_graves. Spread sheet holding entries of axes and daggers, with the information of context, site, parish, artefact identification, type, subtype, absolute dating, typochonological dating, references, geographical coordinates and regional affiliations (csv). S3 Data. SI_D03_pollen_example. Spread sheet holding sample entries of the pollen records from Krageholm (neotoma Site ID 3204) and Bjäresjöholmsjön (neotoma Site ID 3017) for example run of S3 File. Record can be access via the neotoma explorer (https://apps.neotomadb.org/explorer/) with their given IDs. Each entry holds the information of the records type, regional affiliation, absolute BP and BCE dating, as well as the counts of given plant taxa (csv). S4 Data. SI_D04_PAP_303600_TOC_LOI. Table holding sample entries of TOC content, LOI and SST reconstruction of sediment core PAP_303600 for correlations of population development with Baltic sea surface t [file pone.0301938.s001.zip › support_information/SI_C04_data_preparation.html]

SI\_C04\_data\_preparation


# Data preparation - super df¶

## Preparation¶

In [1]:

```
# Load packages
import sys, os
import numpy as np
import pandas as pd
import matplotlib.pyplot as plt

from scipy import signal
```

In [2]:

```
import warnings
warnings.filterwarnings('ignore')
```

In [3]:

```
sys.path.insert(0, './functions')
from functions import SI_C06_func_multi_plotting as mp

inp_dir = "./data/data_raw/"
out_dir = "./data/data_derived/"
```

In [4]:

```
# Create list objects from files in the respective folder
meta_file_lst = os.listdir(out_dir+"meta/")
file_lst = os.listdir(out_dir+"table/")
## Inspect list object
#print(file_lst[0:4])
```

In [5]:

```
# Dating start and end in CE
dat_start = -2850
dat_end = -1699

# Create df 
df = pd.DataFrame()
df["CE"] = np.arange(dat_start-100, dat_end+100)
df["BP"] = (df["CE"] - 1950) * -1
```

## Add KDE models (geomorphological regions)¶

In [6]:

```
# Write KDE model (all sites, all regions) into main df
for i in ["all","set","bur"]:
    # Load data
    kde = pd.read_csv(out_dir+"table/all_"+i+"_spd_bin_kde.csv")
    
    # Define BP column and transform to CE
    kde["calBP"] = kde["V1"]
    kde["calCE"] = (kde["calBP"] - 1950) * -1
    
    # Calculate mean and standard deviation for each year
    kde.loc[::,"mean"] = kde.loc[::,"V2":"V500"].mean(axis=1)
    kde.loc[::,"std"] = kde.loc[::,"V2":"V500"].std(axis=1)
    
    # Write KDE model into main df
    df["kde_"+i+"_mean_all"] = kde.loc[::,"mean"].values
    df["kde_"+i+"_std_all"] = kde.loc[::,"std"].values
    df["kde_"+i+"_detrend_all"] = signal.detrend(kde.loc[::,"mean"].values)
    df["kde_"+i+"_growth_all"] = kde.loc[::,"mean"].pct_change(periods=10).values
```

In [7]:

```
# All site types data
w1, w2 = "all_spd_bin_kde", "region_geom_2"
remove_word = ["North Eastern Germany", "Western Sweden"]
meta_lst, r_name = mp.meta(meta_file_lst, w1, w2, remove_word, out_dir)
kde_lst, not_kde_lst, r_name = mp.kde_lst(file_lst, w1, w2, remove_word, out_dir)

# Settlement data
w1, w2 = "set_II_spd_bin_kde", "region_geom_2"
remove_word = ["North Eastern Germany", "Western Sweden"]
s_meta_lst, s_r_name = mp.meta(meta_file_lst, w1, w2, remove_word, out_dir)
s_kde_lst, s_not_kde_lst, s_r_name = mp.kde_lst(file_lst, w1, w2, remove_word, out_dir)

# Burial data
w1, w2 = "bur_II_spd_bin_kde", "region_geom_2"
remove_word = ["North Eastern Germany", "Western Sweden"]
b_meta_lst, b_r_name = mp.meta(meta_file_lst, w1, w2, remove_word, out_dir)
b_kde_lst, b_not_kde_lst, b_r_name = mp.kde_lst(file_lst, w1, w2, remove_word, out_dir)
```

In [8]:

```
for i in np.arange(0,len(kde_lst)):
    # All site types data
    df["kde_all_mean_"+r_name[i]] = kde_lst[i].loc[::,"mean"].values
    df["kde_all_detrend_"+r_name[i]] = signal.detrend(kde_lst[i].loc[::,"mean"].values)
    df["kde_all_growth_"+r_name[i]] = kde_lst[i].loc[::,"mean"].pct_change(periods=10).values
    df["kde_all_mean_not_"+r_name[i]] = not_kde_lst[i].loc[::,"mean"].values
    df["kde_all_std_"+r_name[i]] = kde_lst[i].loc[::,"std"].values
    df["kde_all_std_not_"+r_name[i]] = not_kde_lst[i].loc[::,"std"].values

    # Settlement data
    df["kde_set_mean_"+r_name[i]] = s_kde_lst[i].loc[::,"mean"].values
    df["kde_set_detrend_"+r_name[i]] = signal.detrend(s_kde_lst[i].loc[::,"mean"].values)
    df["kde_set_growth_"+r_name[i]] = s_kde_lst[i].loc[::,"mean"].pct_change(periods=10).values
    df["kde_set_mean_not_"+r_name[i]] = s_not_kde_lst[i].loc[::,"mean"].values
    df["kde_set_std_"+r_name[i]] = s_kde_lst[i].loc[::,"std"].values
    df["kde_set_std_not_"+r_name[i]] = s_not_kde_lst[i].loc[::,"std"].values
    
    # Burial data
    df["kde_bur_mean_"+r_name[i]] = b_kde_lst[i].loc[::,"mean"].values
    df["kde_bur_detrend_"+r_name[i]] = signal.detrend(b_kde_lst[i].loc[::,"mean"].values)
    df["kde_bur_growth_"+r_name[i]] = b_kde_lst[i].loc[::,"mean"].pct_change(periods=10).values
    df["kde_bur_mean_not_"+r_name[i]] = b_not_kde_lst[i].loc[::,"mean"].values
    df["kde_bur_std_"+r_name[i]] = b_kde_lst[i].loc[::,"std"].values
    df["kde_bur_std_not_"+r_name[i]] = b_not_kde_lst[i].loc[::,"std"].values
```

## Add KDE models (pollen reference regions)¶

In [9]:

```
# All site types data
w1, w2 = "all_spd_bin_kde", "region_geom_3"
remove_word = ["npr"]
kde_lst_3, not_kde_lst_3, r_name_3 = mp.kde_lst(file_lst, w1, w2, remove_word, out_dir)

# Settlement data
w1, w2 = "set_II_spd_bin_kde", "region_geom_3"
remove_word = ["npr"]
s_kde_lst_3, s_not_kde_lst_3, s_r_name_3 = mp.kde_lst(file_lst, w1, w2, remove_word, out_dir)

# Burial data
w1, w2 = "bur_II_spd_bin_kde", "region_geom_3"
remove_word = ["npr"]
b_kde_lst_3, b_not_kde_lst_3, b_r_name_3 = mp.kde_lst(file_lst, w1, w2, remove_word, out_dir)
```

In [10]:

```
for i in np.arange(0,len(kde_lst_3)):
    # All site types data
    df["kde_all_mean_"+r_name_3[i]] = kde_lst_3[i].loc[::,"mean"].values
    df["kde_all_detrend_"+r_name_3[i]] = signal.detrend(kde_lst_3[i].loc[::,"mean"].values)
    df["kde_all_growth_"+r_name_3[i]] = kde_lst_3[i].loc[::,"mean"].pct_change(periods=10).values
    df["kde_all_mean_not_"+r_name_3[i]] = not_kde_lst_3[i].loc[::,"mean"].values
    df["kde_all_std_"+r_name_3[i]] = kde_lst_3[i].loc[::,"std"].values
    df["kde_all_std_not_"+r_name_3[i]] = not_kde_lst_3[i].loc[::,"std"].values
    
    # Settlement data
    df["kde_set_mean_"+r_name_3[i]] = s_kde_lst_3[i].loc[::,"mean"].values
    df["kde_set_detrend_"+r_name_3[i]] = signal.detrend(s_kde_lst_3[i].loc[::,"mean"].values)
    df["kde_set_growth_"+r_name_3[i]] = s_kde_lst_3[i].loc[::,"mean"].pct_change(periods=10).values
    df["kde_set_mean_not_"+r_name_3[i]] = s_not_kde_lst_3[i].loc[::,"mean"].values
    df["kde_set_std_"+r_name_3[i]] = s_kde_lst_3[i].loc[::,"std"].values
    df["kde_set_std_not_"+r_name_3[i]] = s_not_kde_lst_3[i].loc[::,"std"].values
    
    # Burial data
    df["kde_bur_mean_"+r_name_3[i]] = b_kde_lst_3[i].loc[::,"mean"].values
    df["kde_bur_detrend_"+r_name_3[i]] = signal.detrend(b_kde_lst_3[i].loc[::,"mean"].values)
    df["kde_bur_growth_"+r_name_3[i]] = b_kde_lst_3[i].loc[::,"mean"].pct_change(periods=10).values
    df["kde_bur_mean_not_"+r_name_3[i]] = b_not_kde_lst_3[i].loc[::,"mean"].values
    df["kde_bur_std_"+r_name_3[i]] = b_kde_lst_3[i].loc[::,"std"].values
    df["kde_bur_std_not_"+r_name_3[i]] = b_not_kde_lst_3[i].loc[::,"std"].values
```

## Add VOS models (pollen reference regions)¶

### Multiple pollen records¶

In [11]:

```
file_name_lst = ["north","zealand"]
regi_name_lst = ["Northern Jutland", "Zealand"]

for i in np.arange(0,len(file_name_lst)):
    dfP = pd.read_csv(inp_dir+"/SI_D05_vos_"+file_name_lst[i]+".csv")
    dfP.loc[::,"CE"] = np.round(dfP.loc[::,"x"],0)
    
    # Create transfer df
    dfT = pd.DataFrame(columns=["CE"])
    dfT["CE"] = np.arange(dfP["CE"].min(),dfP["CE"].max()+1)
    maskPN = dfT['CE'].isin(dfP.loc[::,"CE"].tolist())
    dfT.loc[maskPN,"VOS_mean"] = dfP.loc[::,"y"].values
    dfT.loc[maskPN,"VOS_std"] = dfP.loc[::,"se"].values
    dfT['VOS_mean_inter'] = dfT['VOS_mean'].interpolate(method='linear')
    dfT['VOS_std_inter'] = dfT['VOS_std'].interpolate(method='linear')
    # As there are negative score the scores must be made positive to calculate the rate of change
    dfT['VOS_growth'] = (dfT['VOS_mean_inter'] + abs(dfT['VOS_mean_inter'].min())+1).pct_change(50).interpolate(method="cubic").values

    # Transfer data to df
    maskDAT = (dfT.loc[::,"CE"] >= df["CE"].min()) & (dfT.loc[::,"CE"] <= df["CE"].max())
    df.loc[::,"VOS_mean_"+regi_name_lst[i]] = dfT.loc[maskDAT,'VOS_mean'].values
    df.loc[::,"VOS_std_"+regi_name_lst[i]] = dfT.loc[maskDAT,'VOS_std'].values
    df.loc[::,"VOS_mean_"+regi_name_lst[i]+"_inter"] = dfT.loc[maskDAT,'VOS_mean_inter'].values
    df.loc[::,"VOS_std_"+regi_name_lst[i]+"_inter"] = dfT.loc[maskDAT,'VOS_std_inter'].values
    df.loc[::,"VOS_mean_"+regi_name_lst[i]+"_detrend"] = signal.detrend(dfT.loc[maskDAT,'VOS_mean_inter'].values)
    df.loc[::,"VOS_mean_"+regi_name_lst[i]+"_growth"] = dfT.loc[maskDAT,'VOS_growth'].values

    ## Plot it
    #plt.plot(df['CE'], df["VOS_mean_"+regi_name_lst[i]+"_inter"], color = "black")
    #plt.scatter(df['CE'], df["VOS_mean_"+regi_name_lst[i]], color = "black")
    #plt.fill_between(df['CE'],
    #                 df["VOS_mean_"+regi_name_lst[i]+"_inter"] - df["VOS_std_"+regi_name_lst[i]+"_inter"],
    #                df["VOS_mean_"+regi_name_lst[i]+"_inter"] + df["VOS_std_"+regi_name_lst[i]+"_inter"],
    #                 color="grey", alpha=0.15)
    #plt.title("VOS "+regi_name_lst[i])
    #plt.show()
```

### Single pollen records¶

In [12]:

```
file_name_lst = ["vinge","belau"]
regi_name_lst = ["Vinge", "Southern Jutland"]

for i in np.arange(0,len(file_name_lst)):
    dfP = pd.read_csv(inp_dir+"/SI_D05_vos_"+file_name_lst[i]+".csv")
    dfP.loc[::,"CE"] = np.round(dfP.loc[::,"CE"],0)
    
    # Create transfer df
    dfT = pd.DataFrame(columns=["CE"])
    dfT["CE"] = np.arange(dfP["CE"].min(),dfP["CE"].max()+1)
    maskPN = dfT['CE'].isin(dfP.loc[::,"CE"].tolist())
    dfT.loc[maskPN,"VOS_mean"] = dfP.loc[::,"score"].values
    dfT['VOS_mean_inter'] = dfT['VOS_mean'].interpolate(method='linear')
    
    # As there are negative score the scores must be made positive to calculate the rate of change
    dfT['VOS_growth'] = (dfT['VOS_mean_inter'] + \
          abs(dfT['VOS_mean_inter'].min())+1).pct_change(50).interpolate(method="cubic").values
    
    # Transfer data to df
    maskDAT = (dfT.loc[::,"CE"] >= df["CE"].min()) & (dfT.loc[::,"CE"] <= df["CE"].max())
    df.loc[::,"VOS_mean_"+regi_name_lst[i]] = dfT.loc[maskDAT,'VOS_mean'].values
    df.loc[::,"VOS_mean_"+regi_name_lst[i]+"_inter"] = dfT.loc[maskDAT,'VOS_mean_inter'].values
    df.loc[::,"VOS_mean_"+regi_name_lst[i]+"_detrend"] = signal.detrend(dfT.loc[maskDAT,'VOS_mean_inter'].values)
    df.loc[::,"VOS_mean_"+regi_name_lst[i]+"_growth"] = dfT.loc[maskDAT,'VOS_growth'].values
    
    ## Plot it
    #plt.plot(df['CE'], df["VOS_mean_"+regi_name_lst[i]+"_inter"], color = "black")
    #plt.scatter(df['CE'], df["VOS_mean_"+regi_name_lst[i]], color = "black")
    #plt.title("VOS "+regi_name_lst[i])
    #plt.show()
```

## Add Baltic sea surface temperature (Warden et al. 2017)¶

In [13]:

```
#Load data
sst = pd.read_csv(inp_dir+"SI_D04_PAP_303600_TOC_LOI.tab", sep='\t')
sst.loc[::,"CE"] = (sst.loc[::,"Age [ka BP]"] - 1950) * -1
# Cut data to set time frame
maskDAT = (sst.loc[::,"CE"] >= df["CE"].min()) & (sst.loc[::,"CE"] <= df["CE"].max())
sst = sst.loc[maskDAT,::]

# write SST values into df
maskSST = df['CE'].isin(sst.loc[::,"CE"].tolist())
df.loc[maskSST,"SST"] = np.flip(sst.loc[::,"SST (1-12) [°C]"].values)

df["SST_inter"] = df["SST"].interpolate(method='linear').bfill().ffill().values
df["SST_detrend"] = signal.detrend(df["SST_inter"].values)
df["SST_growth"] = df["SST_inter"].pct_change(10).values

## Plot it
#plt.plot(df['CE'], df['SST_inter'], color = "black")
#plt.scatter(df['CE'], df["SST"], color = "black")
#plt.title("SST")
#plt.show()
```

## Save df¶

In [14]:

```
# Write ddf to .csv file
df.to_csv(out_dir+"table/super_df.csv")
```
