# Supplementary material for: Subsistence and population development from the Middle Neolithic B (2800–2350 BCE) to the Late Neolithic (2350–1700 BCE) in Southern Scandinavia
Source: PLoS One. 2024 Oct 28;19(10):e0301938. doi: 10.1371/journal.pone.0301938 (PMC11516014; doi:10.1371/journal.pone.0301938)
Supplement: S1 Supporting information — S1 File. SI_C01_SPD_KDE_models. R-script for analysing radiocarbon dates dates. The code performs the computation of over-regional and regional SPD and KDE models, as well as their export to CSV files (Rmd). S2 File. SI_C02_aoristic_dating. R-script for exporting aoristic time series derived from typochronological dated archaeological material as CSV files (Rmd). S3 File. SI_C03_vegetation_openness_score_example. R-script performing the computation of a vegetation openness score from pollen records and the export of the generated time series as CVS file (Rmd). S4 File. SI_C04_data_preparation. Jupyter Notebook performing the import and transformation of relevant data visualize plots exhibited in the paper (ipynb). S5 File. SI_C05_figures_extra. Jupyter Notebook visualizing the plots exhibited in the paper (ipynb). S1 Data. SI_D01_reg_data_no_dups. Spread sheet holding radiocarbon dates, with the information of laboratory identification, site name, geographical coordinates, site type, material, source and regional affiliation (csv). S2 Data. SI_D02_reg_axe_dagger_graves. Spread sheet holding entries of axes and daggers, with the information of context, site, parish, artefact identification, type, subtype, absolute dating, typochonological dating, references, geographical coordinates and regional affiliations (csv). S3 Data. SI_D03_pollen_example. Spread sheet holding sample entries of the pollen records from Krageholm (neotoma Site ID 3204) and Bjäresjöholmsjön (neotoma Site ID 3017) for example run of S3 File. Record can be access via the neotoma explorer (https://apps.neotomadb.org/explorer/) with their given IDs. Each entry holds the information of the records type, regional affiliation, absolute BP and BCE dating, as well as the counts of given plant taxa (csv). S4 Data. SI_D04_PAP_303600_TOC_LOI. Table holding sample entries of TOC content, LOI and SST reconstruction of sediment core PAP_303600 for correlations of population development with Baltic sea surface t [file pone.0301938.s001.zip › support_information/SI_C05_figures_extra.html]

SI\_C05\_figures\_extra


# Plot data (incl. paper figures)¶

## Preparation¶

In [1]:

```
# Load packages
import sys, os
import numpy as np
import pandas as pd

import matplotlib.pyplot as plt
from matplotlib.gridspec import GridSpec
import seaborn as sns

from scipy import signal
from scipy.stats import pearsonr
```

In [2]:

```
import warnings
warnings.filterwarnings('ignore')
```

In [3]:

```
# Add direction to script holding functions
sys.path.insert(0, './functions')
from functions import SI_C06_func_multi_plotting as mp

# 
inp_dir = "./data/data_raw/"
out_dir = "./data/data_derived/"
```

In [4]:

```
# Create list objects from files in the respective folder
meta_file_lst = os.listdir(out_dir+"meta/")
file_lst = os.listdir(out_dir+"table/")
## Inspect list object
#print(file_lst[0:4])
```

In [5]:

```
# Dating start and end in CE
dat_start = -2850
dat_end = -1699
# Information of spacing x-label (time axis) ticks
step_width = 100
label_seq = np.arange(dat_start, dat_end, step_width)
label_seq_BCE = np.arange(dat_start, dat_end, step_width)*-1
```

In [6]:

```
label_seq_BCE
```

Out[6]:

```
array([2850, 2750, 2650, 2550, 2450, 2350, 2250, 2150, 2050, 1950, 1850,
       1750])
```

In [7]:

```
df = pd.read_csv(out_dir+"table/super_df.csv")
```

In [8]:

```
temp = np.logical_and(df.loc[::,"CE"] >= dat_start, df.loc[::,"CE"] < dat_end)
```

## Over-regional SPD and KDE model¶

In [9]:

```
# Load data
spdEXP = pd.read_csv(out_dir+"table/all_all_spd_bin_exp.csv")
spdEXP["calBP"] = spdEXP["calBP"]
spdEXP["CE"] = (spdEXP["calBP"] - 1950) *-1
metaEXP = pd.read_csv(out_dir+"meta/meta_all_all_spd_bin_exp.csv")

# Calculate pos and neg deviation from model
spd_lst = [spdEXP]
neg_super, pos_super = mp.sig_dev(spd_lst)

# Plot it
fig, axs = plt.subplots(2, 1)
fig.subplots_adjust(hspace=0.75) # make a little extra space between the subplots
fig.set_size_inches(w=15,h=4) #set figure size 
ax1 = plt.subplot2grid((2,1),(0,0))

# Plot SPD and exponential model
ax1.plot(spd_lst[0].loc[temp,"CE"], spd_lst[0].loc[temp,"PrDens"], color='black', alpha=0.99, label="SPD all sites")
# Plot min-max envelop from exponential model
plt.fill_between(spd_lst[0].loc[temp,"CE"], spd_lst[0].loc[temp,"env_min"], spd_lst[0].loc[temp,"env_max"],
                color="grey", alpha=0.25, label="95% MC envelop exponential growth model")
# Iterate the negative deviations and plot them
for n in np.arange(0,len(neg_super[0])):
    if n == 0:
        plt.axvspan(neg_super[0][n][0], neg_super[0][n][1], facecolor='blue', alpha=0.15, label="negative deviation")
    else:
        plt.axvspan(neg_super[0][n][0], neg_super[0][n][1], facecolor='blue', alpha=0.15)
# Iterate the positive deviations and plot them
for p in np.arange(0,len(pos_super[0])):
    if p == 0:  
        plt.axvspan(pos_super[0][p][0], pos_super[0][p][1], facecolor='red', alpha=0.15, label="positive deviation")
    else:
        plt.axvspan(pos_super[0][p][0], pos_super[0][p][1], facecolor='red', alpha=0.15)    
ax1.set_ylabel("")
plt.grid(axis="x", alpha=0.33)
plt.ylim()
plt.xticks(label_seq, label_seq_BCE, rotation=0)
plt.xlim((dat_start, dat_end))
ax1.set_xlabel("cal. BCE")
# plt.legend(facecolor="white", loc='upper left')
plt.title("smoothed SPD all sites (binned)")
# Plot KDE
ax2 = plt.subplot2grid((2,1),(1,0))
ax2.plot(df.loc[temp,"CE"], df.loc[temp,"kde_all_mean_all"], color='black', alpha=0.99, label="cKDE (mean)")

# Plot min-max envelop from perm. test
plt.plot(df.loc[temp,"CE"], (df.loc[temp,"kde_all_mean_all"] - df.loc[temp,"kde_all_std_all"]), 
         color='black', alpha=0.5, linestyle="--", label="1-std envelop")
plt.plot(df.loc[temp,"CE"], (df.loc[temp,"kde_all_mean_all"] + df.loc[temp,"kde_all_std_all"]), 
         color='black', alpha=0.5, linestyle="--")
plt.fill_between(df.loc[temp,"CE"], 
                 (df.loc[temp,"kde_all_mean_all"] - df.loc[temp,"kde_all_std_all"]), 
                 (df.loc[temp,"kde_all_mean_all"] + df.loc[temp,"kde_all_std_all"]), 
                 color="grey", alpha=0.1)
# Compute and plot After Wanner et al. 2011 (standart deviation)
df, neg_kde, pos_kde = mp.sig_dev_super_df(df,["kde_all_detrend_all"])  
mp.plot_dev_kde(df, neg_kde, pos_kde, temp)  
ax2.set_ylabel("")
plt.grid(axis="x", alpha=0.33)
plt.ylim()
plt.xticks(label_seq, label_seq_BCE, rotation=0)
plt.xlim((dat_start, dat_end))
ax2.set_xlabel("cal. BCE")
# plt.legend(facecolor="white", loc='upper left')
plt.title("KDE model all sites")
# plt.savefig(out_dir+'figure/fig_6_region_none_spd_log_kde_all'+str(dat_start)+'_'+str(dat_end)+'.tif', dpi=600, bbox_inches="tight")
plt.savefig(out_dir+'figure/fig_6_region_none_spd_log_kde_all'+str(dat_start)+'_'+str(dat_end)+'.pdf', dpi=600, bbox_inches="tight")
plt.show()
```

## Regionalized KDE models¶

In [10]:

```
r_name = ["all","Scania and Bornholm", "Danish Isles", "Eastern Jutland", "Western Jutland"]
```

### KDE models all sites (+ growth rates)¶

In [11]:

```
# Define basics of the plot
rows = len(r_name)
cols = 2 #Hard coded as a 2 column plot is wanted
fig, axs = plt.subplots(rows, cols)
fig.subplots_adjust(hspace=0.75, wspace=0.1) #Make a little extra space between the subplots
fig.set_size_inches(w=30,h=10) #Set figure size 
# Iterate over rows and columns to plot
for row in np.arange(0,rows):
    for col in np.arange(0,cols):
        if col == 0:
            ax = plt.subplot2grid((rows,cols),(row,col))
            # Plot regional KDE
            ax.plot(df.loc[temp,"CE"], df.loc[temp,"kde_all_mean_"+r_name[row]],
                    color='black', alpha=0.99, label="KDE model all sites")
            # Plot min-max envelope
            ax.plot(df.loc[temp,"CE"], 
                    (df.loc[temp,"kde_all_mean_"+r_name[row]] - df.loc[temp,"kde_all_std_"+r_name[row]]), color='black', alpha=0.25, linestyle="--", label="1-std envelop")
            ax.plot(df.loc[temp,"CE"], 
                    (df.loc[temp,"kde_all_mean_"+r_name[row]] + df.loc[temp,"kde_all_std_"+r_name[row]]), color='black', alpha=0.25, linestyle="--")
            ax.fill_between(df.loc[temp,"CE"],
                            (df.loc[temp,"kde_all_mean_"+r_name[row]] - df.loc[temp,"kde_all_std_"+r_name[row]]),
                            (df.loc[temp,"kde_all_mean_"+r_name[row]] + df.loc[temp,"kde_all_std_"+r_name[row]]),
                            color="grey", alpha=0.1)
            # Compute and iterate the negative/positive deviations to plot them
            df, neg_kde, pos_kde = mp.sig_dev_super_df(df,["kde_all_detrend_"+r_name[row]])    
            mp.plot_dev_kde(df, neg_kde, pos_kde, temp)
            # Further plot styling
            plt.grid(axis="x", alpha=0.33)
            plt.ylim(ymin=0)
            plt.xticks(label_seq, label_seq_BCE, rotation=0)
            plt.xlim((dat_start, dat_end))
            plt.title("KDE model: " + r_name[row])
            # Plot CE x lab only on last plot in row
            if row == rows-1:
                ax.set_xlabel("cal. BCE")
            else:
                pass
        else:
            ax = plt.subplot2grid((rows,cols),(row,col))
            # Plot regional KE
            ax.plot(df.loc[temp,"CE"],df.loc[temp,"kde_all_mean_"+r_name[row]].pct_change(periods=10)
                    , color='black', alpha=0.99, label="KDE model all sites growth rate")
            # Plot min-max envelope
            ax.plot(df.loc[temp,"CE"], 
                    (df.loc[temp,"kde_all_mean_"+r_name[row]] - df.loc[temp,"kde_all_std_"+r_name[row]]).pct_change(periods=10), color='black', alpha=0.25, linestyle="--", label="1-std envelop")
            ax.plot(df.loc[temp,"CE"], 
                    (df.loc[temp,"kde_all_mean_"+r_name[row]] + df.loc[temp,"kde_all_std_"+r_name[row]]).pct_change(periods=10), color='black', alpha=0.25, linestyle="--")
            ax.fill_between(df.loc[temp,"CE"],
                            (df.loc[temp,"kde_all_mean_"+r_name[row]] - df.loc[temp,"kde_all_std_"+r_name[row]]).pct_change(periods=10), 
                            (df.loc[temp,"kde_all_mean_"+r_name[row]] + df.loc[temp,"kde_all_std_"+r_name[row]]).pct_change(periods=10),
                            color="grey", alpha=0.1)
            ax.hlines(0,-2850,-1700,color="black",ls=":")
            # Compute and iterate the negative/positive deviations to plot them
            df, neg_kde, pos_kde = mp.sig_dev_super_df(df,["kde_all_growth_"+r_name[row]])     
            mp.plot_dev_kde(df, neg_kde, pos_kde, temp)
            # Further plot styling
            ax.set_ylabel("growth rate")
            plt.grid(axis="x", alpha=0.33)
            plt.ylim()
            plt.xticks(label_seq, label_seq_BCE, rotation=0)
            plt.xlim((dat_start, dat_end))
            plt.title("Growth rate: "+r_name[row])
            # Plot CE x lab only on last plot in row
            if row == rows-1:
                ax.set_xlabel("cal. BCE")
            else:
                pass  
# fig.legend(loc="lower center")
# plt.savefig(out_dir+'figure/fig_7_kde_all_vs_growth_all.tif', dpi=600, bbox_inches="tight")
plt.savefig(out_dir+'figure/fig_7_kde_all_vs_growth_all.pdf', dpi=600, bbox_inches="tight")
plt.show()
```

### KDE models settlement vs. burial contexts¶

In [12]:

```
# Define basics of the plot
rows = len(r_name)
cols = 2 #Hard coded as a 2 column plot is wanted
fig, axs = plt.subplots(rows, cols)
fig.subplots_adjust(hspace=0.75, wspace=0.1) #Make a little extra space between the subplots
fig.set_size_inches(w=30,h=10) #Set figure size 
# Iterate over rows and columns to plot
for row in np.arange(0,rows):
    for col in np.arange(0,cols):
        if col == 0:
            ax = plt.subplot2grid((rows,cols),(row,col))
            # Plot regional KDE
            ax.plot(df.loc[temp,"CE"], df.loc[temp,"kde_set_mean_"+r_name[row]],
                    color='black', alpha=0.99, label="KDE model settlement sites")
            # Plot min-max envelope
            ax.plot(df.loc[temp,"CE"], 
                    (df.loc[temp,"kde_set_mean_"+r_name[row]] - df.loc[temp,"kde_set_std_"+r_name[row]]), color='black', alpha=0.25, linestyle="--", label="1-std envelop")
            ax.plot(df.loc[temp,"CE"], 
                    (df.loc[temp,"kde_set_mean_"+r_name[row]] + df.loc[temp,"kde_set_std_"+r_name[row]]), color='black', alpha=0.25, linestyle="--")
            ax.fill_between(df.loc[temp,"CE"],
                            (df.loc[temp,"kde_set_mean_"+r_name[row]] - df.loc[temp,"kde_set_std_"+r_name[row]]),
                            (df.loc[temp,"kde_set_mean_"+r_name[row]] + df.loc[temp,"kde_set_std_"+r_name[row]]),
                            color="grey", alpha=0.1)
            # Compute and iterate the negative/positive deviations to plot them
            df, neg_kde, pos_kde = mp.sig_dev_super_df(df,["kde_set_detrend_"+r_name[row]])     
            mp.plot_dev_kde(df, neg_kde, pos_kde, temp)
            # Further plot styling
            plt.grid(axis="x", alpha=0.33)
            plt.ylim(ymin=0)
            plt.xticks(label_seq, label_seq_BCE, rotation=0)
            plt.xlim((dat_start, dat_end))
            plt.title("KDE model settlement sites: " + r_name[row])
            # Plot CE x lab only on last plot in row
            if row == rows-1:
                ax.set_xlabel("cal. CE")
            else:
                pass
        else:
            ax = plt.subplot2grid((rows,cols),(row,col))
            # Plot regional KE
            ax.plot(df.loc[temp,"CE"],df.loc[temp,"kde_bur_mean_"+r_name[row]]
                    , color='black', alpha=0.99, label="KDE model burial sites")
            # Plot min-max envelope
            ax.plot(df.loc[temp,"CE"], 
                    (df.loc[temp,"kde_bur_mean_"+r_name[row]] - df.loc[temp,"kde_bur_std_"+r_name[row]]), color='black', alpha=0.25, linestyle="--", label="1-std envelop")
            ax.plot(df.loc[temp,"CE"], 
                    (df.loc[temp,"kde_bur_mean_"+r_name[row]] + df.loc[temp,"kde_bur_std_"+r_name[row]]), color='black', alpha=0.25, linestyle="--")
            ax.fill_between(df.loc[temp,"CE"],
                            (df.loc[temp,"kde_bur_mean_"+r_name[row]] - df.loc[temp,"kde_bur_std_"+r_name[row]]), 
                            (df.loc[temp,"kde_bur_mean_"+r_name[row]] + df.loc[temp,"kde_bur_std_"+r_name[row]]),
                            color="grey", alpha=0.1)
            ax.hlines(0,-2850,-1700,color="black",ls=":")
            # Compute and iterate the negative/positive deviations to plot them
            df, neg_kde, pos_kde = mp.sig_dev_super_df(df,["kde_bur_detrend_"+r_name[row]])     
            mp.plot_dev_kde(df, neg_kde, pos_kde, temp)
            # Further plot styling
            ax.set_ylabel("")
            plt.grid(axis="x", alpha=0.33)
            plt.ylim()
            plt.xticks(label_seq, label_seq_BCE, rotation=0)
            plt.xlim((dat_start, dat_end))
            plt.title("KDE model burial sites: "+r_name[row])
            # Plot CE x lab only on last plot in row
            if row == rows-1:
                ax.set_xlabel("cal. CE")
            else:
                pass  
# fig.legend(loc="lower center")
# plt.savefig(out_dir+'figure/kde_set_vs_bur.pdf', dpi=600, bbox_inches="tight")
plt.show()
```

### KDE models correlations¶

In [13]:

```
dat_all = (-2850,-1700)
dat_mnb = (-2850,-2350)
dat_ln = (-2350,-1700)
# Write the tuples into a list
dat_lst = [dat_all, dat_mnb, dat_ln]
```

#### Pearson correlation¶

In [14]:

```
x_name = r_name[1:]
mp.plot_c_cor_reg_reg(df, dat_lst, x_name, method_tst = "detrend")
mp.plot_c_cor_reg_reg(df, dat_lst, x_name, method_tst = "growth")
```

#### Auto- and cross-correlations¶

In [15]:

```
# Define length of time lag (in years)
lag = 300
# - years: X schneller als Y :: + years: X langsamer als Y

# Define colors
color_lst = ["blue","red","green","orange","purple"]
```

In [16]:

```
for var in ["detrend", "growth"]:
    for ent in np.arange(0,len(dat_lst)):
        # Subset df to correlation window min and max date
        tempX = np.logical_and(df.loc[::,"CE"] >= dat_lst[ent][0], 
                               df.loc[::,"CE"] < dat_lst[ent][1])
        fig = plt.figure(figsize=(int(len(r_name)*2), int(len(r_name)*2)))
        fig.subplots_adjust(hspace=0.25, wspace = 0.25)
        fig.suptitle("Cross-correlation regional KDE models ("+ var + ") : "+\
                     str(dat_lst[ent][0]*-1)+'-'+str(dat_lst[ent][1]*-1)+" BCE", fontsize=12)
        gs = GridSpec(nrows=len(r_name), ncols=len(r_name))
        for r1 in np.arange(0,len(r_name)):
            for r2 in np.arange(0,len(r_name)):
                if (r1 == r1 and r2 > r1):
                    pass
                else:
                    ax = fig.add_subplot(gs[r1,r2])
                    d1 = signal.detrend(df.loc[tempX,"kde_all_"+var+"_"+r_name[r1]])
                    d2 = signal.detrend(df.loc[tempX,"kde_all_"+var+"_"+r_name[r2]])
                    a = plt.xcorr(d1, d2, usevlines=True, normed=True, maxlags=lag, lw=1, color=color_lst[r1]) 
                    # Compute significance level
                    plt.hlines((2./np.sqrt(lag)), -lag, lag, "k", linestyle=":", alpha=0.5)
                    plt.hlines((2./np.sqrt(lag))*-1, -lag, lag, "k", linestyle=":", alpha=0.5)
                    plt.xticks(fontsize = 7)
                    plt.yticks(fontsize = 7)
                    if r2 == 0:
                        plt.ylabel(r_name[r1]+" \n correlation",
                                   fontsize = 8)
                    else:
                        pass 
                    if r1 == len(r_name)-1:
                        plt.xlabel("lag (in years)",fontsize = 8)
                    else:
                        pass
                    if (r1 == r1 and r2 == r1):
                        plt.title(r_name[r2], fontsize= 8, pad=20, fontweight="bold")
                    else:
                        pass
                    plt.ylim(-1,1)
                    plt.xlim(0,300)
                    plt.grid(alpha=0.33) 
        #plt.savefig(out_dir+'figure/cross_cor_kde_'+var+'_'+str(dat_lst[ent][0])+'_'+str(dat_lst[ent][1])+'.tif', dpi=600, bbox_inches="tight")
        plt.savefig(out_dir+'figure/cross_cor_kde_'+var+'_'+str(dat_lst[ent][0])+'_'+str(dat_lst[ent][1])+'.pdf', dpi=600, bbox_inches="tight")
        plt.show()
```

## Aoristic time series¶

In [17]:

```
# Load data
dfAO = pd.read_csv(out_dir+"table/region_geom_2_ao_da.csv")
ao_name = r_name = ["all", "Danish Isles", "Eastern Jutland", "Western Jutland"]
tempAO = np.logical_and(dfAO.loc[::,"CE"] >= dat_start, dfAO.loc[::,"CE"] < dat_end)

# Define basics of the plot
rows = len(ao_name)
cols = 1 # Hard coded as a 2 column plot is wanted
fig, axs = plt.subplots(rows, cols)
fig.subplots_adjust(hspace=0.75) # Make a little extra space between the subplots
fig.set_size_inches(w=15,h=8) # Set figure size 
# Iterate over rows and columns to plot
for row in np.arange(0,rows):
    ax = plt.subplot2grid((rows,1),(row,0))
    # Plot regional KDE
    ax.plot(dfAO.loc[tempAO,"CE"], dfAO.loc[tempAO,"dagger_"+r_name[row]],
            color='purple', alpha=0.99, label="Dagger", lw=2, linestyle="--")
    ax.plot(dfAO.loc[tempAO,"CE"], dfAO.loc[tempAO,"axe_"+r_name[row]],
            color='green', alpha=0.99, label="Axe", lw=2)
    plt.grid(axis="x", alpha=0.33)
    plt.ylim(ymin=0)
    plt.xticks(label_seq, label_seq_BCE, rotation=0)
    plt.xlim((dat_start, dat_end))
    plt.title("Aoristic sum: " + ao_name[row])
    # Plot CE x lab only on last plot in row
    if row == rows-1:
        ax.set_xlabel("BCE")
# plt.savefig(out_dir+'figure/fig_8_axe_dagger.tif', dpi=600, bbox_inches="tight")
plt.savefig(out_dir+'figure/fig_8_axe_dagger.pdf', dpi=600, bbox_inches="tight")
plt.show()
```

## Regional vegetation openness¶

### Vegetation openness scores (+growth rates)¶

In [18]:

```
vos_name = ["Northern Jutland", "Zealand", "Southern Jutland"]

# Define basics of the plot
rows = len(vos_name)
cols = 2 # Hard coded as a 2 column plot is wanted
fig, axs = plt.subplots(rows, cols)
fig.subplots_adjust(hspace=0.75, wspace=0.1) # Make a little extra space between the subplots
fig.set_size_inches(w=30,h=6) # Set figure size 
# Iterate over rows and columns to plot
for row in np.arange(0,rows):
    for col in np.arange(0,cols):
        if col == 0:
            ax = plt.subplot2grid((rows,cols),(row,col))
            # Plot regional KDE
            ax.plot(df.loc[temp,"CE"], df.loc[temp,"VOS_mean_"+vos_name[row]+"_inter"],
                    color='black', alpha=0.99, label="VOS")
            
            if vos_name[row] != "Southern Jutland":
                # Plot min-max envelope
                ax.fill_between(df.loc[temp,"CE"],
                                (df.loc[temp,"VOS_mean_"+vos_name[row]+"_inter"] - df.loc[temp,"VOS_std_"+vos_name[row]+"_inter"]),
                                (df.loc[temp,"VOS_mean_"+vos_name[row]+"_inter"] + df.loc[temp,"VOS_std_"+vos_name[row]+"_inter"]),
                                color="grey", alpha=0.1)
                if vos_name[row] == "Zealand":
                    ax.plot(df.loc[temp,"CE"], df.loc[temp,"VOS_mean_Vinge_inter"],
                            color='red', alpha=0.75, label="VOS Vinge")
                else:
                    pass
            else:
                pass
                
            # Compute and iterate the negative/positive deviations to plot them
            df, neg_vos, pos_vos = mp.sig_dev_super_df(df,["VOS_mean_"+vos_name[row]+"_detrend"])    
            mp.plot_dev_vos(df, neg_vos, pos_vos, temp)
            
            # Further plot styling
            plt.grid(axis="x", alpha=0.33)
            plt.xticks(label_seq, label_seq_BCE, rotation=0)
            plt.xlim((dat_start, dat_end))
            plt.title("Vegetation openness score: " + vos_name[row])
            # Plot CE x lab only on last plot in row
            if row == rows-1:
                ax.set_xlabel("cal. BCE")
            else:
                pass
        else:
            ax = plt.subplot2grid((rows,cols),(row,col))
            # Plot regional KE
            ax.plot(df.loc[temp,"CE"],df.loc[temp,"VOS_mean_"+vos_name[row]+"_growth"]
                    , color='black', alpha=0.99, label="VOS growth rate")
            ax.hlines(0,-2850,-1700,color="black",ls=":")
            
            # Compute and iterate the negative/positive deviations to plot them
            df, neg_vos, pos_vos = mp.sig_dev_super_df(df,["VOS_mean_"+vos_name[row]+"_growth"])     
            mp.plot_dev_vos(df, neg_vos, pos_vos, temp)
            
            # Further plot styling
            ax.set_ylabel("growth rate")
            plt.grid(axis="x", alpha=0.33)
            plt.ylim()
            plt.xticks(label_seq, label_seq_BCE, rotation=0)
            plt.xlim((dat_start, dat_end))
            plt.title("Growth rate: "+vos_name[row])
            # Plot CE x lab only on last plot in row
            if row == rows-1:
                ax.set_xlabel("cal. BCE")
            else:
                pass  
            
            if vos_name[row] == "Zealand":
                
                ax2 = ax.twinx()
                ax2.plot(df.loc[temp,"CE"], df.loc[temp,"VOS_mean_Vinge_growth"],
                        color='red', alpha=0.75, label="VOS Vinge")
                ax2.set_ylabel("growth rate (Vinge)", c='red')
                ax2.hlines(0,-2850,-1700,color="red",ls=":")
            else:
                pass
            
# fig.legend(loc="lower center")
# plt.savefig(out_dir+'figure/fig_9_vos_model_vs_growth.tif', dpi=600, bbox_inches="tight")
plt.savefig(out_dir+'figure/fig_9_vos_model_vs_growth.pdf', dpi=600, bbox_inches="tight")
plt.show()
```

### KDE models all sites (+ growth rates) of pollen reference regions¶

In [19]:

```
# Define basics of the plot
rows = len(vos_name)
cols = 2 #Hard coded as a 2 column plot is wanted
fig, axs = plt.subplots(rows, cols)
fig.subplots_adjust(hspace=0.5, wspace=0.1) #Make a little extra space between the subplots
fig.set_size_inches(w=30,h=6) #Set figure size 
# Iterate over rows and columns to plot
for row in np.arange(0,rows):
    for col in np.arange(0,cols):
        if col == 0:
            ax = plt.subplot2grid((rows,cols),(row,col))
            # Plot regional KDE
            ax = plt.subplot2grid((rows,cols),(row,col))
            # Plot regional KE
            ax.plot(df.loc[temp,"CE"],df.loc[temp,"kde_all_mean_"+vos_name[row]]
                    , color='black', alpha=0.99, label="KDE model all sites")
            # Plot min-max envelope
            ax.plot(df.loc[temp,"CE"], 
                    (df.loc[temp,"kde_all_mean_"+vos_name[row]] - df.loc[temp,"kde_all_std_"+vos_name[row]]), color='black', alpha=0.25, linestyle="--", label="1-std envelop")
            ax.plot(df.loc[temp,"CE"], 
                    (df.loc[temp,"kde_all_mean_"+vos_name[row]] + df.loc[temp,"kde_all_std_"+vos_name[row]]), color='black', alpha=0.25, linestyle="--")
            ax.fill_between(df.loc[temp,"CE"],
                            (df.loc[temp,"kde_all_mean_"+vos_name[row]] - df.loc[temp,"kde_all_std_"+vos_name[row]]), 
                            (df.loc[temp,"kde_all_mean_"+vos_name[row]] + df.loc[temp,"kde_all_std_"+vos_name[row]]),
                            color="grey", alpha=0.1)
            # Compute and iterate the negative/positive deviations to plot them
            df, neg_kde, pos_kde = mp.sig_dev_super_df(df,["kde_all_detrend_"+vos_name[row]])    
            mp.plot_dev_kde(df, neg_kde, pos_kde, temp) 
            # Further plot styling
            plt.ylim(0)
            plt.grid(axis="x", alpha=0.33)
            plt.xticks(label_seq, rotation=0)
            plt.xlim((dat_start, dat_end))
            plt.title("KDE model: " + vos_name[row])
            # Plot CE x lab only on last plot in row
            if row == rows-1:
                ax.set_xlabel("cal. CE")
            else:
                pass
        else:
            ax = plt.subplot2grid((rows,cols),(row,col))
            # Plot regional KE
            ax.plot(df.loc[temp,"CE"],df.loc[temp,"kde_all_mean_"+vos_name[row]].pct_change(periods=10)
                    , color='black', alpha=0.99, label="KDE model all sites growth rate")
            # Plot min-max envelope
            ax.plot(df.loc[temp,"CE"], 
                    (df.loc[temp,"kde_all_mean_"+vos_name[row]] - df.loc[temp,"kde_all_std_"+vos_name[row]]).pct_change(periods=10), color='black', alpha=0.25, linestyle="--", label="1-std envelop")
            ax.plot(df.loc[temp,"CE"], 
                    (df.loc[temp,"kde_all_mean_"+vos_name[row]] + df.loc[temp,"kde_all_std_"+vos_name[row]]).pct_change(periods=10), color='black', alpha=0.25, linestyle="--")
            ax.fill_between(df.loc[temp,"CE"],
                            (df.loc[temp,"kde_all_mean_"+vos_name[row]] - df.loc[temp,"kde_all_std_"+vos_name[row]]).pct_change(periods=10), 
                            (df.loc[temp,"kde_all_mean_"+vos_name[row]] + df.loc[temp,"kde_all_std_"+vos_name[row]]).pct_change(periods=10),
                            color="grey", alpha=0.1)
            ax.hlines(0,-2850,-1700,color="black",ls=":")
            # Compute and iterate the negative/positive deviations to plot them
            df, neg_kde, pos_kde = mp.sig_dev_super_df(df,["kde_all_growth_"+vos_name[row]])     
            mp.plot_dev_kde(df, neg_kde, pos_kde, temp) 
            # Further plot styling
            ax.set_ylabel("growth rate")
            plt.grid(axis="x", alpha=0.33)
            plt.ylim()
            plt.xticks(label_seq, label_seq_BCE, rotation=0)
            plt.xlim((dat_start, dat_end))
            plt.title("Growth rate: "+vos_name[row])
            # Plot CE x lab only on last plot in row
            if row == rows-1:
                ax.set_xlabel("cal. BCE")
            else:
                pass  
# fig.legend(loc="lower center")
# plt.savefig(out_dir+'figure/fig_10_kde_model_vs_growth_all_geom3.tif', dpi=600, bbox_inches="tight")
plt.savefig(out_dir+'figure/fig_10_kde_model_vs_growth_all_geom3.pdf', dpi=600, bbox_inches="tight")
plt.show()
```

### VOS vs KDE model correlation¶

#### Pearson correlation (binned data)¶

In [20]:

```
mp.plot_b_cor_vos_kde(df, dat_lst, vos_name, method_tst = "detrend")
mp.plot_b_cor_vos_kde(df, dat_lst, vos_name, method_tst = "growth")
```

#### Auto- and cross-correlation (binned data)¶

In [21]:

```
for var in ["detrend", "growth"]:
    fig = plt.figure(figsize=(int(len(vos_name)*2), int(len(vos_name)*2)))
    fig.subplots_adjust(hspace=0.5, wspace=0.5)
    fig.suptitle("Cross-correlation VOS vs. binned KDE models ("+var+")", fontsize=12)
    
    gs = GridSpec(nrows=len(dat_lst), ncols=len(vos_name))
    color_lst = ["orange","blue","green"]
    bin_lst = []
    for d in np.arange(0,len(dat_lst)):
        # Iterare over all "relevant" proxies
        tempX = np.logical_and(df.loc[::,"CE"] >= dat_lst[d][0],
                              df.loc[::,"CE"] < dat_lst[d][1])

        for p in np.arange(0,len(vos_name)):
            # -----------------------------------------------------------------------------
            # Find start and end date index in df of given proxy  -------------------------
            # -----------------------------------------------------------------------------
            mask1 = pd.notna(df.loc[::,"VOS_mean_"+vos_name[p]])
            x_df = df.loc[mask1,["VOS_mean_"+vos_name[p],"CE"]]
            x_df = x_df.loc[tempX,::]
            x_lst = []
            # ----------------------------------------------------------------------------- 
            # Iterate over subset df and measure the distance of each dating 
            # its predating date
            for i in np.arange(0,len(list(x_df.index))):
                if i == len(list(x_df.index))-1:
                    pass
                else:
                    x_lst.append(x_df.loc[list(x_df.index)[i],"CE"] - \
                                 x_df.loc[list(x_df.index)[i+1],"CE"])

            # Calculate a max (here min, because of negative chronological 
            # scheme) distance between measures with a date and assigne a 
            # "good" bin size for "Palmisano correlation".
            if (np.min(x_lst)*-1) <= 25:
                binS = 25
            elif 25 < (np.min(x_lst)*-1) <= 50:
                binS = 50
            elif 50 < (np.min(x_lst)*-1) <= 100:
                binS = 100
            elif 100 < (np.min(x_lst)*-1) <= 150:
                binS = 150
            elif 150 < (np.min(x_lst)*-1) <= 200:
                binS = 200  
            elif 200 < (np.min(x_lst)*-1) <= 250:
                binS = 250
            elif 250 < (np.min(x_lst)*-1) <= 300:
                binS = 300
            else:
                binS = 0
            # ----------------------------------------------------------------------------- 
            
            s_idx = df.loc[::,"CE"][df.loc[::,"CE"] == dat_lst[d][0]].index.values[0]
            e_idx = df.loc[::,"CE"][df.loc[::,"CE"] == dat_lst[d][1]].index.values[0]        
            # Prepare breaks and window for binned window correlation
            # write the tuples into a list
            if binS > 0:
                brks = np.arange(s_idx, e_idx, binS)
                # Iterate over regions to calculate correlation between proxy 
                #Correlate bins over full duration of proxy
                prox_bin = []
                regi_bin = []
                for j in brks:
                    prox_bin.append(
                        df.loc[j:j+binS,"VOS_mean_"+vos_name[p]+"_"+var].mean(skipna=True))
                    # mean
                    regi_bin.append(
                        df.loc[j:j+binS,"kde_all_"+var+"_"+vos_name[p]].mean(skipna=True))

                bin_lst.append((binS, len(prox_bin)))

                lag = int(np.round(len(prox_bin) / 3, 0))

                ax = fig.add_subplot(gs[d,p])
                if len(color_lst) == 0:
                    a = ax.xcorr(prox_bin, regi_bin, usevlines=True, normed=True, maxlags=lag, lw=2)
                else:
                    a = ax.xcorr(prox_bin, regi_bin, usevlines=True, normed=True, maxlags=lag, lw=2, color = color_lst[p])
                plt.hlines((2./np.sqrt(lag)), -lag, lag, "k", linestyle=":", alpha=0.5)
                plt.hlines((2./np.sqrt(lag))*-1, -lag, lag, "k", linestyle=":", alpha=0.5)
                plt.xticks(fontsize = 8)
                plt.yticks(fontsize = 8)
                if p == 0:
                    plt.ylabel(str(dat_lst[d][0]*-1)+"-"+str(dat_lst[d][1]*-1)+" BCE \n correlation",
                               fontsize= 10)
                else:
                    pass 
                if d == len(dat_lst)-1:
                    plt.xlabel("lag (in bins a "+str(bin_lst[p][0])+" years)",
                               fontsize= 10)
                else:
                    pass
                if d == 0:
                    plt.title(vos_name[p], fontsize= 10, pad=20, fontweight="bold")
                else:
                    pass
                plt.ylim(-1,1)
                plt.grid(alpha=0.5)
            else:
                pass
    #plt.savefig(out_dir+'figure/cross_cor_vos_detrend_'+str(dat_lst[ent][0])+'_'+str(dat_lst[ent][1])+'.tif', dpi=600, bbox_inches="tight")
    plt.savefig(out_dir+'figure/cross_cor_vos_detrend_'+str(dat_lst[ent][0])+'_'+str(dat_lst[ent][1])+'.pdf', dpi=600, bbox_inches="tight")
    plt.show()
```

## Detour: Pearson correlation Baltic sea surface temerature vs. regional KDE models¶

In [22]:

```
for var in ["detrend", "growth"]:
    dat_str_lst = []
    for t in np.arange(0,len(dat_lst)):
        dat_str_lst.append(str(dat_lst[t][0]*-1)+"-"+str(dat_lst[t][1]*-1)+" BCE")
    dfCOR = pd.DataFrame(index=dat_str_lst, columns=r_name) 
    dfSIG = pd.DataFrame(index=dat_str_lst, columns=r_name) 


    for d in np.arange(0,len(dat_lst)):
        # Iterare over all "relevant" proxies

        temp = np.logical_and(df.loc[::,"CE"] >= dat_lst[d][0],
                              df.loc[::,"CE"] < dat_lst[d][1])

        bin_lst = []

        # -----------------------------------------------------------------------------
        # Find start and end date index in df of given proxy  -------------------------
        # -----------------------------------------------------------------------------
        mask1 = pd.notna(df.loc[::,"SST"])
        x_df = df.loc[mask1,["SST","CE"]]
        x_df = x_df.loc[temp,::]
        x_lst = []
        # ----------------------------------------------------------------------------- 
        # Iterate over subset df and measure the distance of each dating 
        # its predating date
        for i in np.arange(0,len(list(x_df.index))):
            if i == len(list(x_df.index))-1:
                pass
            else:
                x_lst.append(x_df.loc[list(x_df.index)[i],"CE"] - \
                             x_df.loc[list(x_df.index)[i+1],"CE"])

        # Calculate a max (here min, because of negative chronological 
        # scheme) distance between measures with a date and assigne a 
        # "good" bin size for "Palmisano correlation".
        if (np.min(x_lst)*-1) <= 25:
            binS = 25
        elif 25 < (np.min(x_lst)*-1) <= 50:
            binS = 50
        elif 50 < (np.min(x_lst)*-1) <= 100:
            binS = 100
        elif 100 < (np.min(x_lst)*-1) <= 150:
            binS = 150
        elif 150 < (np.min(x_lst)*-1) <= 200:
            binS = 200  
        elif 200 < (np.min(x_lst)*-1) <= 250:
            binS = 250
        elif 250 < (np.min(x_lst)*-1) <= 300:
            binS = 300
        else:
            binS = 0
        # ----------------------------------------------------------------------------- 

        for p in np.arange(0,len(r_name)):
            s_idx = df.loc[::,"CE"][df.loc[::,"CE"] == dat_lst[d][0]].index.values[0]
            e_idx = df.loc[::,"CE"][df.loc[::,"CE"] == dat_lst[d][1]].index.values[0]        

            # Prepare breaks and window for binned window correlation
            # write the tuples into a list
            if binS > 0:
                brks = np.arange(s_idx, e_idx, binS)

                # Correlate bins over full duration of proxy
                prox_bin = []
                regi_bin = []
                for j in brks:
                    prox_bin.append(df.loc[j:j+binS,"SST_"+var].mean(skipna=True))
                    #!!! mean, sum, was?
                    regi_bin.append(
                        df.loc[j:j+binS,"kde_all_"+var+"_"+r_name[p]].mean(skipna=True))
                # Write correlation (full len) and significance into given 
                # cells of dfs
                dfCOR.loc[dat_str_lst[d],r_name[p]] = pearsonr(prox_bin,regi_bin)[0]
                dfSIG.loc[dat_str_lst[d],r_name[p]] = pearsonr(prox_bin,regi_bin)[1]
            else:
                pass

            bin_lst.append((binS, len(prox_bin)))

        # Make all coulmns to dtype numeric
        for col in dfCOR.columns.tolist():
            dfCOR[col] = pd.to_numeric(dfCOR[col])
            dfSIG[col] = pd.to_numeric(dfSIG[col])

    sns.heatmap(dfCOR, square=True, annot=True, vmin=-1, vmax=1, center=0, 
                linewidths=.5, cmap='PRGn_r', cbar_kws={"shrink": .85}).set(
                    title="Correlation SST vs. binned KDE models ("+var+")")
    plt.savefig(out_dir+'figure/cor_sst_kde_'+var+'.png', dpi=500, bbox_inches="tight")
    plt.show()
```
